# Modeling dynamics of acute HIV infection incorporating density-dependent cell death and multiplicity of infection

**DOI:** 10.1371/journal.pcbi.1012129

**Published:** 2024-06-07

**Authors:** Ellie Mainou, Ruy M. Ribeiro, Jessica M. Conway

**Affiliations:** 1 Department of Biology, Center for Infectious Disease Dynamics, The Pennsylvania State University, University Park, Pennsylvania, United States of America; 2 Theoretical Biology and Biophysics, Los Alamos National Laboratory, Los Alamos, New Mexico, United States of America; 3 Department of Mathematics, Center for Infectious Disease Dynamics, The Pennsylvania State University, University Park, Pennsylvania, United States of America; Utrecht University, NETHERLANDS

## Abstract

Understanding the dynamics of acute HIV infection can offer valuable insights into the early stages of viral behavior, potentially helping uncover various aspects of HIV pathogenesis. The standard viral dynamics model explains HIV viral dynamics during acute infection reasonably well. However, the model makes simplifying assumptions, neglecting some aspects of HIV infection. For instance, in the standard model, target cells are infected by a single HIV virion. Yet, cellular multiplicity of infection (MOI) may have considerable effects in pathogenesis and viral evolution. Further, when using the standard model, we take constant infected cell death rates, simplifying the dynamic immune responses. Here, we use four models—1) the standard viral dynamics model, 2) an alternate model incorporating cellular MOI, 3) a model assuming density-dependent death rate of infected cells and 4) a model combining (2) and (3)—to investigate acute infection dynamics in 43 people living with HIV very early after HIV exposure. We find that all models qualitatively describe the data, but none of the tested models is by itself the best to capture different kinds of heterogeneity. Instead, different models describe differing features of the dynamics more accurately. For example, while the standard viral dynamics model may be the most parsimonious across study participants by the corrected Akaike Information Criterion (AICc), we find that viral peaks are better explained by a model allowing for cellular MOI, using a linear regression analysis as analyzed by *R*^2^. These results suggest that heterogeneity in within-host viral dynamics cannot be captured by a single model. Depending on the specific aspect of interest, a corresponding model should be employed.

## Author summary

We conducted a study to better understand the dynamics of early HIV-1 infection using four different mathematical models. These models tested various biological hypotheses, including the presence of cellular coinfection and phenomenological model capturing aspects of immune responses. We analyzed viral load data from 43 participants in a rich dataset of people recently infected. The models we used were the standard viral dynamics model, a model incorporating density-dependent cell death of infected cells, a model with coinfection of infected cells (an adaptation of a macroparasite model to HIV-1 dynamics), and a model that combined cellular coinfection and density-dependent cell death. Overall, the model incorporating density-dependent cell death of infected cells performed the best according to the Akaike Information Criterion (AIC) and Bayesian Information Criterion (BIC). We also assessed the models’ performance by comparing them based on measures such as viral growth rate, peak magnitude and timing, decay rate, and setpoint. We find that different models describe differing features of the dynamics more accurately. For example, viral peaks are better explained by a model allowing for cellular multiplicity of infection (MOI). These results suggest that heterogeneity in within-host viral dynamics cannot be captured by a single model. Therefore, depending on the aspect of interest, a corresponding appropriate model should be employed.

## Introduction

HIV-1 remains a global health challenge, with 38.4 million people living with HIV in 2021 [1]. To control HIV-1 incidence, a successful vaccine is likely needed [2]. However, candidate vaccines tested so far have failed or provide modest efficacy [3]. Elucidating the key events that occur during acute infection may facilitate the development of effective vaccines. Here, we aim to better characterize the dynamics of early HIV-1 infection by testing four different mechanistic hypotheses; specifically, we aim to investigate the effects of cellular coinfection and immune responses on acute HIV infection dynamics.

Acute HIV infection covers a period of 4–5 weeks in which the virus disseminates from the initial site of infection into various tissues and organs [4]. Primary infection kinetics are characterized by exponential increase in the number of virus particles in peripheral blood, reaching a peak, followed by a decline to steady state level, which is referred to as the viral setpoint [5–7]. The viral peak coincides with the first appearance of an adaptive immune response [8]. The decline in plasma viremia is attributed to adaptive immune responses [8], and/or to target cell limitation [9, 10].

Mathematical models have helped to better understand the processes driving within-host dynamics of viral infections. In the case of HIV, acute infection has been modeled with a standard model of viral dynamics initially developed to explore viral decay during treatment [11, 12]. The model consists of uninfected target cells (CD4+ T cells), infected cells and free plasma virus [12]. A central assumption of the model is that target cells are either uninfected or infected with a single virion. Fitting of this model to viral load measurements has yielded estimates for the within-host basic reproductive number *R*_0_ [13], as well as estimates for key parameters [10–12, 14–17]. Overall, the standard model has been able to capture viral loads in people living with HIV-1 early in infection and provide insight into viral kinetics following treatment initiation [9–12]. However, fits to viral load measurements underestimates peak viremia and produce oscillatory dynamics during the setpoint [18]. In addition, certain important assumptions are made. First, the model assumed a constant, log-linear decay of the infected cell population. While this assumption may be valid for the early stages of the infection, it might not be realistic for longer periods of months or years. As the infection is established, the increased presence of infected cells may stimulate immune responses that could lead to an increased killing of infected cells, modeled phenomenologically by Holte et al. (2006) [19]. Reeves et al (2021) were the first to fit an ensemble of models to a rich data set of acute HIV-1 infection (RV217 data set) [20] using a population non-linear mixed-effects framework and found that the standard model incorporating the density dependent cell-death term developed by Holte et al. provides the best fit at the population level [21]. Several models have explicitly integrated adaptive immune responses [22–27]; for a review see [28].

A potentially important infection mechanism not included in the standard model is cellular multiplicity of infection, which may have considerable effects in pathogenesis and evolution. Cellular coinfection can allow for the generation of recombinant viruses. For example, Levy et al. (2004) found that a single round of replication in T lymphocytes in culture generated an average of nine recombination events per virus [29]. Genetic recombination may assist HIV-1 diversification and escape from both antiviral therapies and host immune responses [29]. More recent work shows that recombinant genomes can replace transmitted/founder lineages with a median half-time of 27 days, and more importantly, recombination facilitates the propagation of mutations that confer resistance within the evolving viral population [30, 31]. The effects of cellular MOI have been explored in qualitative analyses of mathematical models for viral infectious diseases [32]. Dixit & Perelson (2004 and 2005) developed a model for HIV cellular coinfection [18, 33], whose central assumption is that viral production remains constant across infected cells, regardless of multiplicity of infection. Under this scenario, predictions of this model are identical to the standard viral dynamics model. However, if multiply infected cells are characterized by an increased burst size, predictions are altered. In this case, the establishment of infection may not be dependent on the basic reproduction number only, but also on the initial virus load. In addition, the viral population may not grow exponentially at a constant rate, but in an increasing rate as viral load increases [34]. Whether burst size is constant across different classes of infected cells remains unknown [29]. Wodarz & Levy (2011) and Guo et al. (2021) developed a system of ODEs, where each class of infected cells is represented by an equation. These models were used to make theoretical predictions and were not fit to data, to our knowledge [35, 36]. HIV-1 models that account for multiplicity of infection remain high-dimensional and difficult to be validated with observed viral load datasets (see Discussion section).

To account for cellular coinfection without resorting to a high-dimensional system, Koelle et al. (2019) developed a new class of low-dimensional within-host models whose structure flexibly allows for cellular coinfection [32]. Their model is based on the structure of epidemiological macroparasite models [37–39], where target cells are analogous to hosts and virions are analogous to macroparasites. In this model, target cells, either uninfected or (singly or multiply) infected, are represented by a single equation, dramatically reducing the dimensionality [32]. The model was fit to an influenza viral load data set and was found to perform better than standard influenza viral dynamics model. In addition, it was able to capture the peak viremia, which the standard viral dynamics model underestimates [32].

Here, we aim to characterize early HIV-1 infection dynamics by four models: the standard viral dynamics model, the Density-Dependent Cell Death model [19, 21], the MOI model [32] adapted to HIV-1 dynamics, and a novel model combining multiplicity of infection and density-dependent cell death. These models are fit to viral load data from 43 participants of the RV217 study of acute HIV-1 infection [20]. We find that the Density-Dependent Cell Death model performs best overall, but different models explain differing quantitative features of the dynamics more accurately.

## Materials and methods

### Ethics statement

This paper uses de-identified data obtained previously [20]. The study protocol was approved by the local ethics review boards and the Walter Reed Army Institute of Research. Written informed consent was obtained from all participants. The protocol was approved by:

- Walter Reed Army Institute of Research; Human Subjects Protection Board, WRAIR
- Makerere University Walter Reed Project, Kampala, Uganda; National AIDS Review Committee and the Uganda National Science and Technology Council
- Walter Reed Project, Kericho, Kenya; Kenya Medical Research Institute (OHRP IRB# IRB00001625) Scientific & Ethical Review Unit (SERU),
- Armed Forces Research Institute of Medical Sciences, Bangkok, Thailand; Royal Thai Army Medical Department Institutional Review Board

### Human HIV viral load data and exclusion criteria

**Fig 1:**
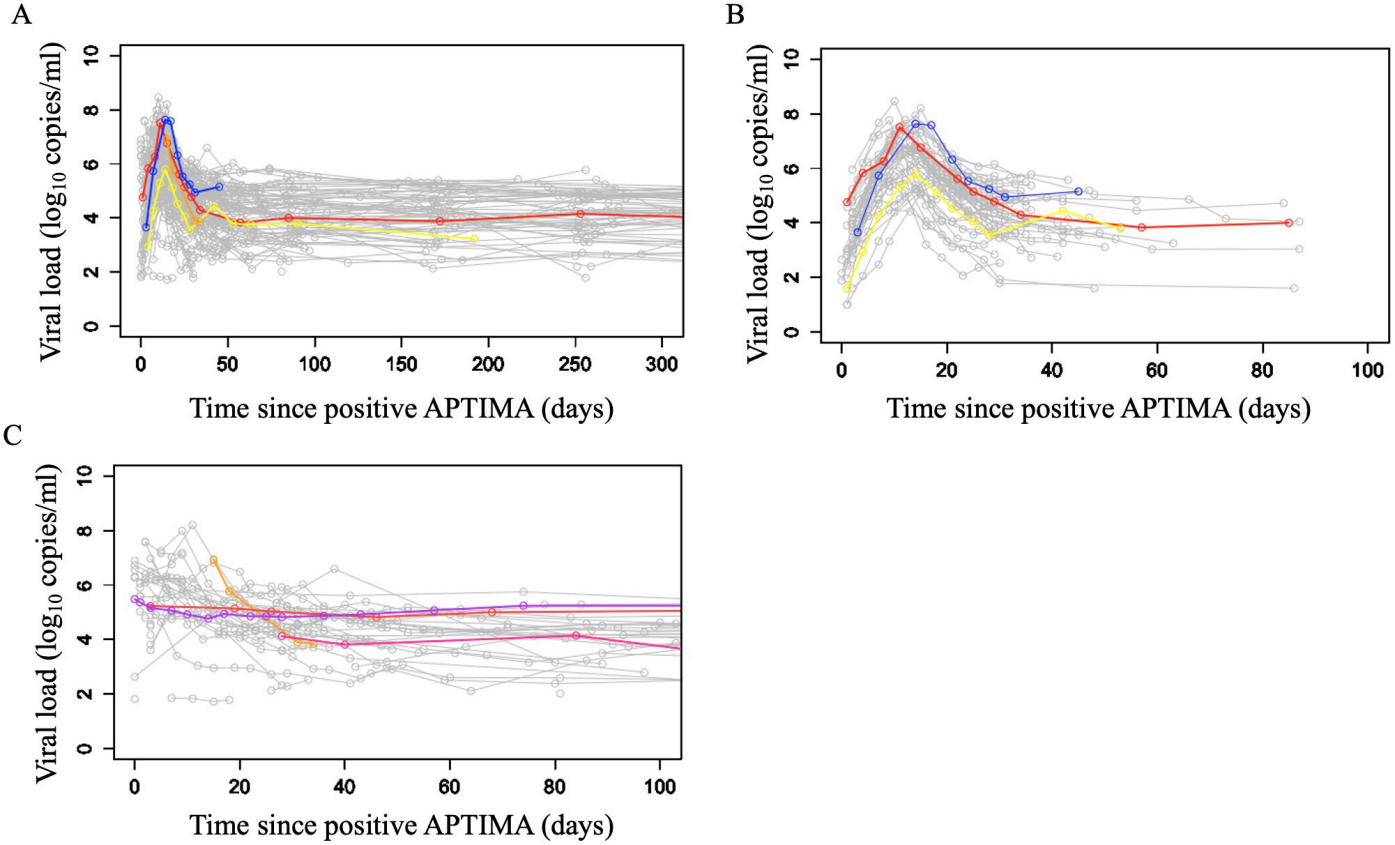
Viral load measurements from the RV217 study participants. Viral load measurements from A) all 77 participants from the RV217 study, B) the 43 study participants that were included in our analysis and C) from 34 study participants excluded from our analysis (grey lines) along with representative viral load trajectories (colored lines).

Viral load measurements were obtained from the RV217 study [20]. Participants were recruited in four locations (Uganda, Kenya, Tanzania and Thailand) between June 2009 and June 2015. Study participants underwent twice-weekly small-volume blood collection by fingerstick. Once tests for HIV-1 RNA were reactive, large-volume blood samples were collected twice weekly for 4 weeks. Participants with confirmed HIV-1 infection were enrolled in the long-term follow-up phase (Fig 1A). We include in our study all participants with at least one study visit before viral RNA detection, two viral load measurements before inferred peak viremia, and at least 8 viral load measurements in total (Fig 1B for study participants included and Fig 1C for study participants not included). Day 0 is defined as the day on which the first blood sample was reactive for HIV-1 RNA using Aptima HIV-1 RNA Qualitative Assay. We aim to characterize dynamics of acute infection and thus restrict viral load measurements to the second viral measurement after the nadir for each study participant.

### Data analysis

We aim to characterize viral quantitative measures for each study participant’s viral load curve. These measures include 1) the growth rate, 2) the decay rate, 3) the viral setpoint, 4) the peak viremia, 5) the timing of viral peak, and 6) a joint measurement of the the peak magnitude and timing.

**Fig 2:**
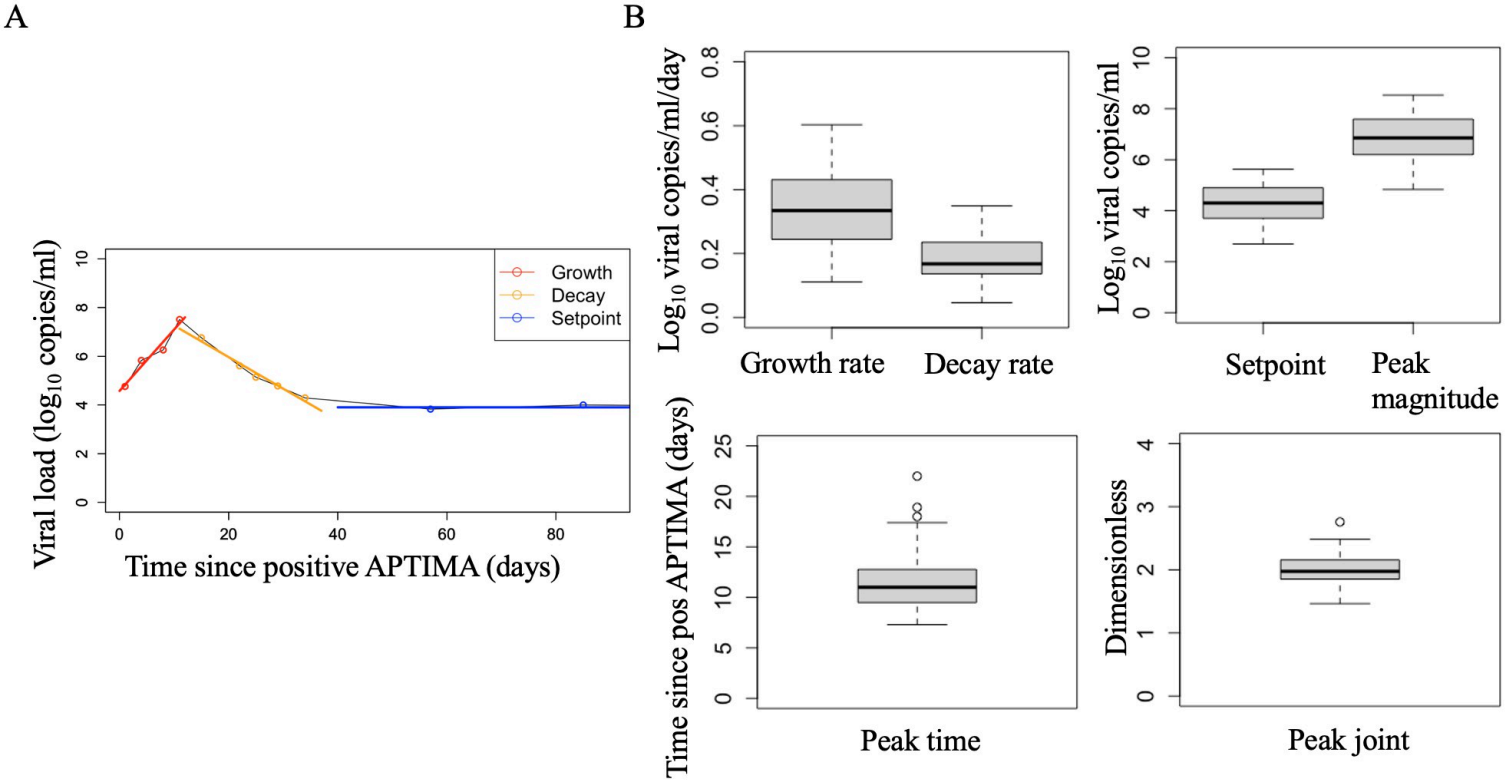
Viral quantitative measurements estimation and summary statistics. A) Example of how quantitative measures are estimated for each study participant. Points represent viral load measurements. Red points were identified as part of the growth phase and were used to estimate the growth rate via linear regression (red line). Similarly, the orange and blue points were identified as part of the viral decay phase and setpoint, respectively, and are graphed with regression lines. B) Summary statistics of quantitative measurements for the 43 study participants included in our analysis. Peak magnitude refers to the peak viremia predicted by the intersection of the growth and decay lines and peak time, the time when the viral peak occurs. We also derive a metric for the overal characterization the peak, called *peak joint* and defined as $\frac{\text{peak}\;\text{magnitude-mean}\;\text{peak}\;\text{magnitude}}{\text{mean}\;\text{peak}\;\text{magnitude}}+\frac{\text{peak}\;\text{timing-mean}\;\text{peak}\;\text{timing}}{\text{mean}\;\text{peak}\;\text{timing}}$.

We calculate the growth and decay rates and setpoint for each study participant in the following way: we categorize data points as belonging to the growth phase, decay phase or setpoint. We then fit linear regressions to the growth and decay data points. The slopes of these regressions are the growth and decay rates. For setpoint, we fit a horizontal line (Fig 2A for an illustration).

Peak magnitude is estimated as the maximum value between the observed maximum viral load and the intersection of the growth and decay lines. Peak timing is calculated as the time when the peak magnitude occurs. Finally, to account for both magnitude and timing of the peak we derive the a joint measurement that incorporates both measures:

$$\frac{\text{peak}\;\text{magnitude-mean}\;\text{peak}\;\text{magnitude}}{\text{mean}\;\text{peak}\;\text{magnitude}}+\frac{\text{peak}\;\text{timing-mean}\;\text{peak}\;\text{timing}}{\text{mean}\;\text{peak}\;\text{timing}}$$

Estimated quantitative measures are reported in S1 Table and a summary of those measurements in Fig 2B.

### The models

We fit each study participant’s viral loads individually. Fitting complex models to a few data points would be extremely difficult, but simple models remain appropriate. For this same reason, we do not test models that incorporate immune responses explicitly; such models would add a number of parameters and our dataset would not be sufficient for model fitting. Therefore, we focus on simple models.

**Fig 3:**
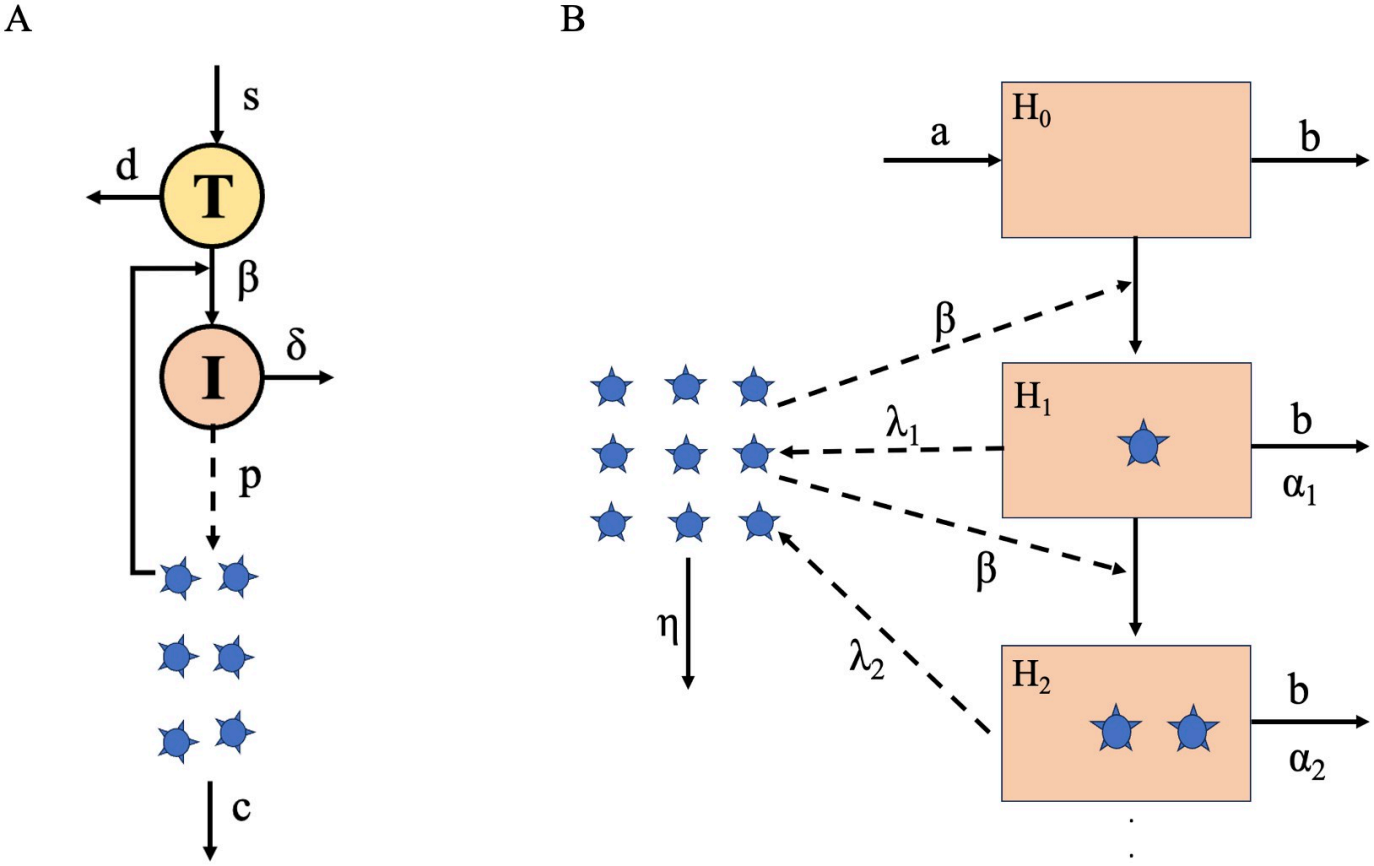
Standard viral dynamics model and Macroparasite model. A) Schematic of the standard model. Target cells, *T* are sourced at rate *s* and die at rate *d*. Virus *V* infects target cells at rate *β*. Infected cells, *I* die at rate *δ* and produce virions at rate *p*. Free virus gets cleared at rate *c*. B) Schematic of the Macroparasite model. Target cells, *H*_*i*_ are produced at a constant rate *a* and die at rate *b*. Target cells can be uninfected or infected with *i* virions. Here we demonstrate cells infected with up to 2 virions. Free virus infects target cells at rate *β*. Virus-induced mortality of infected cells is *α*_*i*_. Infected cells produce free virus at rate λ_*i*_. Virus gets cleared at rate *η*. In these schematics, *c* and *η* represent the same parameter, viral clearance. We choose to keep them separate to avoid confusion and remain consistent with previous publications.

To investigate infection dynamics among study participants, we use the following four models:

1. *Standard model*: The standard viral dynamics model of acute infection was developed to provide the first estimates of fundamental within-host parameters, such as the viral clearance rate and the infected cell lifespan [11] and to explore viral decay during treatment [12]. The standard model is a target cell-limited model, i.e. HIV infection is limited by the availability of target cells, consisting of three compartments– uninfected cells, infected cells and free virus. Target cells, *T*, are produced at rate *s*, die at rate *d* and become infected at rate *k*. Infected cells, *I*, die at a constant rate *δ* and produce HIV-1 virions at rate *p*. Free virus, *V*, is cleared from the blood at rate *c* (Fig 3A). This model can be summarized in the following system of differential equations:

$$\begin{array}{c} \frac{dT}{dt}=s-dT-kTV \end{array}$$ (1)

$$\begin{array}{c} \frac{dI}{dt}=kTV-\delta I \end{array}$$ (2)

$$\begin{array}{c} \frac{dV}{dt}=pI-cV \end{array}$$ (3)

This target-cell limited model has been shown to capture the dynamics of early infection, however it produces oscillatory dynamics and tends to underestimate the magnitude of peak viremia [10].

2. *Density-dependent death of infected cells*: An alternative model proposed by Holte et al. [19] was developed to explain the biphasic decay of HIV virus after treatment initiation. This model allows for nonlinear log decay of infected cell populations, thus capturing in a simple and rudimentary way aspects of immune responses. The model equations are identical to those of the standard viral dynamics model, with the exception of *δI*, which now becomes *δI*^*γ*^. *γ* represents density-dependence in the death of infected cells. Note that for *γ* = 1, this model reduces to the standard viral dynamics model.

3. *MOI model*: Both of the aforementioned models do not make specific assumptions about multiple cellular infections. In the case of HIV, however, there is evidence for cellular coinfection, both *in vivo* and *in vitro* [29, 40–42]. Previous models developed to allow for cellular coinfection are high-dimensional [35] and therefore complicated to ft to available data convincingly. To allow for cellular multiplicity of infection, without resorting to a high-dimensional system, Koelle et al. developed a within-host model that is a close analogy to the population level macroparasite models [32]. They used it to investigate within-host influenza dynamics and found an improved characterization of overall dynamics, as well as of peak viremia, motivating us to test this simple model as well. This is again a target cell-limited model and takes on the following general form:

$$\begin{array}{c} \frac{dH}{dt}=a-bH-H\sum_{i=0}^{\infty }\alpha_{i}p_{i} \end{array}$$ (4)

$$\begin{array}{c} \frac{dV}{dt}=H\sum_{i=0}^{\infty }\lambda_{i}p_{i}-\eta V-\beta HV \end{array}$$ (5)

$$\begin{array}{c} \frac{dP}{dt}=\beta HV-bH\sum_{i=0}^{\infty }ip_{i}-H\sum_{i=0}^{\infty }i\alpha_{i}p_{i} \end{array}$$ (6)

*H* represents the total number of target cells. This includes both uninfected and infected cells with a variable number of virions. Both unifected and infected cells are targets to further infection. Target cells are produced at a constant rate *a* and die at rate *b*. $\sum_{i=0}^{\infty }\alpha_{i}p_{i}$ is the infection-induced mortality of target cells. *α*_*i*_ is the death rate of target cells infected with *i* virions and *p*_*i*_ is the proportion of target cells that are infected with cellular MOI of *i*. The variable *V* is the concentration of free virus. $H\sum_{i=0}^{\infty }\lambda_{i}p_{i}$ represents the viral production, where λ_*i*_ is the viral production rate of cells infected with MOI *i*. Free virus is cleared at rate *η* and is lost due to entry into target cells at rate *β*. *P* represents the amount of internalized virus, across all target cells. *βHV* captures the increase in internalized virus due to cell entry of free virus. $bH\sum_{i=0}^{\infty }ip_{i}$ represents the loss of internalized virus due to the background mortality of target cells and $H\sum_{i=0}^{\infty }i\alpha_{i}p_{i}$ represents the loss of internalized virus due to the infection-induced mortality of target cells (Fig 3B). In the absence of data supporting a more complex formulation, we make the simple assumption that viral production rates and infection-induced mortality rates scale linearly with MOI (λ_*i*_ = *i* ⋅ λ, *α*_*i*_ = *i* ⋅ *α*), and that MOI follows a negative binomial distribution (Koelle et al. 2019), the system becomes the following:

$$\begin{array}{c} \frac{dH}{dt}=a-bH-\alpha P \end{array}$$ (7)

$$\begin{array}{c} \frac{dV}{dt}=\lambda P-\eta V-\beta HV \end{array}$$ (8)

$$\begin{array}{c} \frac{dP}{dt}=\beta HV-bP-\alpha P-\alpha \frac{1+k}{k}\frac{P^{2}}{H} \end{array}$$ (9)

where *k* is the dispersion parameter of the negative binomial distribution [32].

4. *Density-Dependent Death of infected cells & MOI*: By comparing a density-dependent infected cell death model to an MOI model, we will evaluate which mechanism dominates in early infection and therefore explains the data better. But both may be equally important. To account for both cellular coinfection and density-dependence of infected cell death, in a relatively simple model, we use the MOI model as a basis and incorporate density-dependence with rate *γ*. We assume that the sheer number of infected cells enhances the death rate. That number is reflected in the term $\sum_{i=0}^{\infty }\alpha_{i}p_{i}$ in the target cell equation. Similarly, internalized virus P is removed at a density-dependent way from the term $\sum_{i=0}^{\infty }i\alpha_{i}p_{i}$. These terms become ${\left(\sum_{i=0}^{\infty }\alpha_{i}p_{i}\right)}^{\gamma }$ and ${\left(\sum_{i=0}^{\infty }i\alpha_{i}p_{i}\right)}^{\gamma }$ respectively (for details please refer to the S1 Text).

Following the same simplifying assumptions as in the *MOI* model, the system of differential equations becomes the following:

$$\begin{array}{c} \frac{dH}{dt}=a-bH-\alpha^{\gamma }\frac{P^{\gamma }}{H^{\gamma -1}} \end{array}$$ (10)

$$\begin{array}{c} \frac{dV}{dt}=\lambda P-\eta V-\beta HV \end{array}$$ (11)

$$\begin{array}{c} \frac{dP}{dt}=\beta HV-bP-\alpha^{\gamma }\frac{P^{\gamma }}{H^{\gamma -1}}{\left[1+\frac{P}{H}\frac{\left(1+k\right)}{k}\right]}^{\gamma } \end{array}$$ (12)

We restrict ourselves to these four simple models. We avoid models incorporating explicit immune responses, as the inclusion of additional parameters would render it exceedingly difficult to accurately fit such models to our dataset, given the sparse nature of the data (8–13 measurements per study participant). We do anticipate that immunity plays a significant role in the later phase of acute infection dynamics, and in the viral setpoint [8, 10, 19].

### Model fitting

We aim to capture the heterogeneity in viral load dynamics among study participants. Our goal is not to select the best model for the population, but the best model for each study participant. Therefore, we choose to fit the viral load measurements of each study participant separately to each model. We fix the background target cell death rate *d* = 0.01 per day [10]. We assume that prior to infection, target cells are at equilibrium and therefore, we set *s* = *d* ⋅ *T*_0_, and fix the equilibrium target cell count *T*_0_ = 10^6^ cells/mL, assuming that 1 in 1000 cells are available as targets for infection [10, 43]. We fit the mass action infectivity (*β*), the infected cell death rate (*δ*), the viral production rate (*p*), the viral clearance rate (*c*), the dispersion parameter of the negative binomial distribution (*k*) for the MOI models, the intensity of density-dependence (*γ*), as well as *t*_0_, which is the start of exponential viral growth. We fit the parameters *θ* of each mathematical model by minimizing the error functional corresponding to the least squares estimate,

$$LSE\left(\theta \right)=\frac{\sum_{i=1}^{n}{\left[{\text{log}}_{10}\left(v_{i}\right)-{\text{log}}_{10}\left(V\left(t_{i},\theta \right)\right)\right]}^{2}}{n},$$

where *v*_*i*_ is the viral load measurement of the study participant at time *t*_*i*_, *V*(*t*_*i*_, *θ*) is the predicted viral load at time *t*_*i*_ by a specific model, parameterized by *θ* and *n* is the number of viral load measurements for that study participant. We use the *optim* package in *R* [44]. We report the mean, median and standard deviation of estimated parameter values of each model (Table 1), as well as the estimated values for each study participant (S2–S5 Tables).

**Table 1:** Parameter values: Mean values and standard deviation for parameters of each model.

	log_10_ viral production (virions/cell day)	infected cell death rate (day^−1^)	viral clearance rate (day^−1^)	mass action infectivity (mL/ virion day)	*t*_0_ (days)	*γ*	Dispersion parameter
Standard	2.07(0.71)	0.81(1.22)	9.98(6.94)	6.77 × 10^−7^(1.72 × 10^−6^)	−8.41(6.77)		
Density-Dependent Cell Death MOI	3.04(0.73)	2.08(1.39)	13.1(9.37)	1.42 × 10^−7^(3.5 × 10^−7^)	−6.13(5.12)	1.04(1.03)	
	2.29(0.81)	1.04(0.99)	11(10.02)	6.8 × 10^−7^(1.9 × 10^−6^)	−11.3(8.1)		3.01(3.89)
Density-Dependent Cell Death & MOI	2.7(1.14)	2.3(3.13)	10.74(3)	1.69 × 10^−6^(6.8 × 10^−6^)	−9.03(4.61)	1.02(1.02)	9.75(16.9)

### Model comparison

To determine which model explains the data set best for each participant, we use the Akaike Information Criterion (AIC), the Bayesian Information Criterion (BIC) and the corrected Akaike Information Criterion (AICc). BIC penalizes more heavily for more parameters [45], and AICc corrects for small sample sizes [46].

In addition to assessing the overall performance of the model, we are interested in evaluating their ability to predict certain quantitative measures, namely: 1) viral growth rate, 2) viral peak magnitude, 3) viral peak time, 4) a joint peak measurement that combines both magnitude and time, 5) viral decay rate and 6) setpoint. Growth and decay rates are calculated as the tangents of the growth part and decay part of the fitted viral load curve (Fig 2A). Setpoint is the steady-state viremia. Peak magnitude is calculated as the maximum viral load and peak timing is the time when the maximum viral load occurs. The joint measurement for the peak is sum of the difference between the magnitude and the timing weighted by the respective means, as described above,

$$\frac{\text{peak}\;\text{magnitude-mean}\;\text{peak}\;\text{magnitude}}{\text{mean}\;\text{peak}\;\text{magnitude}}+\frac{\text{peak}\;\text{timing-mean}\;\text{peak}\;\text{timing}}{\text{mean}\;\text{peak}\;\text{timing}}.$$

The model that explains best a quantitative measure, is one where the model-predicted value is closest to the data-observed value across all study participants. To assess that, we calculate a linear regression between the data-derived and the model-predicted values. Note that here, our objectives are different, we assume we know the quantity of interest, measured in the data (e.g. viral peak) and want to know how well the model derived quantities match those across all individuals. This is achieved (theoretically) when the intercept is zero and the slope 1, i.e. we have a perfect match between data-derived and model-derived quantities. In this case the estimated regression is *y* = *x*, so overall, the model-derived peak is closest to the data-derived peak. Therefore, the model that explains a quantitative measure best is the one for which the regression coefficient is closest to 1 and the intercept is closest to 0, meaning that the model-predicted value is closest to the data-derived value. To evaluate the regression lines, we use *R*^2^, which helps in assessing how well the independent variable (here we assume is the model-derived quantitative measure) explain the variability observed in the dependent variable (i.e. data-derived quantitative measure). A higher *R*^2^ value indicates a better fit, meaning that a larger proportion of the variance in the data-derived measure is explained by the model-derived measure.

## Results

**Fig 4:**
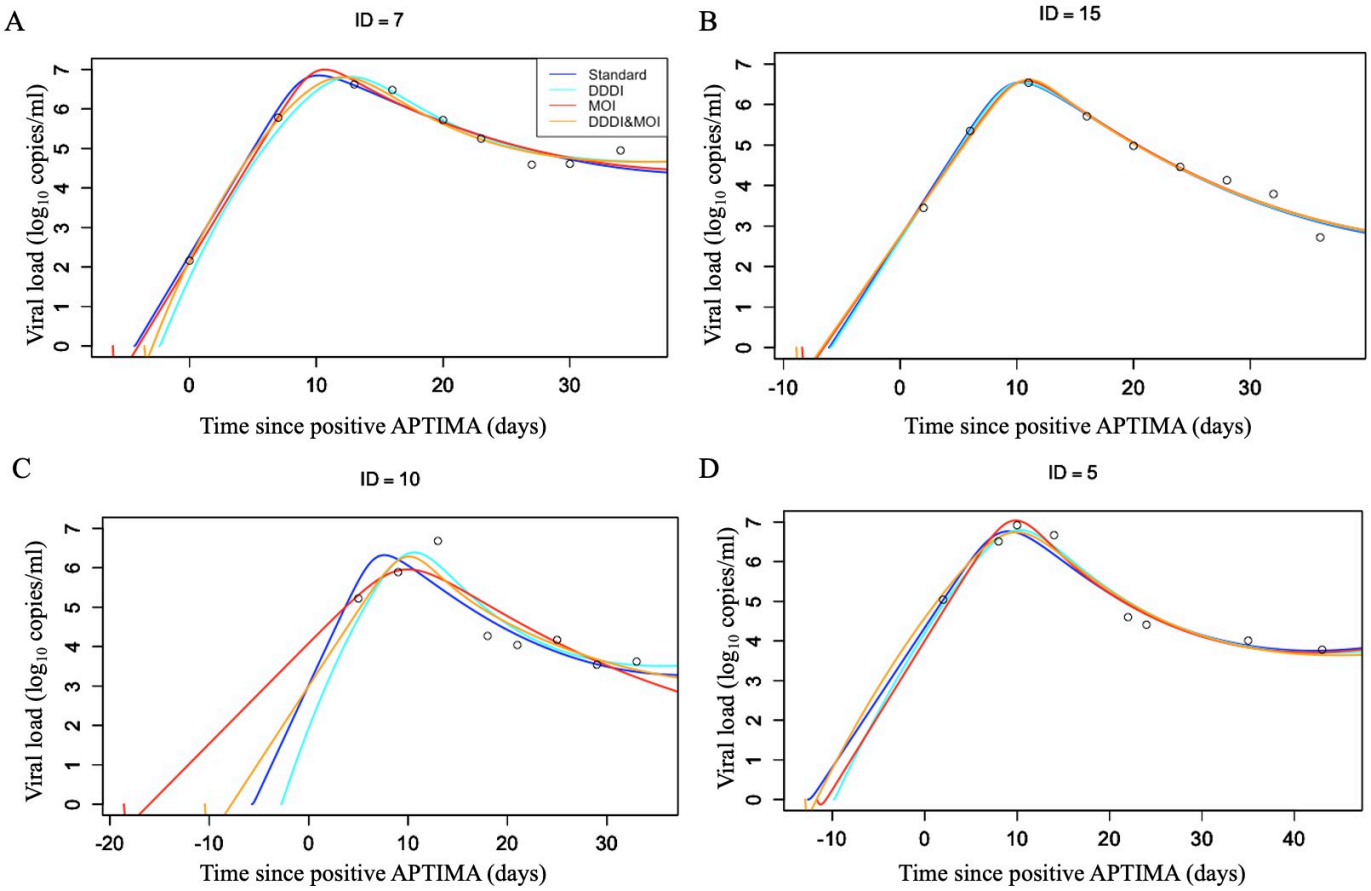
Model fits to study participant viral loads. Viral load measurements for four representative study participant (black points) and fitted curves for the Standard model (blue line), Density dependent death of infected cells(DDDI) model (cyan line), MOI model (red line) and Density dependent cell death model & MOI model (orange line). There are study participants where all models are comparably good at capturing the viral load data (A and B), whereas others (C) where they do not. We also show a case where the MOI model is better able to capture the viral peak (D).

**Fig 5:**
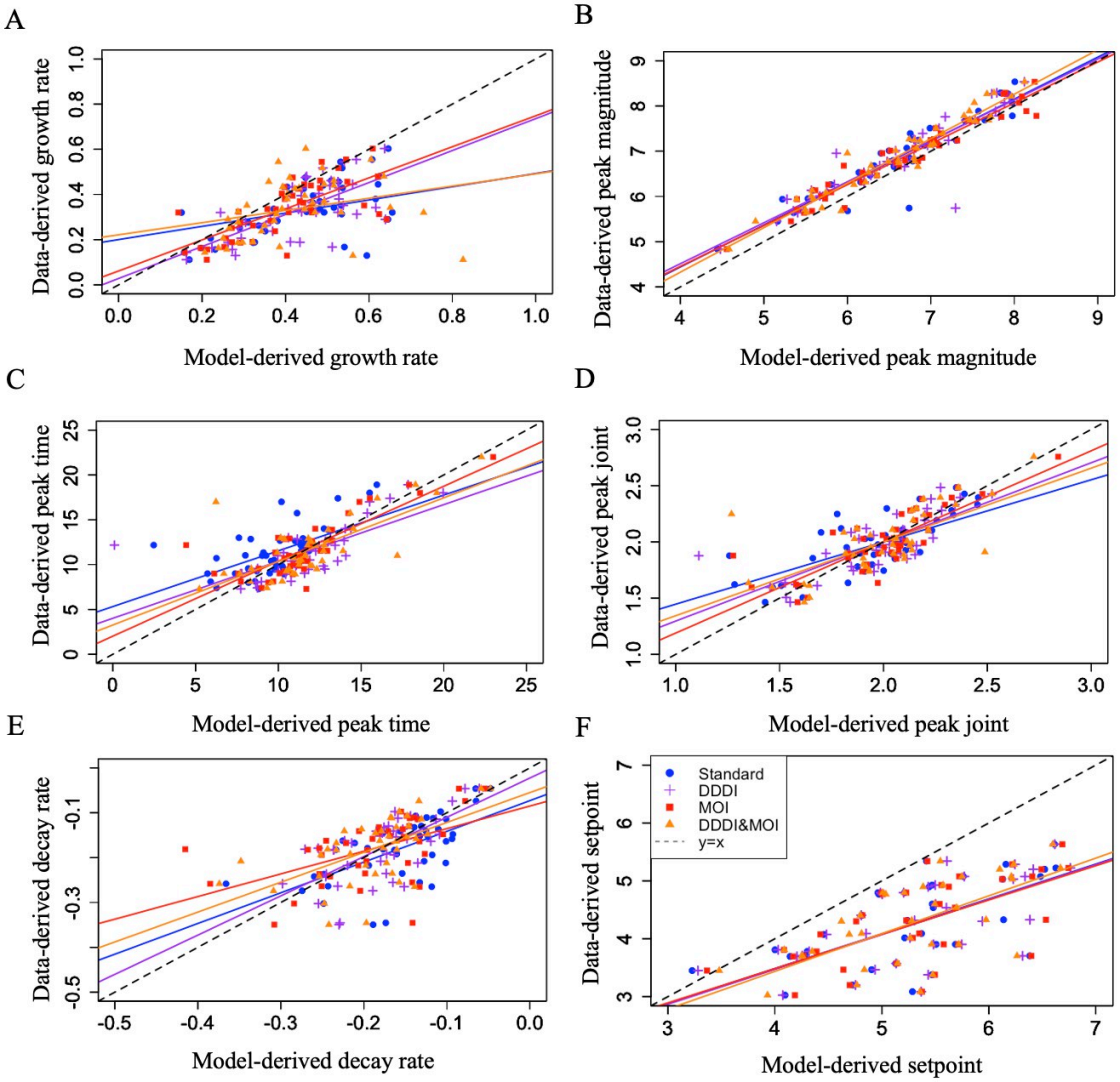
Estimated burst size distributions. Violin plots of the estimated viral burst sizes (on the $log_{10}$ scale) along with the median estimates (black dashed lines) for the all models. For the Standard and Macroparasite models, burst size remains constant throughout the course of acute infection (blue violins). For the models incorporating density-dependence in the death of infected cells DDDI, burst size changes over time and here we report the minimum (pink half-violin) and maximum estimates (soft pink half-violin). Details of the burst size calculations are included in the S2 Text.

*The Density-Dependent Cell Death model outperforms by AIC, BIC*: We report the fitted parameter values for each study participant and each model (S2–S5 Tables) and the fitted curves to the viral load measurements of each study participants (Figs 4, and S1–S43). We observe that all models perform comparably well. Table 1 contains the mean values of the estimated parameters for each model. The mean values of the infected cell death rate and viral clearance rate for all models appears to be higher compared to reported values for acute infection [10]. Mean values for viral production rate and mass action infectivity are comparable to reported values [10, 26]. Finally, model-predicted burst sizes are higher for the Density-Dependent Cell Death model (Fig 5), even though the Macroparasite & Density-Dependent Cell Death model allows for a longer tail of higher burst sizes (for burst size estimation, please refer to S2 Text). These estimates are still underestimations of the burst size, but more consistent with the values provided for Simian Immunodeficiency Virus (SIV) [16]. We also note that the Density-Dependent Cell Death & MOI model is more difficult to fit.

We first compare the performance of each model by computing the Akaike Information Criterion (AIC), the Bayesian Infromation Criterion (BIC) and the corrected Akaike Information Criterion (AICc). Table 2 reports the number of study participants for which a particular model is selected by each estimator. The Density-Dependent Cell Death model is selected by AIC and BIC for the majority of study participants (24 out of 43 study participants). AICc selects overwhelmingly for the Standard model (39 out of 43), suggesting that the standard model performs better for a small number of data points. Using AIC, BIC and AICc, we are interested in determining if other models perform comparably well to the best model for each study participant. In Table 2, we also report for how many study participants a model fit yields the lowest or comparable to the lowest AIC, BIC and AICc (i.e. a difference of < 4 [47]). We find that the Density-Dependent Cell Death model still outperforms by AIC and BIC, whereas the Standard model outperforms by AICc. However, for a considerable number of study participants, the standard model provides comparable fits to the density-dependent model (using AIC—29 participants, or BIC—32 participants). The MOI and Density-Dependent & MOI models consistently underperform by AIC, BIC and AICc (Tables 2, S6 and S7).

**Table 2:** AIC, BIC and AICc. Each row of the table represents for how many study participants the AIC/BIC/AICc is the lowest or comparable to the lowest.

	AIC	AIC not different	BIC	BIC not different	AICc	AICc not different
Standard	17	29	17	32	40	40
Density-Dependent Cell Death MOI	24	36	24	35	1	5
	0	4	0	4	0	0
Density-Dependent Cell Death & MOI	2	7	2	6	2	2

**Fig 6:**
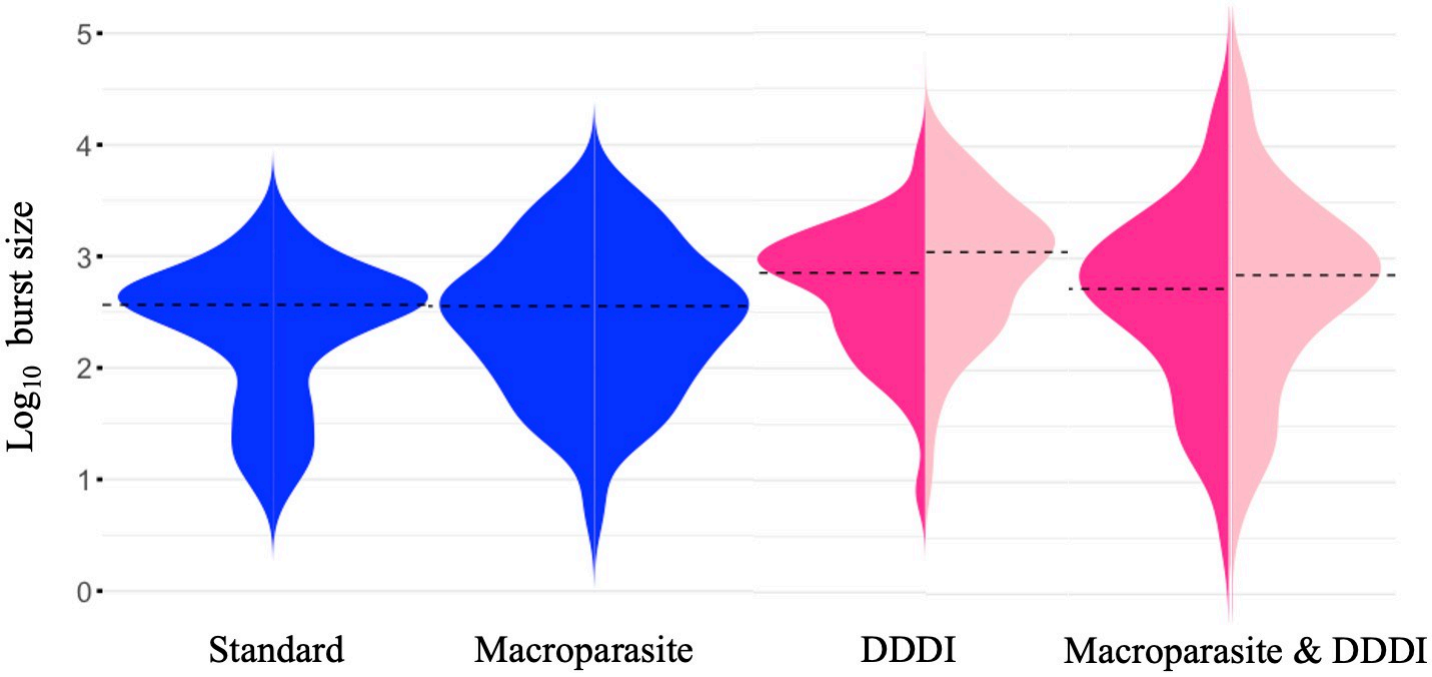
Regressions for all viral quantitative measures. Data-derived and model-derived quantitative measurements (solid points) for each study participant and model, along with the fitted linear regression lines (solid lines) for the growth rate (A), peak magnitude (B), decay rate (C), peak time (D), joint peak measurement(E) and setpoint (F). The model that explains best a quantitative measurement is the one where the model-derived value is closest to the data-derived value, i.e the fitted linear regression is closest to the y = x line (dashed line). Statistical summaries of the fitted regressions can be found in Table 3.

The AIC, BIC and AICc provide an estimate for the goodness of fit for each model, taking into account model complexity. We are interested in determining how well these models can predict specific viral quantitative measurements namely, the growth rate, the peak, the peak timing, the decay rate and the setpoint. To determine how well each model explains a quantitative measurement, we fit a linear regression between the data-derived and the model-derived values of that quantitative measurement. The model that describes the quantitative measurement the best is the one for which the regression coefficient is closest to 1, the intercept is closest to 0 and has the highest *R*^2^. Table 3 contains the regression fits for each model. Fig 6 shows the data-derived and model-derived quantitative measures and the respective regression lines.

**Table 3:** Comparison of quantitative measurements: We report the coefficient, intercept (related p-values in parentheses) and *R*^2^ of the linear regression fit to the data-derived and model-derived growth rate for each model tested. Regression lines are depicted in Fig 3.

Measurement	Model	Coefficient	Intercept	Adjusted *R*^2^
Growth Rate	Standard	0.29 (3.57 × 10^−8^)	0.2 (0.0003)	0.14
	Density-Dependent Cell Death MOI	0.71 (0.014)	0.03 (0.59)	0.47
		0.69 (0.006)	0.06 (0.18)	0.49
	Density-Dependent Cell Death & MOI	0.27 (5 × 10^−6^)	0.22 (0.0008)	0.06
Peak Magnitude	Standard	0.93 (0.15)	0.73 (0.03)	0.89
	Density-Dependent Cell Death MOI	0.91 (0.12)	0.9 (0.03)	0.85
		0.9 (0.01)	0.84 (0.0013)	0.94
	Density-Dependent Cell Death & MOI	0.98 (0.69)	0.39 (0.16)	0.93
Peak Time	Standard	0.62 (9.44 × 10^−5^)	5.32 (1.65 × 10^−6^)	0.54
	Density-Dependent Cell Death MOI	0.63 (4.27 × 10^−5^)	4.01 (0.0003)	0.6
		0.84 (0.09)	2.1 (0.08)	0.65
	Density-Dependent Cell Death & MOI	0.71 (0.009)	3.3 (0.02)	0.51
Joint Peak Measurement	Standard	0.55 (7.33 × 10^−8^)	0.89 (1.09 × 10^−7^)	0.6
	Density-Dependent Cell Death MOI	0.7 (0.0003)	0.6 (0.0003)	0.68
		0.81 (0.04)	0.38 (0.04)	0.66
	Density-Dependent Cell Death & MOI	0.66 (0.003)	0.69 (0.003)	0.47
Decay Rate	Standard	0.68 (0.03)	−0.07 (0.003)	0.38
	Density-Dependent Cell Death MOI	0.88 (0.45)	−0.02 (0.46)	0.41
		0.5 (0.0005)	−0.08 (0.0003)	0.26
	Density-Dependent Cell Death & MOI	0.67 (0.027)	−0.05 (0.7)	0.33
Setpoint	Standard	0.6 (0.0023)	1.07 (0.11)	0.4
	Density-Dependent Cell Death MOI	0.6 (0.001)	1.6 (0.09)	0.43
		0.59 (0.001)	1.11 (0.08)	0.41
	Density-Dependent Cell Death & MOI	0.65 (0.008)	0.83 (0.21)	0.43

*All models overestimate the growth rate*: We find that the growth rate is best explained by the Density-Dependent Infected Cell Death model. Notably, the Standard model and the Density-Dependent Infected Cell Death & MOI model provide growth rate estimates that deviate significantly from the growth rate determined from data (Tables 3 and S8). All models tend to predict fast growth, much faster than the data suggests. We hypothesize that this may attributable to a central assumption of all models: mass action infectivity of target cells. The models we explore do not incorporate the effects of spatial structure in the viral population that may be important especially in early dynamics before the infection becomes systemic. For HIV there is evidence for a spatial structure coming from genetic compartmentalization of the virus [35, 48, 49].

*Models including cellular coinfection more accurately predict peak viral loads*: All models account for peak magnitude (Tables 3 and S9), with the Density-Dependent Cell Death & MOI model emerging as the most effective choice. When it comes to pinpointing the timing of the peak, the MOI model outperforms the others (Tables 3 and S10). Additionally, our comprehensive measure of the peak, which integrates both its magnitude and timing, is more aptly explained by the MOI model, as shown in Tables 3 and S11. These findings strongly imply that burst-size heterogeneity plays a pivotal role in elucidating the dynamics of the viral peak. Our hypothesis revolves around the idea that cellular coinfection, more likely in regimes of high viremia, results in increased viral output as compared to single-virion cell infection. This ability to infect a cell multiple times diminishes the constraints imposed by target cell limitation, ultimately leading to higher peak viremia.

*Models with density-dependent death better predict post-peak decay*: the decay rate is better explained by the Density-Dependent Cell Death model (Tables 3 and S12), suggesting that immune responses are increasingly important in explaining viral dynamics. It should be noted that the Standard model is nested within the Density-Dependent Cell Death model. The presence of the *γ* parameter in the Density-Dependent Cell Death model, allows for more flexibility to capture both fast and slow decays.

*All models overestimate the setpoint*: Even though the setpoint is better explained by the Density-Dependent Cell Death & MOI model (Tables 3 and S13), it should be noted that none of the models is particularly successful in explaining it. All models overestimate viral setpoint, which suggests that there are additional mechanisms at play not accounted for in our modeling.

## Discussion

In this study, we tested four mathematical models, each incorporating different hypotheses, to investigate acute infection dynamics in 43 people tested very early after HIV exposure. It should be noted that this is a particularly rich dataset, since for the study participants included in this analysis, there are at least 8 viral load measurements some of which were detected before peak viremia. We find that the Density-Dependent Cell Death model is relatively better at fitting these viral load measurements AIC/BIC. However, different models are better as describing different quantitative measurements of acute HIV infection, namely the growth and decay rates, peak viremia and setpoint. We fit the models to each study participant separately, because we interested in describing the heterogeneity in viral dynamics among study participants. This also informs our model choices: we focus on simple models, as the small number of viral measurements per study participant would not allow the fitting of more complicated models with a higher number of parameters. The Density-Dependent Cell Death model provides the best fit by AIC and BIC, which is consistent with the results of Reeves et al. (2021) [21]. Reeves et al. fitted several compartmental models to the RV217 dataset in a nonlinear mixed-effects framework and determined that the model explaining the data best is the Density-Dependent Cell Death one [21]. For their fitting, they fix the viral clearance rate to 23 *day*^−1^. We estimated a mean value of 13.1 *day*^−1^, which is lower compared to their fixed value. We also find mass action infectivity to be approximately three orders of magnitude lower compared to their estimates. In contrast, we find the infected cell death rate to be an order of magnitude higher compared to Reeves et al’s estimates. Surprisingly, the simple target cell-limited model also provides reasonable fits, and with fewer parameters is often the best model by AICc.

Peak viremia, both its timing and magnitude, is best explained by models that incorporate cellular coinfection. This is analogous to Koelle et al. [32]. In their study, they validated their MOI model using within-host data from ponies experimentally infected with influenza virus A subtype H3N8. In addition to finding by model selection that the MOI model performs better than the standard model, it also captures the peak more effectively than the standard model, which tends to underestimate it, both for influenza and HIV. It appears that allowing for multiple cellular infections in the modeling framework and permitting increased viral production rates for multiply infected cells, increases plasma viremia closer to observed peak levels. Regarding the magnitude of the peak, we find that the combination of MOI and Density-Dependent Cell Death is most successful at capturing it. This finding suggests that immune responses start to appear and play a role in viral dynamics. The decay rate is also best explained by the Density-Dependent Cell Death model, indicating that the effect of immune responses is exacerbated. Our results support that immune responses contribute to the initial decline in viral load, but do not exclude the effect of target cell limitation, as suggested by Philips (1996) [9, 10]. Indeed there have been observations that suggest that the immune systems controls viremia during primary infection: there is an negative association between an increase in CTL frequency and a decline in viremia [8]. In addition, there has been documented an inverse correlation between the setpoint viral load and the number of effector CTLs [10, 50].

We found that the model that describes the setpoint best is the Density-dependent cell death model & MOI, indicating that both mechanisms incorporated may be important in explaining viral dynamics. Nevertheless, no model, including the Density-dependent cell death model & MOI model one is particularly successful in explaining the setpoint, as is demonstrated by both the regression coefficient and the adjusted *R*^2^. This suggests that these models are not enough to explain setpoint. Adaptive immune responses are already present and may play an important role in controlling the virus. Instead a model that explicitly incorporates adaptive immune responses is expected to explain setpoint dynamics more effectively.

Cellular coinfection in people with HIV has been experimentally demonstrated [29, 40–42]. The coinfection of a cell with multiple virions can lead to the formation of new recombinant viruses [29], which in turn can affect host adaptation [30, 35], as well as the emergence and spread of drug resistance [31, 51–53]. Indeed higher viral loads are associated with higher recombinations rates, suggesting that recombination varies dynamically over time [54]. The MOI model developed by Koelle et al. [32] is not the first attempt to model cellular coinfection. A class of ODE models was developed by Wodarz & Levy [35], Guo et al. [36] and Dixit & Perelson [18, 33]. These models are high-dimensional and, to our knowledge, have mainly been used to derive qualitative conclusions about viral dynamics. In the MOI model, we make certain assumptions, namely that both the viral production rate, λ_*i*_, and the infected cell death rate, *α*_*i*_, scale linearly with MOI, *i* (λ_*i*_ = λ ⋅ *i*, *α*_*i*_ = *α* ⋅ *i*). This assumption is consistent with Koelle et al. (2019) [32]. Regardless, there are different burst size distributions that could be explored by the model. Indeed, an increased burst size can alter basic infection dynamics: under this scenario, virus population does not follow straight exponential growth, but the rate of exponential growth can increase over time, as viremia increases [34], with effects on host adaptation. Despite these theoretical predictions, there are no empirical experiments on HIV that quantify these relationships.

Even though all models are able to describe the viral load dynamics of acute HIV infection, no single model can fully capture those dynamics, since different ones describe different quantitative measures best. We focused on simple models. There are, however, more complex models that could be tested that incorporate a variety of mechanisms; for example: models that incorporate the dynamics of the latent reservoir [55–63]; models that incorporate immune responses [28, 64, 65]; models that include both the latent reservoir and immune responses [26, 66]; models that incorporate an eclipse phase between cell infection and viral production [67–70]; models separating target cells into long- and short-lived [71]; models that include other types of target cells such as macrophages [72]; and models of evolution, such as viral competition [73].

AIC, BIC and AICc are valuable in evaluating the overall goodness of fit and to compare models of different number of parameters. However, as our results suggest, these estimators do not provide a complete picture of a model’s performance. Even though a particular model best explains overall dynamics, different models explain different specific aspects of the dynamics. For example, we find that overall, HIV-1 acute infection dynamics are best described by a Density-Dependent Infected Cell Death model [19], yet peak viremia is best explained by the MOI model [32]. This suggests that if we are interested in the viral peak, instead of the Density Dependent Infected Cell Death, we should use the MOI model. In spite of these, we find that all models provide insight into the viral kinetics and within-host dynamics of acute HIV infection.

All analysis code, along with a simulated test dataset are available at https://github.com/elliemainou/acute-infection.

## Supporting information

S1 Text Derivation of simplified terms in the Density-Dependent Cell Death & MOI model. (DOCX)

S2 Text Burst size calculation. (DOCX)

S1 Table Growth, decay, peak and setpoint linear model parameter estimation and Standard-model derived R0 for each study participant. (DOCX)

S2 Table Estimates for the Standard model. Parameter value estimates for the Standard model, along with the number of data points used to fit the model, the negative log likelihood (nll), AIC, BIC and AICc. We also report mean, median and interquartile range (IQR) for the reader reference.
(DOCX)

S3 Table Estimates for the Density-Dependent Cell Death model. Parameter value estimates for the Density-Dependent Cell Death model, along with the negative log likelihood (nll), AIC, BIC and AICc. We also report mean, median and interquartile range (IQR) for the reader reference.
(DOCX)

S4 Table Estimates for the MOI model. Parameter value estimates for the MOI model, along the negative log likelihood (nll), AIC, BIC and AICc.
(DOCX)

S5 Table Estimates for the MOI model. Parameter value estimates for the Density-Dependent Cell Death & MOI model, along the negative log likelihood (nll), AIC, BIC and AICc.
(DOCX)

S6 Table Selected models. Summary of which model is selected by AIC, BIC and AICc for each study participant.
(DOCX)

S7 Table Negative log-likelihood summary. Sum of negative log likelihood (NLL), AIC, BIC, AICc across all study participants (n = 43).
(DOCX)

S8 Table Growth rate estimations. Data-derived growth rate and model-derived growth rates for each study participants, along with the squared difference for the data-and-model derived rates for each model.
(DOCX)

S9 Table Decay rate estimations. Data-derived decay rate and model-derived decay rates for each study participants, along with the squared difference for the data-and-model derived rates for each model.
(DOCX)

S10 Table Setpoint estimations. Data-derived setpoint and model-derived setpoint for each study participants, along with the squared difference for the data-and-model derived setpoint for each model.
(DOCX)

S11 Table Peak magnitude estimations. Data-derived peak magnitude and model-derived peak magnitude for each study participants, along with the squared difference for the data-and-model derived peak magnitude for each model.
(DOCX)

S12 Table Peak time estimations. Data-derived peak time and model-derived peak time for each study participants, along with the squared difference for the data-and-model derived peak time for each model.
(DOCX)

S13 Table Joint peak estimations. Data-derived joint peak measurement and model-derived joint peak measurement for each study participants, along with the squared difference for the data-and-model derived joint peak measurement for each model.
(DOCX)

S1 Fig All model fits to study participant 1. We provide the fitted curves of the four models (solid lines) to the viral load measurements (points). The black line represents the Standard model, the green line shows the Density-dependent cell death model, the pink line shows the MOI model and orange line represents the Density-dependent cell death & MOI model. Time 0 is the day of the first positive result in the Aptima HIV-1 RNA Qualitative Assay and a quantitative viral load measurement was not taken at that day.
(PDF)

S2 Fig All model fits to study participant 2. We provide the fitted curves of the four models (solid lines) to the viral load measurements (points). The black line represents the Standard model, the green line shows the Density-dependent cell death model, the pink line shows the MOI model and orange line represents the Density-dependent cell death & MOI model. Time 0 is the day of the first positive result in the Aptima HIV-1 RNA Qualitative Assay and a quantitative viral load measurement was not taken at that day.
(PDF)

S3 Fig All model fits to study participant 4. We provide the fitted curves of the four models (solid lines) to the viral load measurements (points). The black line represents the Standard model, the green line shows the Density-dependent cell death model, the pink line shows the MOI model and orange line represents the Density-dependent cell death & MOI model. Time 0 is the day of the first positive result in the Aptima HIV-1 RNA Qualitative Assay and a quantitative viral load measurement was not taken at that day.
(PDF)

S4 Fig All model fits to study participant 5. We provide the fitted curves of the four models (solid lines) to the viral load measurements (points). The black line represents the Standard model, the green line shows the Density-dependent cell death model, the pink line shows the MOI model and orange line represents the Density-dependent cell death & MOI model. Time 0 is the day of the first positive result in the Aptima HIV-1 RNA Qualitative Assay and a quantitative viral load measurement was not taken at that day.
(PDF)

S5 Fig All model fits to study participant 6. We provide the fitted curves of the four models (solid lines) to the viral load measurements (points). The black line represents the Standard model, the green line shows the Density-dependent cell death model, the pink line shows the MOI model and orange line represents the Density-dependent cell death & MOI model. Time 0 is the day of the first positive result in the Aptima HIV-1 RNA Qualitative Assay and a quantitative viral load measurement was not taken at that day.
(PDF)

S6 Fig All model fits to study participant 7. We provide the fitted curves of the four models (solid lines) to the viral load measurements (points). The black line represents the Standard model, the green line shows the Density-dependent cell death model, the pink line shows the MOI model and orange line represents the Density-dependent cell death & MOI model. Time 0 is the day of the first positive result in the Aptima HIV-1 RNA Qualitative Assay and a quantitative viral load measurement was not taken at that day.
(PDF)

S7 Fig All model fits to study participant 8. We provide the fitted curves of the four models (solid lines) to the viral load measurements (points). The black line represents the Standard model, the green line shows the Density-dependent cell death model, the pink line shows the MOI model and orange line represents the Density-dependent cell death & MOI model. Time 0 is the day of the first positive result in the Aptima HIV-1 RNA Qualitative Assay and a quantitative viral load measurement was not taken at that day.
(PDF)

S8 Fig All model fits to study participant 11. We provide the fitted curves of the four models (solid lines) to the viral load measurements (points). The black line represents the Standard model, the green line shows the Density-dependent cell death model, the pink line shows the MOI model and orange line represents the Density-dependent cell death & MOI model. Time 0 is the day of the first positive result in the Aptima HIV-1 RNA Qualitative Assay and a quantitative viral load measurement was not taken at that day.
(PDF)

S9 Fig All model fits to study participant 12. We provide the fitted curves of the four models (solid lines) to the viral load measurements (points). The black line represents the Standard model, the green line shows the Density-dependent cell death model, the pink line shows the MOI model and orange line represents the Density-dependent cell death & MOI model. Time 0 is the day of the first positive result in the Aptima HIV-1 RNA Qualitative Assay and a quantitative viral load measurement was not taken at that day.
(PDF)

S10 Fig All model fits to study participant 20. We provide the fitted curves of the four models (solid lines) to the viral load measurements (points). The black line represents the Standard model, the green line shows the Density-dependent cell death model, the pink line shows the MOI model and orange line represents the Density-dependent cell death & MOI model. Time 0 is the day of the first positive result in the Aptima HIV-1 RNA Qualitative Assay and a quantitative viral load measurement was not taken at that day.
(PDF)

S11 Fig All model fits to study participant 21. We provide the fitted curves of the four models (solid lines) to the viral load measurements (points). The black line represents the Standard model, the green line shows the Density-dependent cell death model, the pink line shows the MOI model and orange line represents the Density-dependent cell death & MOI model. Time 0 is the day of the first positive result in the Aptima HIV-1 RNA Qualitative Assay and a quantitative viral load measurement was not taken at that day.
(PDF)

S12 Fig All model fits to study participant 22. We provide the fitted curves of the four models (solid lines) to the viral load measurements (points). The black line represents the Standard model, the green line shows the Density-dependent cell death model, the pink line shows the MOI model and orange line represents the Density-dependent cell death & MOI model. Time 0 is the day of the first positive result in the Aptima HIV-1 RNA Qualitative Assay and a quantitative viral load measurement was not taken at that day.
(PDF)

S13 Fig All model fits to study participant 23. We provide the fitted curves of the four models (solid lines) to the viral load measurements (points). The black line represents the Standard model, the green line shows the Density-dependent cell death model, the pink line shows the MOI model and orange line represents the Density-dependent cell death & MOI model. Time 0 is the day of the first positive result in the Aptima HIV-1 RNA Qualitative Assay and a quantitative viral load measurement was not taken at that day.
(PDF)

S14 Fig All model fits to study participant 24. We provide the fitted curves of the four models (solid lines) to the viral load measurements (points). The black line represents the Standard model, the green line shows the Density-dependent cell death model, the pink line shows the MOI model and orange line represents the Density-dependent cell death & MOI model. Time 0 is the day of the first positive result in the Aptima HIV-1 RNA Qualitative Assay and a quantitative viral load measurement was not taken at that day.
(PDF)

S15 Fig All model fits to study participant 25. We provide the fitted curves of the four models (solid lines) to the viral load measurements (points). The black line represents the Standard model, the green line shows the Density-dependent cell death model, the pink line shows the MOI model and orange line represents the Density-dependent cell death & MOI model. Time 0 is the day of the first positive result in the Aptima HIV-1 RNA Qualitative Assay and a quantitative viral load measurement was not taken at that day.
(PDF)

S16 Fig All model fits to study participant 26. We provide the fitted curves of the four models (solid lines) to the viral load measurements (points). The black line represents the Standard model, the green line shows the Density-dependent cell death model, the pink line shows the MOI model and orange line represents the Density-dependent cell death & MOI model. Time 0 is the day of the first positive result in the Aptima HIV-1 RNA Qualitative Assay and a quantitative viral load measurement was not taken at that day.
(PDF)

S17 Fig All model fits to study participant 27. We provide the fitted curves of the four models (solid lines) to the viral load measurements (points). The black line represents the Standard model, the green line shows the Density-dependent cell death model, the pink line shows the MOI model and orange line represents the Density-dependent cell death & MOI model. Time 0 is the day of the first positive result in the Aptima HIV-1 RNA Qualitative Assay and a quantitative viral load measurement was not taken at that day.
(PDF)

S18 Fig All model fits to study participant 28. We provide the fitted curves of the four models (solid lines) to the viral load measurements (points). The black line represents the Standard model, the green line shows the Density-dependent cell death model, the pink line shows the MOI model and orange line represents the Density-dependent cell death & MOI model. Time 0 is the day of the first positive result in the Aptima HIV-1 RNA Qualitative Assay and a quantitative viral load measurement was not taken at that day.
(PDF)

S19 Fig All model fits to study participant 29. We provide the fitted curves of the four models (solid lines) to the viral load measurements (points). The black line represents the Standard model, the green line shows the Density-dependent cell death model, the pink line shows the MOI model and orange line represents the Density-dependent cell death & MOI model. Time 0 is the day of the first positive result in the Aptima HIV-1 RNA Qualitative Assay and a quantitative viral load measurement was not taken at that day.
(PDF)

S20 Fig All model fits to study participant 31. We provide the fitted curves of the four models (solid lines) to the viral load measurements (points). The black line represents the Standard model, the green line shows the Density-dependent cell death model, the pink line shows the MOI model and orange line represents the Density-dependent cell death & MOI model. Time 0 is the day of the first positive result in the Aptima HIV-1 RNA Qualitative Assay and a quantitative viral load measurement was not taken at that day.
(PDF)

S21 Fig All model fits to study participant 32. We provide the fitted curves of the four models (solid lines) to the viral load measurements (points). The black line represents the Standard model, the green line shows the Density-dependent cell death model, the pink line shows the MOI model and orange line represents the Density-dependent cell death & MOI model. Time 0 is the day of the first positive result in the Aptima HIV-1 RNA Qualitative Assay and a quantitative viral load measurement was not taken at that day.
(PDF)

S22 Fig All model fits to study participant 33. We provide the fitted curves of the four models (solid lines) to the viral load measurements (points). The black line represents the Standard model, the green line shows the Density-dependent cell death model, the pink line shows the MOI model and orange line represents the Density-dependent cell death & MOI model. Time 0 is the day of the first positive result in the Aptima HIV-1 RNA Qualitative Assay and a quantitative viral load measurement was not taken at that day.
(PDF)

S23 Fig All model fits to study participant 34. We provide the fitted curves of the four models (solid lines) to the viral load measurements (points). The black line represents the Standard model, the green line shows the Density-dependent cell death model, the pink line shows the MOI model and orange line represents the Density-dependent cell death & MOI model. Time 0 is the day of the first positive result in the Aptima HIV-1 RNA Qualitative Assay and a quantitative viral load measurement was not taken at that day.
(PDF)

S24 Fig All model fits to study participant 37. We provide the fitted curves of the four models (solid lines) to the viral load measurements (points). The black line represents the Standard model, the green line shows the Density-dependent cell death model, the pink line shows the MOI model and orange line represents the Density-dependent cell death & MOI model. Time 0 is the day of the first positive result in the Aptima HIV-1 RNA Qualitative Assay and a quantitative viral load measurement was not taken at that day.
(PDF)

S25 Fig All model fits to study participant 40. We provide the fitted curves of the four models (solid lines) to the viral load measurements (points). The black line represents the Standard model, the green line shows the Density-dependent cell death model, the pink line shows the MOI model and orange line represents the Density-dependent cell death & MOI model. Time 0 is the day of the first positive result in the Aptima HIV-1 RNA Qualitative Assay and a quantitative viral load measurement was not taken at that day.
(PDF)

S26 Fig All model fits to study participant 41. We provide the fitted curves of the four models (solid lines) to the viral load measurements (points). The black line represents the Standard model, the green line shows the Density-dependent cell death model, the pink line shows the MOI model and orange line represents the Density-dependent cell death & MOI model. Time 0 is the day of the first positive result in the Aptima HIV-1 RNA Qualitative Assay and a quantitative viral load measurement was not taken at that day.
(PDF)

S27 Fig All model fits to study participant 42. We provide the fitted curves of the four models (solid lines) to the viral load measurements (points). The black line represents the Standard model, the green line shows the Density-dependent cell death model, the pink line shows the MOI model and orange line represents the Density-dependent cell death & MOI model. Time 0 is the day of the first positive result in the Aptima HIV-1 RNA Qualitative Assay and a quantitative viral load measurement was not taken at that day.
(PDF)

S28 Fig All model fits to study participant 44. We provide the fitted curves of the four models (solid lines) to the viral load measurements (points). The black line represents the Standard model, the green line shows the Density-dependent cell death model, the pink line shows the MOI model and orange line represents the Density-dependent cell death & MOI model. Time 0 is the day of the first positive result in the Aptima HIV-1 RNA Qualitative Assay and a quantitative viral load measurement was not taken at that day.
(PDF)

S29 Fig All model fits to study participant 46. We provide the fitted curves of the four models (solid lines) to the viral load measurements (points). The black line represents the Standard model, the green line shows the Density-dependent cell death model, the pink line shows the MOI model and orange line represents the Density-dependent cell death & MOI model. Time 0 is the day of the first positive result in the Aptima HIV-1 RNA Qualitative Assay and a quantitative viral load measurement was not taken at that day.
(PDF)

S30 Fig All model fits to study participant 48. We provide the fitted curves of the four models (solid lines) to the viral load measurements (points). The black line represents the Standard model, the green line shows the Density-dependent cell death model, the pink line shows the MOI model and orange line represents the Density-dependent cell death & MOI model. Time 0 is the day of the first positive result in the Aptima HIV-1 RNA Qualitative Assay and a quantitative viral load measurement was not taken at that day.
(PDF)

S31 Fig All model fits to study participant 49. We provide the fitted curves of the four models (solid lines) to the viral load measurements (points). The black line represents the Standard model, the green line shows the Density-dependent cell death model, the pink line shows the MOI model and orange line represents the Density-dependent cell death & MOI model. Time 0 is the day of the first positive result in the Aptima HIV-1 RNA Qualitative Assay and a quantitative viral load measurement was not taken at that day.
(PDF)

S32 Fig All model fits to study participant 52. We provide the fitted curves of the four models (solid lines) to the viral load measurements (points). The black line represents the Standard model, the green line shows the Density-dependent cell death model, the pink line shows the MOI model and orange line represents the Density-dependent cell death & MOI model. Time 0 is the day of the first positive result in the Aptima HIV-1 RNA Qualitative Assay and a quantitative viral load measurement was not taken at that day.
(PDF)

S33 Fig All model fits to study participant 55. We provide the fitted curves of the four models (solid lines) to the viral load measurements (points). The black line represents the Standard model, the green line shows the Density-dependent cell death model, the pink line shows the MOI model and orange line represents the Density-dependent cell death & MOI model. Time 0 is the day of the first positive result in the Aptima HIV-1 RNA Qualitative Assay and a quantitative viral load measurement was not taken at that day.
(PDF)

S34 Fig All model fits to study participant 57. We provide the fitted curves of the four models (solid lines) to the viral load measurements (points). The black line represents the Standard model, the green line shows the Density-dependent cell death model, the pink line shows the MOI model and orange line represents the Density-dependent cell death & MOI model. Time 0 is the day of the first positive result in the Aptima HIV-1 RNA Qualitative Assay and a quantitative viral load measurement was not taken at that day.
(PDF)

S35 Fig All model fits to study participant 58. We provide the fitted curves of the four models (solid lines) to the viral load measurements (points). The black line represents the Standard model, the green line shows the Density-dependent cell death model, the pink line shows the MOI model and orange line represents the Density-dependent cell death & MOI model. Time 0 is the day of the first positive result in the Aptima HIV-1 RNA Qualitative Assay and a quantitative viral load measurement was not taken at that day.
(PDF)

S36 Fig All model fits to study participant 59. We provide the fitted curves of the four models (solid lines) to the viral load measurements (points). The black line represents the Standard model, the green line shows the Density-dependent cell death model, the pink line shows the MOI model and orange line represents the Density-dependent cell death & MOI model. Time 0 is the day of the first positive result in the Aptima HIV-1 RNA Qualitative Assay and a quantitative viral load measurement was not taken at that day.
(PDF)

S37 Fig All model fits to study participant 61. We provide the fitted curves of the four models (solid lines) to the viral load measurements (points). The black line represents the Standard model, the green line shows the Density-dependent cell death model, the pink line shows the MOI model and orange line represents the Density-dependent cell death & MOI model. Time 0 is the day of the first positive result in the Aptima HIV-1 RNA Qualitative Assay and a quantitative viral load measurement was not taken at that day.
(PDF)

S38 Fig All model fits to study participant 62. We provide the fitted curves of the four models (solid lines) to the viral load measurements (points). The black line represents the Standard model, the green line shows the Density-dependent cell death model, the pink line shows the MOI model and orange line represents the Density-dependent cell death & MOI model. Time 0 is the day of the first positive result in the Aptima HIV-1 RNA Qualitative Assay and a quantitative viral load measurement was not taken at that day.
(PDF)

S39 Fig All model fits to study participant 64. We provide the fitted curves of the four models (solid lines) to the viral load measurements (points). The black line represents the Standard model, the green line shows the Density-dependent cell death model, the pink line shows the MOI model and orange line represents the Density-dependent cell death & MOI model. Time 0 is the day of the first positive result in the Aptima HIV-1 RNA Qualitative Assay and a quantitative viral load measurement was not taken at that day.
(PDF)

S40 Fig All model fits to study participant 65. We provide the fitted curves of the four models (solid lines) to the viral load measurements (points). The black line represents the Standard model, the green line shows the Density-dependent cell death model, the pink line shows the MOI model and orange line represents the Density-dependent cell death & MOI model. Time 0 is the day of the first positive result in the Aptima HIV-1 RNA Qualitative Assay and a quantitative viral load measurement was not taken at that day.
(PDF)

S41 Fig All model fits to study participant 67. We provide the fitted curves of the four models (solid lines) to the viral load measurements (points). The black line represents the Standard model, the green line shows the Density-dependent cell death model, the pink line shows the MOI model and orange line represents the Density-dependent cell death & MOI model. Time 0 is the day of the first positive result in the Aptima HIV-1 RNA Qualitative Assay and a quantitative viral load measurement was not taken at that day.
(PDF)

S42 Fig All model fits to study participant 71. We provide the fitted curves of the four models (solid lines) to the viral load measurements (points). The black line represents the Standard model, the green line shows the Density-dependent cell death model, the pink line shows the MOI model and orange line represents the Density-dependent cell death & MOI model. Time 0 is the day of the first positive result in the Aptima HIV-1 RNA Qualitative Assay and a quantitative viral load measurement was not taken at that day.
(PDF)

S43 Fig All model fits to study participant 73. We provide the fitted curves of the four models (solid lines) to the viral load measurements (points). The black line represents the Standard model, the green line shows the Density-dependent cell death model, the pink line shows the MOI model and orange line represents the Density-dependent cell death & MOI model. Time 0 is the day of the first positive result in the Aptima HIV-1 RNA Qualitative Assay and a quantitative viral load measurement was not taken at that day.
(PDF)

## References

[1] WHO. HIV; 2022. apps.who.int/iris/handle/10665/342899.

[2] Fauci AS, Marston HD. Ending AIDS—is an HIV vaccine necessary? New England Journal of Medicine. 2014;370(6):495–498. doi: 10.1056/NEJMp1313771 24499210

[3] Burton DR. Advancing an HIV vaccine; advancing vaccinology. Nature Reviews Immunology. 2019;19(2):77–78. doi: 10.1038/s41577-018-0103-6 30560910 PMC6425752

[4] Krebs SJ, Ananworanich J. Immune activation during acute HIV infection and the impact of early antiretroviral therapy. Current Opinion in HIV and AIDS. 2016;11(2):163–172. doi: 10.1097/COH.0000000000000228 26599167

[5] Kaufmann DE, Walker BD. PD-1 and CTLA-4 inhibitory cosignaling pathways in HIV infection and the potential for therapeutic intervention. The Journal of Immunology. 2009;182(10):5891–5897. doi: 10.4049/jimmunol.0803771 19414738 PMC3726306

[6] Lindbäck S, Karlsson AC, Mittler J, Blaxhult A, Carlsson M, Briheim G, et al. Viral dynamics in primary HIV-1 infection. AIDS. 2000;14(15):2283–2291. doi: 10.1097/00002030-200010200-00009 11089616

[7] Burg D, Rong L, Neumann AU, Dahari H. Mathematical modeling of viral kinetics under immune control during primary HIV-1 infection. Journal of Theoretical Biology. 2009;259(4):751–759. doi: 10.1016/j.jtbi.2009.04.010 19389409

[8] Koup R, Safrit JT, Cao Y, Andrews CA, McLeod G, Borkowsky W, et al. Temporal association of cellular immune responses with the initial control of viremia in primary Human Immunodeficiency Virus Type 1 syndrome. Journal of Virology. 1994;68(7):4650–4655. doi: 10.1128/jvi.68.7.4650-4655.1994 8207839 PMC236393

[9] Phillips AN. Reduction of HIV concentration during acute infection: independence from a specific immune response. Science. 1996;271(5248):497–499. doi: 10.1126/science.271.5248.497 8560262

[10] Stafford MA, Corey L, Cao Y, Daar ES, Ho DD, Perelson AS. Modeling plasma virus concentration during primary HIV infection. Journal of Theoretical Biology. 2000;203(3):285–301. doi: 10.1006/jtbi.2000.1076 10716909

[11] Perelson AS, Neumann AU, Markowitz M, Leonard JM, Ho DD. HIV-1 dynamics in vivo: virion clearance rate, infected cell life-span, and viral generation time. Science. 1996;271(5255):1582–1586. doi: 10.1126/science.271.5255.1582 8599114

[12] Perelson AS, Essunger P, Cao Y, Vesanen M, Hurley A, Saksela K, et al. Decay characteristics of HIV-1-infected compartments during combination therapy. Nature. 1997;387:188–191. doi: 10.1038/387188a0 9144290

[13] Ribeiro RM, Qin L, Chavez LL, Li D, Self SG, Perelson AS. Estimation of the initial viral growth rate and basic reproductive number during acute HIV-1 infection. Journal of Virology. 2010;84(12):6096–6102. doi: 10.1128/JVI.00127-10 20357090 PMC2876646

[14] Mittler JE, Markowitz M, Ho DD, Perelson AS. Improved estimates for H1V-1 clearance rate and intracellular delay. AIDS. 1999;13(11):1415. doi: 10.1097/00002030-199907300-00023 10449298

[15] Markowitz M, Louie M, Hurley A, Sun E, Di Mascio M, Perelson AS, et al. A novel antiviral intervention results in more accurate assessment of Human Immunodeficiency Virus Type 1 replication dynamics and T-cell decay in vivo. Journal of Virology. 2003;77(8):5037–5038. doi: 10.1128/JVI.77.8.5037-5038.2003 12663814 PMC152136

[16] Chen HY, Di Mascio M, Perelson AS, Ho DD, Zhang L. Determination of virus burst size in vivo using a single-cycle SIV in rhesus macaques. Proceedings of the National Academy of Sciences. 2007;104(48):19079–19084. doi: 10.1073/pnas.0707449104 18025463 PMC2141911

[17] Vaidya NK, Ribeiro RM, Miller CJ, Perelson AS. Viral dynamics during primary simian immunodeficiency virus infection: effect of time-dependent virus infectivity. Journal of Virology. 2010;84(9):4302–4310. doi: 10.1128/JVI.02284-09 20147390 PMC2863724

[18] Dixit NM, Perelson AS. HIV dynamics with multiple infections of target cells. Proceedings of the National Academy of Sciences. 2005;102(23):8198–8203. doi: 10.1073/pnas.0407498102 15928092 PMC1149399

[19] Holte SE, Melvin AJ, Mullins JI, Tobin NH, Frenkel LM. Density-dependent decay in HIV-1 dynamics. JAIDS Journal of Acquired Immune Deficiency Syndromes. 2006;41(3):266–276. doi: 10.1097/01.qai.0000199233.69457.e4 16540927

[20] Robb ML, Eller LA, Kibuuka H, Rono K, Maganga L, Nitayaphan S, et al. Prospective study of acute HIV-1 infection in adults in East Africa and Thailand. New England Journal of Medicine. 2016;374(22):2120–2130. doi: 10.1056/NEJMoa1508952 27192360 PMC5111628

[21] Reeves DB, Rolland M, Dearlove BL, Li Y, Robb ML, Schiffer JT, et al. Timing HIV infection with a simple and accurate population viral dynamics model. Journal of the Royal Society Interface. 2021;18(179):20210314. doi: 10.1098/rsif.2021.0314 34186015 PMC8241492

[22] Kalams SA, Goulder PJ, Shea AK, Jones NG, Trocha AK, Ogg GS, et al. Levels of Human Immunodeficiency Virus Type 1-specific cytotoxic T-lymphocyte effector and memory responses decline after suppression of viremia with highly active antiretroviral therapy. Journal of Virology. 1999;73(8):6721–6728. doi: 10.1128/jvi.73.8.6721-6728.1999 10400770 PMC112757

[23] Bonhoeffer S, May RM, Shaw GM, Nowak MA. Virus dynamics and drug therapy. Proceedings of the National Academy of Sciences. 1997;94(13):6971–6976. doi: 10.1073/pnas.94.13.6971 PMC21269 9192676

[24] Bonhoeffer S, Rembiszewski M, Ortiz GM, Nixon DF. Risks and benefits of structured antiretroviral drug therapy interruptions in HIV-1 infection. AIDS. 2000;14(15):2313–2322. doi: 10.1097/00002030-200010200-00012 11089619

[25] Wodarz D, Nowak MA. Specific therapy regimes could lead to long-term immunological control of HIV. Proceedings of the National Academy of Sciences. 1999;96(25):14464–14469. doi: 10.1073/pnas.96.25.14464 10588728 PMC24459

[26] Conway JM, Perelson AS. Post-treatment control of HIV infection. Proceedings of the National Academy of Sciences. 2015;112(17):5467–5472. doi: 10.1073/pnas.1419162112 25870266 PMC4418889

[27] Wodarz D, Nowak MA. Mathematical models of HIV pathogenesis and treatment. BioEssays. 2002;24(12):1178–1187. doi: 10.1002/bies.10196 12447982

[28] Conway JM, Ribeiro RM. Modeling the immune response to HIV infection. Current Opinion in Systems Biology. 2018;12:61–69. doi: 10.1016/j.coisb.2018.10.006 31463420 PMC6713454

[29] Levy DN, Aldrovandi GM, Kutsch O, Shaw GM. Dynamics of HIV-1 recombination in its natural target cells. Proceedings of the National Academy of Sciences. 2004;101(12):4204–4209. doi: 10.1073/pnas.0306764101 15010526 PMC384719

[30] Song H, Giorgi EE, Ganusov VV, Cai F, Athreya G, Yoon H, et al. Tracking HIV-1 recombination to resolve its contribution to HIV-1 evolution in natural infection. Nature communications. 2018;9(1):1928. doi: 10.1038/s41467-018-04217-5 29765018 PMC5954121

[31] Kouyos RD, Fouchet D, Bonhoeffer S. Recombination and drug resistance in HIV: population dynamics and stochasticity. Epidemics. 2009;1(1):58–69. doi: 10.1016/j.epidem.2008.11.001 21352751

[32] Koelle K, Farrell AP, Brooke CB, Ke R. Within-host infectious disease models accommodating cellular coinfection, with an application to influenza. Virus Evolution. 2019;5(2):vez018. doi: 10.1093/ve/vez018 31304043 PMC6613536

[33] Dixit NM, Perelson AS. Multiplicity of Human Immunodeficiency Virus infections in lymphoid tissue. Journal of Virology. 2004;78(16):8942–8945. doi: 10.1128/JVI.78.16.8942-8945.2004 15280505 PMC479058

[34] Cummings KW, Levy DN, Wodarz D. Increased burst size in multiply infected cells can alter basic virus dynamics. Biology Direct. 2012;7(1):1–15. doi: 10.1186/1745-6150-7-16 22569346 PMC3482397

[35] Wodarz D, Levy DN. Effect of multiple infection of cells on the evolutionary dynamics of HIV in vivo: implications for host adaptation mechanisms. Experimental Biology and Medicine. 2011;236(8):926–937. doi: 10.1258/ebm.2011.011062 21768164

[36] Guo T, Qiu Z, Kitagawa K, Iwami S, Rong L. Modeling HIV multiple infection. Journal of Theoretical Biology. 2021;509:110502. doi: 10.1016/j.jtbi.2020.110502 32998053

[37] Roberts M, Smith G, Grenfell B. Mathematical Models for Macroparasites of Wildlife; Ecology of Infectious Diseases in Natural Populations. Cambridge: Cambridge University Press. 1995; p. 177–208.

[38] Anderson R, May RM. Regulation and stability of host-parasite population interactions. Journal of Animal Ecology. 1978;47(1):219–247. doi: 10.2307/3933

[39] Anderson R, May R. Infectious Diseases of Humans: Dynamics and Control. Oxford: Oxford University Press; 1992.

[40] Jung A, Maier R, Vartanian JP, Bocharov G, Jung V, Fischer U, et al. Multiply infected spleen cells in HIV patients. Nature. 2002;418(6894):144–144. 12110879 10.1038/418144a

[41] Dang Q, Chen J, Unutmaz D, Coffin JM, Pathak VK, Powell D, et al. Nonrandom HIV-1 infection and double infection via direct and cell-mediated pathways. Proceedings of the National Academy of Sciences. 2004;101(2):632–637. doi: 10.1073/pnas.0307636100 14707263 PMC327199

[42] Chen J, Dang Q, Unutmaz D, Pathak VK, Maldarelli F, Powell D, et al. Mechanisms of nonrandom Human Immunodeficiency Virus Type 1 infection and double infection: preference in virus entry is important but is not the sole factor. Journal of Virology. 2005;79(7):4140–4149. doi: 10.1128/JVI.79.7.4140-4149.2005 15767415 PMC1061529

[43] Sachsenberg N, Perelson A, Yerly S, Schockmel G, Leduc D, Hirschel B, et al. Turnover of CD4+ and CD8+ T lymphocytes in HIV-1 infection as measured by Ki-67 antigen. The Journal of experimental medicine. 1998;187:1295–303. doi: 10.1084/jem.187.8.1295 9547340 PMC2212238

[44] Torsney-Weir T. Optim functions. https://CRANR-projectorg/package=optimfunctions. 2017;.

[45] Stoica P, Selen Y. Model-order selection: a review of information criterion rules. IEEE Signal Processing Magazine. 2004;21(4):36–47. doi: 10.1109/MSP.2004.1311138

[46] Sugiura N. Further analysis of the data by Akaike’s information criterion and the finite corrections: Further analysis of the data by akaike’s. Communications in Statistics-Theory and Methods. 1978;7(1):13–26. doi: 10.1080/03610927808827599

[47] Anderson D, Burnham K. Model selection and multi-model inference. Second NY: Springer-Verlag. 2004;63(2020):10.

[48] Korber BT, Kunstman KJ, Patterson BK, Furtado M, McEvilly MM, Levy R, et al. Genetic differences between blood- and brain-derived viral sequences from Human Immunodeficiency Virus Type 1-infected patients: evidence of conserved elements in the V3 region of the envelope protein of brain-derived sequences. Journal of Virology. 1994;68(11):7467–7481. doi: 10.1128/jvi.68.11.7467-7481.1994 7933130 PMC237189

[49] Law K, Komarova N, Yewdall A, Lee R, Herrera O, Wodarz D, et al. In Vivo HIV-1 cell-to-cell transmission promotes multicopy micro-compartmentalized infection. Cell. 2016;15:2771–83. 27292632 10.1016/j.celrep.2016.05.059

[50] Ogg GS, Jin X, Bonhoeffer S, Dunbar PR, Nowak MA, Monard S, et al. Quantitation of HIV-1-specific cytotoxic T lymphocytes and plasma load of viral RNA. Science. 1998;279(5359):2103–2106. doi: 10.1126/science.279.5359.2103 9516110

[51] Vuilleumier S, Bonhoeffer S. Contribution of recombination to the evolutionary history of HIV. Current Opinion in HIV and AIDS. 2015;10(2):84–89. doi: 10.1097/COH.0000000000000137 25565174

[52] Fraser C. HIV recombination: what is the impact on antiretroviral therapy? Journal of the Royal Society Interface. 2005;2(5):489–503. doi: 10.1098/rsif.2005.0064 16849208 PMC1618498

[53] Bretscher MT, Althaus CL, Müller V, Bonhoeffer S. Recombination in HIV and the evolution of drug resistance: for better or for worse? Bioessays. 2004;26(2):180–188. doi: 10.1002/bies.10386 14745836

[54] Romero EV, Feder AF. Elevated HIV viral load is associated with higher recombination rate in vivo. bioRxiv. 2023; p. 2023–05. doi: 10.1101/2023.05.05.539643 38197289 PMC10777272

[55] Ribeiro RM, Mohri H, Ho DD, Perelson AS. In vivo dynamics of T cell activation, proliferation, and death in HIV-1 infection: why are CD4+ but not CD8+ T cells depleted? Proceedings of the National Academy of Sciences. 2002;99(24):15572–15577. doi: 10.1073/pnas.242358099 PMC137758 12434018

[56] Yates A, Stark J, Klein N, Antia R, Callard R. Understanding the slow depletion of memory CD4+ T cells in HIV infection. PLoS medicine. 2007;4(5):e177. doi: 10.1371/journal.pmed.0040177 17518516 PMC1872038

[57] Nowak MA, Anderson RM, McLean AR, Wolfs TF, Goudsmit J, May RM. Antigenic diversity thresholds and the development of AIDS. Science. 1991;254(5034):963–969. doi: 10.1126/science.1683006 1683006

[58] Regoes RR, Wodarz D, Nowak MA. Virus dynamics: the effect of target cell limitation and immune responses on virus evolution. Journal of theoretical biology. 1998;191(4):451–462. doi: 10.1006/jtbi.1997.0617 9631576

[59] Conway JM, Coombs D. A stochastic model of latently infected cell reactivation and viral blip generation in treated HIV patients. PLoS computational biology. 2011;7(4):e1002033. doi: 10.1371/journal.pcbi.1002033 21552334 PMC3084212

[60] Conway JM, Perelson AS. Residual viremia in treated HIV+ individuals. PLoS computational biology. 2016;12(1):e1004677. doi: 10.1371/journal.pcbi.1004677 26735135 PMC4703306

[61] Rong L, Perelson AS. Asymmetric division of activated latently infected cells may explain the decay kinetics of the HIV-1 latent reservoir and intermittent viral blips. Mathematical biosciences. 2009;217(1):77–87. doi: 10.1016/j.mbs.2008.10.006 18977369 PMC2657607

[62] Rong L, Perelson AS. Modeling latently infected cell activation: viral and latent reservoir persistence, and viral blips in HIV-infected patients on potent therapy. PLoS computational biology. 2009;5(10):e1000533. doi: 10.1371/journal.pcbi.1000533 19834532 PMC2752194

[63] Hill AL, Rosenbloom DI, Fu F, Nowak MA, Siliciano RF. Predicting the outcomes of treatment to eradicate the latent reservoir for HIV-1. Proceedings of the National Academy of Sciences. 2014;111(37):13475–13480. doi: 10.1073/pnas.1406663111 25097264 PMC4169952

[64] Wang A, Li MY. Viral dynamics of HIV-1 with CTL immune response. Discrete & Continuous Dynamical Systems-Series B. 2021;26(4).

[65] Naik PA, Zu J, Owolabi KM. Modeling the mechanics of viral kinetics under immune control during primary infection of HIV-1 with treatment in fractional order. Physica A: statistical mechanics and its applications. 2020;545:123816. doi: 10.1016/j.physa.2019.123816

[66] Cao Y, Cartwright EK, Silvestri G, Perelson AS. CD8+ lymphocyte control of SIV infection during antiretroviral therapy. PLoS pathogens. 2018;14(10):e1007350. doi: 10.1371/journal.ppat.1007350 30308068 PMC6199003

[67] Conway JM, Konrad BP, Coombs D. Stochastic analysis of pre-and postexposure prophylaxis against HIV infection. SIAM Journal on Applied Mathematics. 2013;73(2):904–928. doi: 10.1137/120876800

[68] Marozsan AJ, Fraundorf E, Abraha A, Baird H, Moore D, Troyer R, et al. Relationships between infectious titer, capsid protein levels, and reverse transcriptase activities of diverse human immunodeficiency virus type 1 isolates. Journal of virology. 2004;78(20):11130–11141. doi: 10.1128/JVI.78.20.11130-11141.2004 15452233 PMC521859

[69] Herz A, Bonhoeffer S, Anderson RM, May RM, Nowak MA. Viral dynamics in vivo: limitations on estimates of intracellular delay and virus decay. Proceedings of the National Academy of Sciences. 1996;93(14):7247–7251. doi: 10.1073/pnas.93.14.7247 8692977 PMC38968

[70] Dixit NM, Markowitz M, Ho DD, Perelson AS. Estimates of intracellular delay and average drug efficacy from viral load data of HIV-infected individuals under antiretroviral therapy. Antiviral therapy. 2004;9(2):237–246. doi: 10.1177/135965350400900216 15134186

[71] Kim H, Perelson AS. Viral and latent reservoir persistence in HIV-1–infected patients on therapy. PLoS computational biology. 2006;2(10):e135. doi: 10.1371/journal.pcbi.0020135 17040122 PMC1599767

[72] Lythgoe KA, Fraser C. New insights into the evolutionary rate of HIV-1 at the within-host and epidemiological levels. Proceedings of the Royal Society B: Biological Sciences. 2012;279(1741):3367–3375. doi: 10.1098/rspb.2012.0595 22593106 PMC3385732

[73] McLean AR, Nowak MA. Competition between zidovudine-sensitive and zidovudine-resistant strains of HIV. Aids. 1992;6:71–79. doi: 10.1097/00002030-199201000-00009 1543568

