# Supplementary material for: Modeling dynamics of acute HIV infection incorporating density-dependent cell death and multiplicity of infection
Source: PLoS Comput Biol. 2024 Jun 7;20(6):e1012129. doi: 10.1371/journal.pcbi.1012129 (PMC11189221; doi:10.1371/journal.pcbi.1012129)
Supplement: S1 Text — (DOCX) [file pcbi.1012129.s001.docx]

**S1 Text: Derivation of simplified terms in the *Density-Dependent Cell Death & MOI* model**

In the main text, we presented two published models: the DDDI model which aims to capture the nonlinear log decay of the infected cell population and the MOI model (Eq 4-6) which incorporates the effects of cellular coinfection and burst size heterogeneity. We then provide an alternate model combining these two effects. To account for both cellular coinfection and density-dependence of infected cell death, in a relatively simple model, we use the MOI model as a basis and incorporate density-dependence with rate γ.

Recall the MOI model,

$$\frac{dH}{dt}=a-bH-H\sum_{i=0}^{\infty} \alpha_{i}p_{i}$$

$$\frac{dV}{dt}=H\sum_{i=0}^{\infty} \lambda_{i}p_{i} -\eta V-\beta HV$$

$$\frac{dP}{dt}=\beta HV -bH\sum_{i=0}^{\infty} ip_{i}-H\sum_{i=0}^{\infty} {i\alpha}_{i}p_{i}$$

We assume that the sheer number of infected cells enhances the death rate. That number is reflected in the term $H\sum_{i=0}^{\infty} \alpha_{i}p_{i}$ in the target cell equation. Similarly, internalized virus P is removed at a density-dependent way from the term $H\sum_{i=0}^{\infty} {i\alpha}_{i}p_{i}$. To permit density-dependent death, these terms $H\left( \sum_{i=0}^{\infty} \alpha_{i}p_{i} \right)^{\gamma}$ and ${H\left( \sum_{i=0}^{\infty} {i \alpha}_{i}p_{i} \right)}^{\gamma}$ respectively.

We again assume that the death rate of infected cells *α_i_* scales linearly with MOI *i*. Therefore,

$$H\left( \sum_{i=0}^{\infty} \alpha_{i}p_{i} \right)^{\gamma}= H\left( \sum_{i=0}^{\infty} i\alpha p_{i} \right)^{\gamma}$$

$= H\alpha^{\gamma}\left( \sum_{i=0}^{\infty} ip_{i} \right)^{\gamma}$

$= H\alpha^{\gamma}\left( \frac{P}{H} \right)^{\gamma}$

$= \alpha^{\gamma}\frac{P^{\gamma}}{H^{\gamma-1}}$

and

$$H\left( \sum_{i=0}^{\infty} {i \alpha}_{i}p_{i} \right)^{\gamma}= H\left( \sum_{i=0}^{\infty} i^{2}\alpha p_{i} \right)^{\gamma}$$

= $H\alpha^{\gamma}\left( \sum_{i=0}^{\infty} i^{2}p_{i} \right)^{\gamma}$

= $H\alpha^{\gamma}\left[ \frac{P}{H}+ \left( \frac{P}{H} \right)^{2}+\frac{1+k}{k} \right]^{\gamma}$

= $\alpha^{\gamma}{\frac{P^{\gamma}}{H^{\gamma-1}}\left[ 1+ \frac{P}{H}\left( \frac{1+k}{k} \right) \right]}^{\gamma}$

Therefore, the equations of the Density-dependent cell death & MOI model become the following:

$$\frac{dH}{dt}=a-bH-\alpha^{\gamma}\frac{P^{\gamma}}{H^{\gamma-1}}$$

$$\frac{dV}{dt}=\lambda P -\eta V-\beta HV$$

$$\frac{dP}{dt}=\beta HV -bP-\alpha^{\gamma}{\frac{P^{\gamma}}{H^{\gamma-1}}\left[ 1+ \frac{P}{H}\left( \frac{1+k}{k} \right) \right]}^{\gamma}$$
