# Supplementary material for: Modeling dynamics of acute HIV infection incorporating density-dependent cell death and multiplicity of infection
Source: PLoS Comput Biol. 2024 Jun 7;20(6):e1012129. doi: 10.1371/journal.pcbi.1012129 (PMC11189221; doi:10.1371/journal.pcbi.1012129)
Supplement: S2 Text — (DOCX) [file pcbi.1012129.s002.docx]

**S2 Text: Burst size calculation**

In Figure 5 in the main text, we show the model-predicted infected cell burst sizes. Here we provide details as to how those quantities were computed. Burst size is defined as the number of virions produced throughout the lifetime of an infected cell. In the case of the Standard model,

$$Burst size=\frac{viral production}{death of infected cells}= \frac{pI}{\delta I}= \frac{p}{\delta}$$

Similarly, for the MOI model, the term that serves as a proxy for the death of infected cells is decrease in target cells due to virus-induced mortality. Therefore,

$$Burst size=\frac{viral production}{death of infected cells}= \frac{\lambda P}{\alpha P}= \frac{\lambda}{\alpha}$$

For both the Standard and the MOI models, burst size remains constant over time.

For the two models that incorporate density-dependence in the death rate of infected cells, estimation of burst size is more complicated. In the case of the Density-dependent Death of Infected Cells (DDDI) model,

$$Burst size=\frac{viral production}{death of infected cells}= \frac{pI}{\delta I^{\gamma}}= \frac{pI^{1-\gamma}}{\delta}$$

Burst size changes over time, due to the presence of infected cells, I. Minimum burst size occurs for the maximum I, whereas minimum burst size occurs for I=0, becoming $\frac{p}{\delta}$.

For the Density-dependent infected cell death & MOI model,

$$Burst size=\frac{viral production}{death of infected cells}= \frac{\lambda P}{\alpha^{\gamma}\frac{P^{\gamma}}{H^{\gamma-1}}}= \frac{\lambda H^{\gamma-1}P^{1-\gamma}}{\alpha^{\gamma}}$$

Again, burst size is non-constant. For both the DDDI model and the DDDI&MOI we report the minimum and maximum values.
