# Supplementary material for: Modeling dynamics of acute HIV infection incorporating density-dependent cell death and multiplicity of infection
Source: PLoS Comput Biol. 2024 Jun 7;20(6):e1012129. doi: 10.1371/journal.pcbi.1012129 (PMC11189221; doi:10.1371/journal.pcbi.1012129)
Supplement: S1 Table — (DOCX) [file pcbi.1012129.s003.docx]

Table S1: Growth, decay, peak and setpoint linear model parameter estimation and Standard-model derived R_0_ for each study participant. We R_0_ compute as R_0_= (1+r/d)e^rτ^, where r=growth rate, d=decay rate and τ= 1 day is the eclipse phase. R_0_ is derived from Ribeiro et al. (2010) [1]. We also report the least squares estimate, which is the objective function used in *optim* for the goodness of fit for each linear model. “NA” signifies that the measurement was not estimated. Finally, we report mean, median and interquartile range (IQR) for the reader’s reference.

[1] Ribeiro RM, Qin L, Chavez LL, Li D, Self SG, Perelson AS. Estimation of the initial viral growth rate and basic reproductive number during acute HIV-1 infection. J Virol. 2010 Jun;84(12):6096-102. doi: 10.1128/JVI.00127-10.

| **ID** | **Growth rate** | **Intercept growth model** | **Least squares for growth model** | **Decay rate** | **Intercept decay model** | **Least squares for decay model** | **Setpoint** | **Least squares for setpoint model** | **Peak magnitude** | **Peak timing** | **R_0_** |
| --- | --- | --- | --- | --- | --- | --- | --- | --- | --- | --- | --- |
| 1 | 0.25 | 4.58 | 0.2016 | -0.13 | 8.56 | 0.0819 | 3.9 | 0.0153 | 7.51 | 11 | 3.79 |
| 2 | 0.32 | 3.31 | 0 | -0.14 | 6.72 | 0.5309 | 3.69 | 0.1233 | 5.71 | 7.41 | 4.65 |
| 3 | NA | NA | NA | -0.06 | 6.29 | 0.4739 | 3.6 | 0.2921 | NA | NA | NA |
| 4 | 0.28 | 0.7 | 0.1383 | -0.21 | 9.95 | 0.0507 | 3.81 | 0.4583 | 5.94 | 18.91 | 3.04 |
| 5 | 0.19 | 3.43 | 0 | -0.19 | 8.08 | 8E-04 | 4.3 | 1.0839 | 5.74 | 12.17 | 2.41 |
| 6 | 0.24 | 4.57 | 0.0022 | -0.24 | 9.93 | 0.0409 | 4.9 | 0.1729 | 7.27 | 11.34 | 2.55 |
| 7 | 0.19 | 5.05 | 0 | -0.11 | 7.78 | 0.5335 | 3.38 | 0.2315 | 6.78 | 9.28 | 3.31 |
| 8 | 0.52 | 2.16 | 0 | -0.15 | 8.74 | 0.0529 | 3.9 | 0.8169 | 7.25 | 9.83 | 7.39 |
| 9 | NA | NA | NA | -0.1 | 6.5 | 0.0029 | 2.64 | 0.1699 | NA | NA | NA |
| 10 | NA | NA | NA | -0.23 | 7.82 | 0.1995 | 4.29 | 0.2713 | NA | NA | NA |
| 11 | 0.35 | 2.86 | 0.2604 | -0.18 | 10.34 | 0.4038 | NA | NA | 7.78 | 14.01 | 4.16 |
| 12 | 0.31 | 4.03 | 0.0151 | -0.24 | 11.13 | 0.2202 | 5.2 | 0.513 | 8.07 | 12.92 | 3.17 |
| 13 | NA | NA | NA | NA | NA | NA | NA | NA | NA | NA | NA |
| 14 | NA | NA | NA | -0.07 | 6.41 | 2.6683 | 4.55 | 0.0174 | NA | NA | NA |
| 15 | NA | NA | NA | -0.07 | 5.95 | 3.7063 | 2.28 | 0.4413 | NA | NA | NA |
| 16 | NA | NA | NA | -0.33 | 10.9 | 0.0648 | 3.33 | 2.5103 | NA | NA | NA |
| 17 | NA | NA | NA | -0.08 | 8.44 | 0.1341 | 3.53 | 0.1992 | NA | NA | NA |
| 18 | NA | NA | NA | -0.14 | 6.78 | 0.7755 | 2.51 | 0.0469 | NA | NA | NA |
| 19 | 0.62 | 2.71 | 0 | -0.3 | 11.45 | 0.0033 | NA | NA | 8.61 | 9.55 | 5.7 |
| 20 | 0.17 | 4.38 | 0 | -0.35 | 10.99 | 0.3772 | 3.46 | 0.4849 | 6.68 | 13 | 1.76 |
| 21 | 0.43 | 3.07 | 0.0748 | -0.26 | 11.46 | 0.0652 | 5.08 | 0.8903 | 8.27 | 12.12 | 4.03 |
| 22 | 0.32 | 3.72 | 0.0021 | -0.18 | 7.8 | 0.3531 | 3.2 | 2.8902 | 6.32 | 8.1 | 3.79 |
| 23 | 0.38 | 2.07 | 0.1184 | -0.21 | 8.56 | 0.2414 | 4.08 | 1.9058 | 6.26 | 11.03 | 4.13 |
| 24 | 0.14 | 2.71 | 0.2823 | -0.12 | 8.7 | 6E-04 | NA | NA | 6.01 | 22 | 2.53 |
| 25 | 0.48 | 2.5 | 0 | -0.15 | 8.04 | 0.0671 | 3.57 | 0.3811 | 6.74 | 8.93 | 6.87 |
| 26 | 0.4 | 3.14 | 0 | -0.19 | 9.43 | 0.0144 | 4.54 | 0.0089 | 7.39 | 10.52 | 4.62 |
| 27 | 0.37 | 3.15 | 0.2873 | -0.3 | 12.49 | 0.3569 | 3.7 | 0.1305 | 8.29 | 13.89 | 3.22 |
| 28 | 0.26 | 3.75 | 0.0864 | -0.22 | 9.82 | 0.0783 | 3.09 | 0.6549 | 7.07 | 12.63 | 2.87 |
| 29 | 0.19 | 4.59 | 0.0937 | -0.27 | 10.42 | 0.0033 | 4.32 | 0.1053 | 7.01 | 12.85 | 2.07 |
| 30 | NA | NA | NA | NA | NA | NA | 4.23 | 0.5085 | NA | NA | NA |
| 31 | 0.13 | 4.53 | 0.0011 | -0.27 | 8.08 | 0.4891 | 2.69 | 0.3771 | 5.68 | 9 | 1.68 |
| 32 | 0.21 | 2.63 | 8E-04 | -0.16 | 6.59 | 0.0104 | 3.45 | 0.0072 | 4.84 | 10.72 | 2.78 |
| 33 | 0.43 | 2.15 | 0.0043 | -0.17 | 6.8 | 0.0967 | NA | NA | 5.64 | 8 | 5.53 |
| 34 | 0.46 | 2.67 | 0 | -0.16 | 8.86 | 0.5014 | NA | NA | 7.24 | 10 | 6.02 |
| 35 | NA | NA | NA | NA | NA | NA | 2.49 | 1.3984 | NA | NA | NA |
| 36 | NA | NA | NA | -0.12 | 7.64 | 1.9544 | NA | NA | NA | NA | NA |
| 37 | 0.11 | 3.65 | 0.2605 | -0.05 | 5.64 | 0.1822 | 3.02 | 0.0185 | 5.45 | 17 | 3.81 |
| 38 | NA | NA | NA | NA | NA | NA | NA | NA | NA | NA | NA |
| 39 | NA | NA | NA | NA | NA | NA | NA | NA | NA | NA | NA |
| 40 | 0.48 | 4.27 | 0 | -0.26 | 9.32 | 1.7897 | NA | NA | 7.76 | 9 | 4.62 |
| 41 | 0.32 | 3.66 | 0.014 | -0.24 | 10.73 | 0.5472 | NA | NA | 7.69 | 12.55 | 3.2 |
| 42 | 0.46 | 2.34 | 0.0903 | -0.11 | 8.99 | 0.0093 | 5.29 | 2.0203 | 7.67 | 11.54 | 8 |
| 43 | NA | NA | NA | -0.1 | 6.55 | 0.0016 | 4.96 | 0.2073 | NA | NA | NA |
| 44 | 0.33 | 1.71 | 0.0654 | -0.16 | 7.96 | 0.0183 | 3.78 | 6E-04 | 5.95 | 12.68 | 4.35 |
| 45 | NA | NA | NA | -0.05 | 5.4 | 0.0172 | 4.87 | 0.0081 | NA | NA | NA |
| 46 | 0.16 | 4.07 | 0.2138 | -0.15 | 8.86 | 0.0207 | 4.77 | 0.2143 | 6.53 | 15.74 | 2.41 |
| 47 | NA | NA | NA | NA | NA | NA | NA | NA | NA | NA | NA |
| 48 | 0.34 | 5.27 | 0 | -0.35 | 11.88 | 0.0509 | 5.22 | 0.0695 | 8.54 | 9.56 | 2.79 |
| 49 | 0.43 | 1.56 | 0.0427 | -0.11 | 7.74 | 0.0528 | 4.8 | 0.5065 | 6.46 | 11.53 | 7.41 |
| 50 | NA | NA | NA | -0.05 | 6.52 | 0.0192 | 4.37 | 0.015 | NA | NA | NA |
| 51 | 0.03 | 1.44 | 0.4048 | NA | NA | NA | 4.66 | 1.2892 | NA | NA | NA |
| 52 | 0.29 | 3.14 | 0 | -0.07 | 7.48 | 0.0014 | 5.34 | 0.0871 | 6.66 | 11 | 6.57 |
| 53 | NA | NA | NA | NA | NA | NA | 5.08 | 0.1126 | NA | NA | NA |
| 54 | NA | NA | NA | -0.06 | 6.26 | 0.0018 | 4.57 | 0.9262 | NA | NA | NA |
| 55 | 0.34 | 3.61 | 0.0463 | -0.13 | 8.05 | 0.1456 | 4.01 | 0.004 | 6.79 | 9.47 | 4.96 |
| 56 | NA | NA | NA | NA | NA | NA | NA | NA | NA | NA | NA |
| 57 | 0.39 | 2.75 | 0.0982 | -0.1 | 7.87 | 0.4488 | 4.6 | 0.7161 | 6.86 | 10.39 | 7.5 |
| 58 | 0.45 | 3.06 | 0.1568 | -0.14 | 9.15 | 0.0228 | 5.03 | 0.3094 | 7.69 | 10.39 | 6.49 |
| 59 | 0.36 | 2.35 | 0.2753 | -0.15 | 8.64 | 0.009 | 4.8 | 0.3294 | 6.77 | 12.16 | 4.84 |
| 60 | NA | NA | NA | -0.25 | 5.76 | 0.2266 | 2.78 | 0.927 | NA | NA | NA |
| 61 | 0.28 | 1.21 | 0.2635 | -0.26 | 10.58 | 0.0811 | 3.71 | 0.1297 | 6.14 | 17.4 | 2.8 |
| 62 | 0.54 | 1.95 | 9E-04 | -0.18 | 8.83 | 0.6186 | 4.93 | 0.1899 | 7.13 | 9.51 | 6.97 |
| 63 | NA | NA | NA | -0.19 | 6.42 | 0.0267 | 4.69 | 0.4413 | NA | NA | NA |
| 64 | 0.16 | 3.12 | 0.2906 | -0.05 | 6.67 | 0.0804 | 4.41 | 0.1355 | 6.09 | 18 | 5.35 |
| 65 | 0.32 | 4.62 | 0 | -0.04 | 7.25 | 0.1744 | 4.09 | 0.3025 | 6.95 | 7.3 | 12.16 |
| 66 | 0.05 | 5.3 | 2E-04 | -0.01 | 5.85 | 3E-04 | NA | NA | 5.84 | 11 | 9.72 |
| 67 | 0.55 | 2.65 | 0 | -0.11 | 8.07 | 0.1795 | 5.08 | 0.3339 | 7.16 | 8.13 | 10.36 |
| 68 | NA | NA | NA | -0.19 | 8.16 | 0.1075 | 4.36 | 0.0778 | NA | NA | NA |
| 69 | 0.29 | 2.94 | 0.1319 | -0.12 | 8.64 | 0.1516 | 5.07 | 0.2263 | 6.98 | 13.78 | 4.61 |
| 70 | NA | NA | NA | -0.02 | 5.02 | 0.0026 | 4.14 | 0.2032 | NA | NA | NA |
| 71 | 0.44 | 4.24 | 0.0561 | -0.14 | 9.48 | 0.1257 | 5.63 | 0.1775 | 8.21 | 9.03 | 6.4 |
| 72 | NA | NA | NA | NA | NA | NA | 4.12 | 0.1576 | NA | NA | NA |
| 73 | 0.6 | 1.93 | 0.0321 | -0.18 | 9.68 | 0.509 | 4.33 | 0.6403 | 7.89 | 9.88 | 7.91 |
| 74 | NA | NA | NA | -0.02 | 5.62 | 0.0052 | NA | NA | NA | NA | NA |
| 75 | NA | NA | NA | NA | NA | NA | NA | NA | NA | NA | NA |
| 76 | NA | NA | NA | NA | NA | NA | NA | NA | NA | NA | NA |
| 77 | NA | NA | NA | NA | NA | NA | NA | NA | NA | NA | NA |
| Mean | 0.32 | 3.17 | 0.08 | -0.16 | 8.28 | 0.31 | 4.13 | 0.47 | 6.89 | 11.65 | 4.89 |
| Median | 0.32 | 3.12 | 0.03 | -0.15 | 8.08 | 0.08 | 4.29 | 0.23 | 6.9 | 11 | 4.48 |
| IQR | 0.225 | 2.42 | 0 | -0.21 | 6.7 | 0.01 | 3.58 | 0.11 | 6.17 | 9.52 | 3.07 |
| IQR Q3 | 0.43 | 4.05 | 0.13 | -0.1 | 9.44 | 0.36 | 4.8 | 0.51 | 7.63 | 12.8 | 6.46 |
