# Supplementary material for: Modeling dynamics of acute HIV infection incorporating density-dependent cell death and multiplicity of infection
Source: PLoS Comput Biol. 2024 Jun 7;20(6):e1012129. doi: 10.1371/journal.pcbi.1012129 (PMC11189221; doi:10.1371/journal.pcbi.1012129)
Supplement: S2 Table — Parameter value estimates for the Standard model, along with the number of data points used to fit the model, the negative log likelihood (nll), AIC, BIC and AICc. We also report mean, median and interquartile range (IQR) for the reader reference. (DOCX) [file pcbi.1012129.s004.docx]

Table S2: Parameter value estimates for the Standard model, along with the number of data points used to fit the model, the negative log likelihood (NLL), AIC, BIC and AICc. We also report mean, median and interquartile range (IQR) for the reader reference.

| **ID** | **Log_10_(p)** | **delta** | **c** | **k** | **t_0_** | **n** | **error value** | **NLL** | **AIC** | **BIC** | **AICc** |
| --- | --- | --- | --- | --- | --- | --- | --- | --- | --- | --- | --- |
| 1 | 2.99 | 0.58 | 19.08 | 2.54E-08 | -17.59 | 11 | 0.0788 | 27.4 | 64.8 | 66.79 | 76.8 |
| 2 | 1.08 | 0.33 | 20.04 | 2.4238E-06 | -7.79 | 9 | 0.0837 | 16.78 | 43.57 | 44.55 | 63.57 |
| 4 | 0.75 | 0.35 | 10.97 | 2.23E-06 | -3.5 | 10 | 0.097 | 7.66 | 25.33 | 26.84 | 40.33 |
| 5 | 2.08 | 0.46 | 13 | 3.85E-07 | -4.6 | 8 | 0.0936 | 7.51 | 25.02 | 25.42 | 55.02 |
| 6 | 1.45 | 0.57 | 1.33 | 1.05E-07 | -12.62 | 8 | 0.0643 | 19.43 | 48.86 | 49.26 | 78.86 |
| 7 | 2.49 | 0.53 | 12.05 | 5.27E-08 | -14.7 | 12 | 0.0268 | 25.85 | 61.69 | 64.12 | 71.69 |
| 8 | 2.42 | 0.39 | 16.32 | 1.084E-07 | -4.35 | 9 | 0.0474 | 10.19 | 30.38 | 31.36 | 50.38 |
| 11 | 2.57 | 1.03 | 0.84 | 1.27E-08 | -4.03 | 9 | 0.0279 | 14.04 | 38.07 | 39.06 | 58.07 |
| 12 | 2.61 | 0.86 | 1.14 | 1.22E-08 | -5.21 | 10 | 0.0786 | 18.74 | 47.47 | 48.98 | 62.47 |
| 20 | 1.44 | 0.51 | 5.14 | 4.08E-07 | -5.71 | 8 | 0.2151 | 12.79 | 35.58 | 35.97 | 65.58 |
| 21 | 2.53 | 0.82 | 1.09 | 1.42E-08 | -4.91 | 9 | 0.0839 | 17.29 | 44.59 | 45.57 | 64.59 |
| 22 | 1.59 | 0.48 | 11.89 | 6.87E-07 | -4.89 | 10 | 0.107 | 17.65 | 45.3 | 46.81 | 60.3 |
| 23 | 0.92 | 0.36 | 6.7 | 1.73E-06 | -2.97 | 9 | 0.1841 | 10.47 | 30.93 | 31.92 | 50.93 |
| 24 | 3.45 | 6.13 | 13.99 | 3.29E-08 | -10.6 | 7 | 0.0581 | 14.84 | 39.68 | 39.41 | 99.68 |
| 25 | 2.42 | 0.62 | 20.27 | 1.34E-07 | -6.11 | 9 | 0.0369 | 16.53 | 43.07 | 44.05 | 63.07 |
| 26 | 1.81 | 0.35 | 4.72 | 1.14E-07 | -8.88 | 8 | 0.0615 | 16.16 | 42.33 | 42.72 | 72.33 |
| 27 | 2.55 | 0.94 | 1.2 | 1.33E-08 | -5.15 | 9 | 0.1613 | 17.9 | 45.8 | 46.79 | 65.8 |
| 28 | 2.64 | 0.99 | 10.86 | 4.72E-08 | -11.13 | 10 | 0.0657 | 22.64 | 55.29 | 56.8 | 70.29 |
| 29 | 2.37 | 0.41 | 21.78 | 1.12E-07 | -14.05 | 8 | 0.1089 | 19.02 | 48.04 | 48.44 | 78.04 |
| 31 | 0.81 | 0.83 | 1.96 | 1.13E-06 | -5.87 | 9 | 0.2009 | 17.87 | 45.74 | 46.72 | 65.74 |
| 32 | 0 | 0.39 | 9.84 | 1.08E-05 | -12.66 | 8 | 0.023 | 14.95 | 39.91 | 40.31 | 69.91 |
| 33 | 1.2 | 0.91 | 12.28 | 1.52E-06 | -6.95 | 9 | 0.0385 | 16.67 | 43.33 | 44.32 | 63.33 |
| 34 | 2.29 | 0.73 | 2.94 | 4.18E-08 | -4.52 | 11 | 0.0184 | 17.06 | 44.11 | 46.1 | 56.11 |
| 37 | 1.15 | 0.32 | 19.58 | 1.02E-06 | -24.25 | 13 | 0.0465 | 27.52 | 65.05 | 67.87 | 73.62 |
| 40 | 2.73 | 1.49 | 2.03 | 1.894E-08 | -6.33 | 9 | 0.0551 | 20.55 | 51.1 | 52.08 | 71.1 |
| 41 | 2.35 | 1.13 | 1 | 2.13E-08 | -6.11 | 9 | 0.1432 | 19.1 | 48.2 | 49.19 | 68.2 |
| 42 | 2.78 | 0.29 | 11.9 | 3.19E-08 | -5.07 | 10 | 0.0076 | 17.2 | 44.4 | 45.92 | 59.4 |
| 44 | 0.71 | 0.27 | 7.95 | 2.43E-06 | -3.35 | 11 | 0.1189 | 11.13 | 32.27 | 34.26 | 44.27 |
| 46 | 2.09 | 0.37 | 21.39 | 1.63E-07 | -18.63 | 11 | 0.075 | 25.5 | 61.01 | 63 | 73.01 |
| 48 | 2.49 | 0.63 | 0.98 | 1.27E-08 | -9.32 | 8 | 0.1086 | 19.64 | 49.28 | 49.67 | 79.28 |
| 49 | 1.79 | 0.39 | 12.88 | 3.19E-07 | -4.27 | 12 | 0.0072 | 15.77 | 41.54 | 43.96 | 51.54 |
| 52 | 1.99 | 0.19 | 18 | 3.34E-07 | -2.66 | 9 | 0.1425 | 8.43 | 26.86 | 27.84 | 46.86 |
| 55 | 2.27 | 0.5 | 14.66 | 1.15E-07 | -10.05 | 9 | 0.0126 | 20.02 | 50.03 | 51.02 | 70.03 |
| 57 | 2.23 | 0.32 | 14 | 1.23E-07 | -6.91 | 12 | 0.0333 | 21.62 | 53.24 | 55.66 | 63.24 |
| 58 | 2.55 | 0.37 | 6.03 | 3.78E-08 | -4 | 8 | 0.019 | 13.03 | 36.07 | 36.47 | 66.07 |
| 59 | 1.72 | 0.36 | 7.12 | 2.22E-07 | -4.64 | 10 | 0.0343 | 16.2 | 42.4 | 43.91 | 57.4 |
| 61 | 0.68 | 0.43 | 4.29 | 1.40E-06 | -3.33 | 11 | 0.0767 | 7.86 | 25.72 | 27.71 | 37.72 |
| 62 | 2.38 | 0.6 | 9.55 | 8.22E-08 | -4.29 | 9 | 0.0855 | 15.65 | 41.3 | 42.28 | 61.3 |
| 64 | 1.43 | 0.16 | 19.09 | 4.56E-07 | -17.54 | 13 | 0.0429 | 27.27 | 64.54 | 67.36 | 73.11 |
| 65 | 2.45 | 0.31 | 21.41 | 5.06E-08 | -37.98 | 8 | 0.2838 | 21.06 | 52.12 | 52.52 | 82.12 |
| 67 | 2.16 | 0.33 | 6.46 | 9.32E-08 | -4.67 | 10 | 0.0429 | 10.67 | 31.34 | 32.85 | 46.34 |
| 71 | 2.71 | 0.44 | 2.3 | 1.05E-08 | -8.67 | 11 | 0.0313 | 25.06 | 60.12 | 62.11 | 72.12 |
| 73 | 2.61 | 0.48 | 5.02 | 3.12E-08 | -3.25 | 10 | 0.0753 | 11.72 | 33.43 | 34.95 | 48.43 |
| Mean | 1.994 | 0.673 | 9.885 | 0 | -8.468 | 9.605 | 0.079 | 17.08 | 44.161 | 45.417 | 63.908 |
| Median | 2.27 | 0.46 | 9.84 | 0 | -5.87 | 9 | 0.066 | 17.06 | 44.11 | 45.57 | 64.59 |
| IQR | 1.095 | 0.325 | 10.715 | 0 | 5.89 | 1.5 | 0.066 | 6.295 | 12.585 | 12.58 | 15.15 |
