# Supplementary material for: Modeling dynamics of acute HIV infection incorporating density-dependent cell death and multiplicity of infection
Source: PLoS Comput Biol. 2024 Jun 7;20(6):e1012129. doi: 10.1371/journal.pcbi.1012129 (PMC11189221; doi:10.1371/journal.pcbi.1012129)
Supplement: S3 Table — Parameter value estimates for the Density-Dependent Cell Death model, along with the negative log likelihood (nll), AIC, BIC and AICc. We also report mean, median and interquartile range (IQR) for the reader reference. (DOCX) [file pcbi.1012129.s005.docx]

Table S3: Parameter value estimates for the Density-Dependent Cell Death model, along with the negative log likelihood (NLL), AIC, BIC and AICc. We also report mean, median and interquartile range (IQR) for the reader reference.

| **ID** | **Log_10_(p)** | **delta** | **c** | **k** | **γ** | **t_0_** | **error value** | **NLL** | **AIC** | **BIC** | **AICc** |
| --- | --- | --- | --- | --- | --- | --- | --- | --- | --- | --- | --- |
| 1 | 3.74 | 5 | 4.03 | 4.93E-09 | 0.01 | -12.63 | 0.0811 | 26.74 | 65.49 | 67.87 | 86.49 |
| 2 | 2.38 | 1.44 | 23.26 | 3.056E-07 | 0.05 | -4.64 | 0.0514 | 14.54 | 41.07 | 42.26 | 83.07 |
| 4 | 2.08 | 1.21 | 23 | 4.22E-07 | 0.03 | -1.42 | 0.0916 | 4.13 | 20.26 | 22.08 | 48.26 |
| 5 | 4.34 | 2.38 | 22.89 | 3.79E-09 | 0.02 | -19.06 | 0.1144 | 15.77 | 43.54 | 44.01 | 127.54 |
| 6 | 3.28 | 1.88 | 9.83 | 1.62E-08 | 0.02 | -9.82 | 0.0484 | 18.03 | 48.07 | 48.55 | 132.07 |
| 7 | 2.85 | 1.03 | 8.31 | 2.17E-08 | 0.01 | -18 | 0.0259 | 27.03 | 66.06 | 68.97 | 82.86 |
| 8 | 3.57 | 2.58 | 10.37 | 1.29E-08 | 0.04 | -2.39 | 0.0286 | 11.18 | 34.36 | 35.54 | 76.36 |
| 11 | 3.91 | 1.89 | 5.44 | 2.79E-09 | 0.04 | -1.79 | 0.0289 | 9.98 | 31.95 | 33.13 | 73.95 |
| 12 | 4.1 | 4.42 | 3.35 | 2.27E-09 | 0.03 | -1.84 | 0.0629 | 12.75 | 37.5 | 39.32 | 65.5 |
| 20 | 3.44 | 3.09 | 22.91 | 4.05E-08 | 0.02 | -2.77 | 0.1563 | 10.63 | 33.26 | 33.74 | 117.26 |
| 21 | 3.15 | 1.2 | 1.98 | 6.19E-09 | 0.05 | -3.97 | 0.0596 | 15.73 | 43.46 | 44.64 | 85.46 |
| 22 | 3.03 | 3.13 | 13.9 | 6.75E-08 | 0.03 | -4.01 | 0.0449 | 17.59 | 47.17 | 48.99 | 75.17 |
| 23 | 3.18 | 3.99 | 22.91 | 9.15E-08 | 0.03 | -0.46 | 0.1063 | 7.12 | 26.23 | 27.42 | 68.23 |
| 24 | 3.28 | 3.39 | 23.02 | 4.65E-08 | 0 | -11.23 | 0.0603 | 14.4 | 40.81 | 40.48 | Inf |
| 25 | 2.29 | 0.61 | 14.93 | 1.36E-07 | 0 | -5.97 | 0.0378 | 16.52 | 45.03 | 46.22 | 87.03 |
| 26 | 3.34 | 1.32 | 10.27 | 1.49E-08 | 0.05 | -3.62 | 0.0397 | 12.62 | 37.25 | 37.72 | 121.25 |
| 27 | 3.75 | 2.63 | 3.51 | 3.27E-09 | 0.02 | -2.99 | 0.1286 | 15.85 | 43.7 | 44.88 | 85.7 |
| 28 | 3.31 | 1.94 | 13.09 | 1.93E-08 | 0.01 | -8.42 | 0.0465 | 21.74 | 55.48 | 57.29 | 83.48 |
| 29 | 3.57 | 3.18 | 10.71 | 1.39E-08 | 0.03 | -6.75 | 0.0791 | 16.13 | 44.25 | 44.73 | 128.25 |
| 31 | 2.55 | 5 | 11.59 | 1.96E-07 | 0 | -13.45 | 0.2452 | 21.84 | 55.68 | 56.86 | 97.68 |
| 32 | 1.28 | 1.66 | 12.43 | 1.92E-06 | 0.04 | -5.57 | 0.0159 | 12.86 | 37.72 | 38.19 | 121.72 |
| 33 | 1.55 | 0.94 | 23.04 | 1.29E-06 | 0 | -6.09 | 0.0361 | 16.34 | 44.68 | 45.86 | 86.68 |
| 34 | 2.92 | 0.76 | 9.47 | 2.62E-08 | 0.03 | -4.16 | 0.0149 | 15.78 | 43.55 | 45.94 | 64.55 |
| 37 | 2.43 | 1.08 | 43.1 | 2.45E-07 | 0.01 | -19.89 | 0.0551 | 26.71 | 65.41 | 68.8 | 79.41 |
| 40 | 3.96 | 4.99 | 10.24 | 7.62E-09 | 0.01 | -9.51 | 0.1516 | 22.06 | 56.13 | 57.31 | 98.13 |
| 41 | 3.92 | 4.64 | 5.06 | 4.46E-09 | 0.02 | -4.04 | 0.1175 | 17.39 | 46.79 | 47.97 | 88.79 |
| 42 | 2.46 | 0.17 | 4.49 | 2.74E-08 | 0.06 | -4.79 | 0.0074 | 16.85 | 45.7 | 47.52 | 73.7 |
| 44 | 2.63 | 1.83 | 22.91 | 1.88E-07 | 0.04 | -0.17 | 0.0743 | 6.65 | 25.29 | 27.68 | 46.29 |
| 46 | 3.35 | 1.82 | 22.99 | 2.71E-08 | 0.02 | -10.45 | 0.0472 | 22.83 | 57.65 | 60.04 | 78.65 |
| 48 | 3.45 | 0.76 | 2 | 3.06E-09 | 0.09 | -6.08 | 0.0765 | 17.6 | 47.19 | 47.67 | 131.19 |
| 49 | 2.14 | 0.34 | 22.9 | 2.45E-07 | 0.03 | -3.97 | 0.0065 | 15.1 | 42.2 | 45.11 | 59 |
| 52 | 1.96 | 0.02 | 9.51 | 1.89E-07 | 0.2 | -2.34 | 0.1299 | 8.2 | 28.4 | 29.59 | 70.4 |
| 55 | 3.3 | 2.02 | 12.52 | 2.23E-08 | 0.03 | -5.55 | 0.0043 | 17.54 | 47.08 | 48.26 | 89.08 |
| 57 | 3.7 | 1.89 | 18.62 | 1.27E-08 | 0.04 | -2.93 | 0.0161 | 18.5 | 49.01 | 51.92 | 65.81 |
| 58 | 1.93 | 0.49 | 0.64 | 4.30E-08 | 0.04 | -4.58 | 0.0083 | 14.37 | 40.74 | 41.21 | 124.74 |
| 59 | 3.24 | 1.6 | 15.57 | 2.9E-08 | 0.05 | -2.1 | 0.0185 | 12.26 | 36.51 | 38.33 | 64.51 |
| 61 | 2.48 | 1.33 | 32.34 | 2.7E-07 | 0.03 | -0.15 | 0.0545 | 2.5 | 17.01 | 19.4 | 38.01 |
| 62 | 3.61 | 3.55 | 8.82 | 1.27E-08 | 0.03 | -1.27 | 0.0498 | 12.56 | 37.13 | 38.31 | 79.13 |
| 64 | 2.45 | 1.8 | 3.87 | 4.32E-08 | 0.04 | -7.8 | 0.0357 | 21.79 | 55.57 | 58.96 | 69.57 |
| 65 | 2.64 | 2.17 | 4.71 | 3.92E-08 | 0.04 | -16.57 | 0.4188 | 18.41 | 48.83 | 49.31 | 132.83 |
| 67 | 3.46 | 0.72 | 15.62 | 1.44E-08 | 0.09 | -2.92 | 0.0203 | 9.98 | 31.95 | 33.77 | 59.95 |
| 71 | 3.99 | 3.19 | 2.2 | 1.74E-09 | 0.05 | -5.02 | 0.0139 | 21.89 | 55.78 | 58.17 | 76.78 |
| 73 | 2.58 | 0.55 | 1.2 | 1.57E-08 | 0.07 | -2.78 | 0.0209 | 11.92 | 35.83 | 37.65 | 63.83 |
| Mean | 3.037 | 2.084 | 13.106 | 0 | 0.036 | -6.139 | 0.068 | 15.591 | 43.181 | 44.69 | 85.437 |
| Median | 3.24 | 1.83 | 10.71 | 0 | 0.03 | -4.58 | 0.048 | 15.78 | 43.55 | 44.88 | 81.135 |
| IQR | 1.1 | 2.055 | 18.01 | 0 | 0.02 | 5.335 | 0.057 | 5.81 | 11.63 | 11.195 | 26.965 |
