# Supplementary material for: Modeling dynamics of acute HIV infection incorporating density-dependent cell death and multiplicity of infection
Source: PLoS Comput Biol. 2024 Jun 7;20(6):e1012129. doi: 10.1371/journal.pcbi.1012129 (PMC11189221; doi:10.1371/journal.pcbi.1012129)
Supplement: S4 Table — Parameter value estimates for the MOI model, along the negative log likelihood (nll), AIC, BIC and AICc. (DOCX) [file pcbi.1012129.s006.docx]

Table S4: Parameter value estimates for the MOI model, along the negative log likelihood (NLL), AIC, BIC and AICc. We also report mean, median and interquartile range (IQR) for the reader reference.

| **ID** | **Log_10_(λ)** | **α** | **η** | **k** | **β** | **t_0_** | **error value** | **NLL** | **AIC** | **BIC** | **AICc** |
| --- | --- | --- | --- | --- | --- | --- | --- | --- | --- | --- | --- |
| 1 | 2.87 | 0.61 | 12.04 | 6.4 | 2.14E-08 | -20.73 | 0.0642 | 29.89 | 71.78 | 74.17 | 92.78 |
| 2 | 0.4 | 0.11 | 11.52 | 4.08 | 5.55E-06 | -16.53 | 0.0491 | 22.71 | 57.42 | 58.6 | 99.42 |
| 4 | 1.26 | 0.24 | 42.97 | 13.63 | 2.36E-06 | -8.46 | 0.0557 | 19.9 | 51.8 | 53.61 | 79.8 |
| 5 | 0.82 | 0.06 | 15.4 | 1.01 | 2.24E-06 | -20.47 | 0.1244 | 17.32 | 46.64 | 47.12 | 130.64 |
| 6 | 1.84 | 0.78 | 1.44 | 1.88 | 5.64E-08 | -11.67 | 0.0621 | 19.71 | 51.41 | 51.89 | 135.41 |
| 7 | 2.48 | 0.72 | 6.95 | 2.04 | 3.55E-08 | -17.99 | 0.0217 | 28.84 | 69.68 | 72.59 | 86.48 |
| 8 | 3.4 | 1.3 | 24.87 | 0.32 | 2.65E-08 | -6.04 | 0.0451 | 11.23 | 34.47 | 35.65 | 76.47 |
| 11 | 3.41 | 0.95 | 3.65 | 0.9 | 4.55E-09 | -3.83 | 0.0612 | 14.21 | 40.41 | 41.6 | 82.41 |
| 12 | 3.64 | 1.12 | 7.8 | 0.64 | 5.38E-09 | -4.41 | 0.1621 | 17.89 | 47.77 | 49.59 | 75.77 |
| 20 | 2.48 | 2.08 | 8.77 | 0.5 | 8.36E-08 | -18.6 | 0.2516 | 19.89 | 51.77 | 52.25 | 135.77 |
| 21 | 2.42 | 0.95 | 0.85 | 4.05 | 1.57E-08 | -6.35 | 0.0706 | 18.94 | 49.87 | 51.06 | 91.87 |
| 22 | 1.83 | 0.51 | 17.86 | 1.99 | 4.57E-07 | -9.37 | 0.0982 | 23.69 | 59.38 | 61.19 | 87.38 |
| 23 | 0.72 | 0.21 | 7.22 | 1.72 | 2.25E-06 | -7.44 | 0.1212 | 18.87 | 49.74 | 50.92 | 91.74 |
| 24 | 2.43 | 3.32 | 4.77 | 3.59 | 7.19E-08 | -18.58 | 0.059 | 15.83 | 43.67 | 43.34 | Inf |
| 25 | 2.73 | 1.5 | 11.19 | 0.58 | 5.623E-08 | -8.4 | 0.04 | 19.41 | 50.83 | 52.01 | 92.83 |
| 26 | 1.81 | 0.2 | 5.31 | 13.57 | 1.09E-07 | -10.3 | 0.0271 | 18.28 | 48.55 | 49.03 | 132.55 |
| 27 | 2.58 | 0.83 | 1.34 | 10.75 | 1.13E-08 | -7.21 | 0.131 | 19.89 | 51.77 | 52.96 | 93.77 |
| 28 | 2.2 | 1.3 | 2.55 | 2.67 | 4.35E-08 | -12.34 | 0.0491 | 24.1 | 60.2 | 62.02 | 88.2 |
| 29 | 2.16 | 0.39 | 13.44 | 2.53 | 1.01E-07 | -20.19 | 0.096 | 21.73 | 55.46 | 55.94 | 139.46 |
| 31 | 2.29 | 3.89 | 9.82 | 0.25 | 2.72E-07 | -10.74 | 0.2508 | 21.49 | 54.98 | 56.17 | 96.98 |
| 32 | 0.38 | 0.41 | 14.09 | 2.01 | 1.08E-05 | -15.19 | 0.0132 | 19.04 | 50.08 | 50.56 | 134.08 |
| 33 | 1.58 | 1.12 | 16.78 | 3.92 | 1.01E-06 | -8.5 | 0.0351 | 19.91 | 51.82 | 53.01 | 93.82 |
| 34 | 3.48 | 0.84 | 38.13 | 1.56 | 2.73E-08 | -6.05 | 0.0194 | 21.02 | 54.03 | 56.42 | 75.03 |
| 37 | 2.86 | 5.01 | 11.59 | 0.07 | 9.25E-08 | -15.93 | 0.068 | 27.77 | 67.53 | 70.92 | 81.53 |
| 40 | 2.83 | 1.52 | 2.02 | 16.53 | 1.53E-08 | -6.59 | 0.0467 | 21.07 | 54.13 | 55.31 | 96.13 |
| 41 | 2.14 | 0.78 | 1.33 | 3.22 | 2.85E-08 | -8.7 | 0.1206 | 21.38 | 54.77 | 55.95 | 96.77 |
| 42 | 3.32 | 1.02 | 6.86 | 0.24 | 8.26-09 | -6.08 | 0.0093 | 20.54 | 53.07 | 54.89 | 81.07 |
| 44 | 0.82 | 0.21 | 10.13 | 2.13 | 2.14E-06 | -6.6 | 0.0774 | 21.22 | 54.45 | 56.83 | 75.45 |
| 46 | 1.44 | 0.72 | 1.89 | 0.7 | 1.11E-07 | -20.06 | 0.0606 | 27.43 | 66.87 | 69.25 | 87.87 |
| 48 | 2.66 | 0.33 | 2.01 | 6.04 | 9.46E-09 | -11.53 | 0.0526 | 20.78 | 53.56 | 54.04 | 137.56 |
| 49 | 2.77 | 1.61 | 14.92 | 0.26 | 7.15E-08 | -5.53 | 0.0087 | 21.53 | 55.05 | 57.96 | 71.85 |
| 52 | 3.1 | 2.29 | 9.15 | 0.06 | 3.19E-08 | -2.23 | 0.1409 | 11.79 | 35.59 | 36.77 | 77.59 |
| 55 | 2.48 | 0.53 | 18 | 2.64 | 8.47E-08 | -13.21 | 0.0074 | 22.92 | 57.85 | 59.03 | 99.85 |
| 57 | 2.65 | 0.66 | 11.68 | 0.56 | 4.65E-08 | -8.72 | 0.0277 | 26.6 | 65.2 | 68.11 | 82 |
| 58 | 2.08 | 0.62 | 1.11 | 0.46 | 2.98E-08 | -7.99 | 0.0239 | 17.81 | 47.61 | 48.09 | 131.61 |
| 59 | 2.35 | 0.56 | 14.94 | 0.62 | 1.15E-07 | -7.04 | 0.0299 | 21 | 54 | 55.81 | 82 |
| 61 | 1.68 | 1.01 | 12.14 | 0.55 | 5.24E-07 | -4.28 | 0.0713 | 16.83 | 45.66 | 48.04 | 66.66 |
| 62 | 2.58 | 0.61 | 11.54 | 4.15 | 5.79E-08 | -6.17 | 0.0763 | 19.76 | 51.53 | 52.71 | 93.53 |
| 64 | 2.55 | 0.98 | 17.05 | 0.15 | 7.19E-08 | -20.39 | 0.0386 | 32.05 | 76.09 | 79.48 | 90.09 |
| 65 | 2.75 | 0.31 | 39.55 | 4.66 | 4.69E-08 | -49.59 | 0.2866 | 23.38 | 58.75 | 59.23 | 142.75 |
| 67 | 2.57 | 1.52 | 2.76 | 0.16 | 3.03E-08 | -5.56 | 0.0288 | 14.05 | 40.09 | 41.91 | 68.09 |
| 71 | 2.94 | 0.87 | 1.48 | 0.56 | 5.64E-09 | -9.88 | 0.0263 | 26.3 | 64.59 | 66.98 | 85.59 |
| 73 | 2.66 | 0.48 | 2.28 | 5.49 | 1.53E-08 | -3.85 | 0.0322 | 12.48 | 36.95 | 38.77 | 64.95 |
| Mean | 2.289 | 1.048 | 10.958 | 3.02 | NA | -11.38 | 0.073 | 20.709 | 53.416 | 54.925 | 95.848 |
| Median | 2.48 | 0.78 | 9.82 | 1.88 | NA | -8.7 | 0.056 | 20.54 | 53.07 | 54.04 | 90.915 |
| IQR | 0.925 | 0.715 | 11.85 | 3.43 | NA | 9.3 | 0.057 | 4.24 | 8.49 | 8.74 | 17.625 |
