# Supplementary material for: Modeling dynamics of acute HIV infection incorporating density-dependent cell death and multiplicity of infection
Source: PLoS Comput Biol. 2024 Jun 7;20(6):e1012129. doi: 10.1371/journal.pcbi.1012129 (PMC11189221; doi:10.1371/journal.pcbi.1012129)
Supplement: S5 Table — Parameter value estimates for the Density-Dependent Cell Death & MOI model, along the negative log likelihood (nll), AIC, BIC and AICc. (DOCX) [file pcbi.1012129.s007.docx]

Table S5: Parameter value estimates for the Density-Dependent Cell Death & MOI model, along the negative log likelihood (NLL), AIC, BIC and AICc. We also report mean, median and interquartile range (IQR) for the reader reference.

| **ID** | **Log_10_(λ)** | **α** | **η** | **k** | **β** | **γ** | **t_0_** | **error value** | **NLL** | **AIC** | **BIC** | **AICc** |
| --- | --- | --- | --- | --- | --- | --- | --- | --- | --- | --- | --- | --- |
| 1 | 2.59 | 0.6 | 6.56 | 9.93 | 2.26E-08 | 1E-04 | -20.38 | 0.0638 | 29.56 | 73.12 | 75.9 | 110.45 |
| 2 | 0.33 | 0.1 | 9.92 | 48.46 | 5.88E-06 | 1E-04 | -16.5 | 0.037 | 22.73 | 59.46 | 60.85 | 171.46 |
| 4 | 0.9 | 0.24 | 19.08 | 24.91 | 2.55E-06 | 1E-04 | -7.55 | 0.0513 | 18.82 | 51.64 | 53.76 | 107.64 |
| 5 | 0 | 0.01 | 12.08 | 2.86 | 4.3E-05 | 6E-04 | -13.28 | 0.0547 | 18.11 | 50.21 | 50.77 | Inf |
| 6 | 3.79 | 4.32 | 11.81 | 3.57 | 9.49E-09 | 0.012 | -12.92 | 0.0607 | 19.42 | 52.83 | 53.39 | Inf |
| 7 | 2.83 | 0.88 | 12.01 | 6.15 | 2.74E-08 | 0.0109 | -19 | 0.0226 | 29.02 | 72.04 | 75.43 | 100.04 |
| 8 | 3.91 | 5.76 | 11.88 | 3.71 | 9.17E-09 | 0.0268 | -3.56 | 0.0284 | 10.46 | 34.92 | 36.3 | 146.92 |
| 11 | 3.11 | 0.56 | 12.03 | 3.18 | 1.27E-08 | 5E-04 | -14.85 | 0.249 | 22.42 | 58.85 | 60.23 | 170.85 |
| 12 | 2.81 | 0.45 | 7.6 | 2.77 | 1.75E-08 | 6E-04 | -11.96 | 0.187 | 24.84 | 63.68 | 65.8 | 119.68 |
| 20 | 1.74 | 0.45 | 11.92 | 7.74 | 3.2E-07 | 1E-04 | -10.44 | 0.1921 | 18.6 | 51.2 | 51.75 | Inf |
| 21 | 3.22 | 0.72 | 9.11 | 3.65 | 9.96E-09 | 0.0127 | -8.98 | 0.1 | 21.24 | 56.48 | 57.86 | 168.48 |
| 22 | 2.96 | 4.31 | 11.59 | 6.99 | 6.645E-08 | 0.0266 | -5.5 | 0.0449 | 19.37 | 52.73 | 54.85 | 108.73 |
| 23 | 0.49 | 0.21 | 2.35 | 54.72 | 1.97E-06 | 1E-04 | -6.58 | 0.0701 | 18.01 | 50.01 | 51.39 | 162.01 |
| 24 | 2.87 | 2.99 | 11.9 | 1.32 | 5.08E-08 | 0.0177 | -10.77 | 0.0822 | 12.95 | 39.9 | 39.52 | -72.1 |
| 25 | 2.47 | 0.91 | 12.3 | 1.47 | 8.43E-08 | 1E-04 | -8.9 | 0.0417 | 19.89 | 53.78 | 55.16 | 165.78 |
| 26 | 2.18 | 0.21 | 11.53 | 23.92 | 9.52E-08 | 1E-04 | -10.4 | 0.0265 | 18.59 | 51.19 | 51.74 | Inf |
| 27 | 4.23 | 3.16 | 9.95 | 8.63 | 2.46E-09 | 0.0178 | -5.39 | 0.1364 | 17.76 | 49.52 | 50.9 | 161.52 |
| 28 | 2.71 | 0.92 | 12 | 20 | 4.34E-08 | 1E-04 | -12.03 | 0.06 | 24.63 | 63.26 | 65.37 | 119.26 |
| 29 | 2.08 | 0.35 | 12.04 | 3.34 | 1.08E-07 | 0.0079 | -17.69 | 0.0932 | 21.33 | 56.66 | 57.21 | Inf |
| 31 | 3.46 | 17.99 | 11.18 | 7.26 | 7.8E-08 | 0.0095 | -5.26 | 0.1416 | 17.18 | 48.37 | 49.75 | 160.37 |
| 32 | 0.13 | 0.37 | 7.77 | 89.85 | 1.52E-05 | 0.0361 | -15.12 | 0.0091 | 18.94 | 51.89 | 52.45 | Inf |
| 33 | 1.39 | 1.02 | 12.07 | 12.46 | 1.11E-06 | 1E-04 | -8.31 | 0.0409 | 19.75 | 53.51 | 54.89 | 165.51 |
| 34 | 2.68 | 0.95 | 9.11 | 8.93 | 3.40E-08 | 0.0176 | -11.98 | 0.0515 | 24.74 | 63.49 | 66.27 | 100.82 |
| 37 | 1.55 | 1.67 | 12.1 | 0.03 | 1.57E-06 | 0.001 | -2.25 | 0.2932 | 14.8 | 43.61 | 47.56 | 66.01 |
| 40 | 4.06 | 9.46 | 3.97 | 10.61 | 4.74E-09 | 0.011 | -5.72 | 0.0448 | 19.78 | 53.57 | 54.95 | 165.57 |
| 41 | 2.94 | 2.1 | 2.37 | 9.41 | 1.048E-08 | 0.0329 | -6.22 | 0.1143 | 19.26 | 52.53 | 53.91 | 164.53 |
| 42 | 3.61 | 1.18 | 12.06 | 0.35 | 6.67E-09 | 0.0313 | -5.13 | 0.0078 | 19.37 | 52.74 | 54.86 | 108.74 |
| 44 | 2.02 | 2.1 | 11.77 | 7.09 | 2.9E-07 | 0.067 | -2.72 | 0.0713 | 13.7 | 41.4 | 44.19 | 78.73 |
| 46 | 3.39 | 3.67 | 11.92 | 2.08 | 1.97E-08 | 0.0169 | -11.03 | 0.0477 | 23.72 | 61.44 | 64.22 | 98.77 |
| 48 | 5.06 | 6.2 | 11.44 | 8.48 | 7.08E-10 | 0.027 | -5.91 | 0.0741 | 17.07 | 48.15 | 48.7 | Inf |
| 49 | 2.6 | 1.58 | 11.97 | 1.14 | 7.17E-08 | 0.0302 | -4.63 | 0.0141 | 19.71 | 53.41 | 56.81 | 81.41 |
| 52 | 3.01 | 0.97 | 12 | 0.99 | 1.95E-08 | 5E-04 | -14.99 | 0.7707 | 21.74 | 57.48 | 58.86 | 169.48 |
| 55 | 2.8 | 1.37 | 11.99 | 1.36 | 4.09E-08 | 0.0254 | -10.11 | 0.0066 | 21.06 | 56.12 | 57.5 | 168.12 |
| 57 | 3.71 | 3.96 | 12.13 | 4.39 | 1.05E-08 | 0.0315 | -4.67 | 0.0161 | 21.11 | 56.23 | 59.62 | 84.23 |
| 58 | 2.91 | 0.31 | 11.96 | 1.67 | 2.36E-08 | 1E-04 | -7.74 | 0.0213 | 18.4 | 50.81 | 51.36 | Inf |
| 59 | 3.11 | 2.76 | 11.94 | 3.25 | 2.99E-08 | 0.0478 | -3.79 | 0.018 | 15.93 | 45.86 | 47.98 | 101.86 |
| 61 | 1.97 | 1.78 | 11.95 | 1.4 | 3.21E-07 | 0.0274 | -2.76 | 0.0542 | 12.78 | 39.55 | 42.34 | 76.89 |
| 62 | 2.98 | 1.04 | 12 | 1 | 2.6E-08 | 6E-04 | -9.97 | 0.2945 | 21.89 | 57.78 | 59.16 | 169.78 |
| 64 | 3.06 | 3.01 | 11.91 | 1.09 | 3.08E-08 | 0.0329 | -9.14 | 0.036 | 24.47 | 62.95 | 66.9 | 85.35 |
| 65 | 1.98 | 0.78 | 12.03 | 0.08 | 3.64E-07 | 7E-04 | -5.52 | 0.4364 | 16.86 | 47.72 | 48.27 | Inf |
| 67 | 3.55 | 2.79 | 11.99 | 2.89 | 1.1E-08 | 0.0692 | -4.16 | 0.0201 | 10.12 | 34.24 | 36.36 | 90.24 |
| 71 | 4.61 | 3.14 | 11.9 | 3.4 | 1.1E-09 | 0.0338 | -7.14 | 0.016 | 24.22 | 62.45 | 65.23 | 99.78 |
| 73 | 2.44 | 0.46 | 4.66 | 2.82 | 3.11E-08 | 7E-04 | -7.58 | 0.1742 | 17.94 | 49.89 | 52 | 105.89 |
| Mean | 2.657 | 2.275 | 10.741 | 9.752 | 0 | 0.016 | -9.036 | 0.102 | 19.588 | 53.178 | 54.938 | 120.082 |
| Median | 2.83 | 1.04 | 11.92 | 3.57 | 0 | 0.011 | -8.31 | 0.054 | 19.37 | 52.74 | 54.85 | 109.595 |
| IQR | 1.255 | 2.49 | 1.44 | 7.21 | 0 | 0.027 | 6.525 | 0.08 | 3.965 | 7.925 | 8.555 | 66.242 |
