# Supplementary material for: Modeling dynamics of acute HIV infection incorporating density-dependent cell death and multiplicity of infection
Source: PLoS Comput Biol. 2024 Jun 7;20(6):e1012129. doi: 10.1371/journal.pcbi.1012129 (PMC11189221; doi:10.1371/journal.pcbi.1012129)
Supplement: S6 Table — Summary of which model is selected by AIC, BIC and AICc for each study participant. (DOCX) [file pcbi.1012129.s008.docx]

Table S6: Summary of which model is selected by AIC, BIC and AICc for each study participant.

| **ID** | **AIC** | **BIC** | **AICc** | **ID** | **AIC** | **BIC** | **AICc** |  |
| --- | --- | --- | --- | --- | --- | --- | --- | --- |
| 1 | Standard | Standard | Standard | 34 | DDDI | DDDI | Standard |  |
| 2 | DDDI | DDDI | Standard | 37 | DDDDI & MOI | DDDDI & MOI | DDDDI & MOI |  |
| 4 | DDDI | DDDI | Standard | 40 | Standard | Standard | Standard |  |
| 5 | Standard | Standard | Standard | 41 | DDDI | DDDI | Standard |  |
| 6 | DDDI | DDDI | Standard | 42 | Standard | Standard | Standard |  |
| 7 | Standard | Standard | Standard | 44 | DDDI | DDDI | Standard |  |
| 8 | Standard | Standard | Standard | 46 | DDDI | DDDI | Standard |  |
| 11 | DDDI | DDDI | Standard | 48 | DDDI | DDDI | Standard |  |
| 12 | DDDI | DDDI | Standard | 49 | Standard | Standard | Standard |  |
| 20 | DDDI | DDDI | Standard | 52 | Standard | Standard | Standard |  |
| 21 | DDDI | DDDI | Standard | 55 | DDDI | DDDI | Standard |  |
| 22 | Standard | Standard | Standard | 57 | DDDI | DDDI | Standard |  |
| 23 | DDDI | DDDI | Standard | 58 | Standard | Standard | Standard |  |
| 24 | Standard | Standard | DDDDI & MOI | 59 | DDDI | DDDI | Standard |  |
| 25 | Standard | Standard | Standard | 61 | DDDI | DDDI | Standard |  |
| 26 | DDDI | DDDI | Standard | 62 | DDDI | DDDI | Standard |  |
| 27 | DDDI | DDDI | Standard | 64 | DDDI | DDDI | DDDI |  |
| 28 | Standard | Standard | Standard | 65 | Standard | Standard | Standard |  |
| 29 | DDDI | DDDI | Standard | 67 | DDDI | DDDI | Standard |  |
| 31 | Standard | Standard | Standard | 71 | Standard | Standard | Standard |  |
| 32 | DDDI | DDDI | Standard | 73 | Standard | Standard | Standard |  |
| 33 | Standard | Standard | Standard |  |  |  |  |  |
