# Supplementary material for: Modeling dynamics of acute HIV infection incorporating density-dependent cell death and multiplicity of infection
Source: PLoS Comput Biol. 2024 Jun 7;20(6):e1012129. doi: 10.1371/journal.pcbi.1012129 (PMC11189221; doi:10.1371/journal.pcbi.1012129)
Supplement: S7 Table — Sum of negative log likelihood (NLL), AIC, BIC, AICc across all study participants (n = 43). (DOCX) [file pcbi.1012129.s009.docx]

Table S7: We report the sum of negative log likelihood (NLL) across all study participants (n=43), as well as the number of parameters estimated and AIC, BIC, AICc across all study participants.

| **Model** | **NLL** | **# Parameters** | **AIC** | **BIC** | **AICc** |
| --- | --- | --- | --- | --- | --- |
| Standard | 774.88 | 215 | 1979.76 | 2358.42 | 1442.88 |
| Density-Dependent Cell Death | 707.6 | 258 | 1931.2 | 2385.59 | 1312.47 |
| MOI | 935.95 | 258 | 2387.9 | 2842.29 | 1769.18 |
| Density-Dependent Cell Death & MOI | 885.21 | 301 | 2372.42 | 2902.54 | 1670 |
