# Supplementary material for: Modeling dynamics of acute HIV infection incorporating density-dependent cell death and multiplicity of infection
Source: PLoS Comput Biol. 2024 Jun 7;20(6):e1012129. doi: 10.1371/journal.pcbi.1012129 (PMC11189221; doi:10.1371/journal.pcbi.1012129)
Supplement: S8 Table — Data-derived growth rate and model-derived growth rates for each study participants, along with the squared difference for the data-and-model derived rates for each model. (DOCX) [file pcbi.1012129.s010.docx]

Table S8: Data-derived growth rate and model-derived growth rates for each study participants, along with the squared difference for the data-and-model derived rates for each model. We also report mean, median and interquartile range (IQR) for the reader reference.

| **ID** | **Data Growth Rate** | **Standard**  **Growth Rate** | **Error Standard** | **DDDI Growth Rate** | **Error DDDI** | **MOI Growth Rate** | **Error MOI** | **DDDDI & MOI Growth Rate** | **Error DDDDI & MOI** | **Best Model** |
| --- | --- | --- | --- | --- | --- | --- | --- | --- | --- | --- |
| 1 | 0.25 | 0.29 | 0.00147 | 0.29 | 0.0012 | 0.27 | 0.00037 | 0.27 | 0.00018 | DDDDI & MOI |
| 2 | 0.32 | 0.45 | 0.0162 | 0.44 | 0.01329 | 0.29 | 0.00129 | 0.29 | 0.00138 | DDDI |
| 4 | 0.28 | 0.31 | 0.00116 | 0.3 | 0.00075 | 0.29 | 0.00022 | 0.29 | 0.8E-4 | DDDDI & MOI |
| 5 | 0.19 | 1.08 | 0.78659 | 0.41 | 0.04914 | 0.32 | 0.01715 | 0.32 | 0.01746 | DDDI |
| 6 | 0.24 | 0.35 | 0.01297 | 0.37 | 0.01745 | 0.37 | 0.01892 | 0.34 | 0.01109 | DDDDI & MOI |
| 7 | 0.19 | 0.32 | 0.01845 | 0.26 | 0.00574 | 0.29 | 0.01108 | 0.29 | 0.00983 | MOI |
| 8 | 0.52 | 0.54 | 0.00035 | 0.49 | 0.00075 | 0.53 | 1E-04 | 0.49 | 0.00083 | DDDI |
| 11 | 0.35 | 0.53 | 0.03308 | 0.54 | 0.03493 | 0.59 | 0.0562 | 0.31 | 0.00195 | DDDDI & MOI |
| 12 | 0.31 | 0.53 | 0.04923 | 0.57 | 0.06657 | 0.62 | 0.09704 | 0.38 | 0.00428 | DDDDI & MOI |
| 20 | 0.17 | 0.54 | 0.1396 | 0.51 | 0.11865 | 0.25 | 0.00734 | 0.39 | 0.04766 | DDDI |
| 21 | 0.43 | 0.54 | 0.01167 | 0.53 | 0.00987 | 0.48 | 0.00291 | 0.43 | 0.3E-4 | DDDDI & MOI |
| 22 | 0.32 | 0.66 | 0.11254 | 0.53 | 0.04211 | 0.46 | 0.02031 | 0.53 | 0.04525 | DDDI |
| 23 | 0.38 | 0.62 | 0.05572 | 0.53 | 0.02196 | 0.4 | 0.00034 | 0.4 | 0.00057 | DDDI |
| 24 | 0.14 | 0.16 | 0.2E-3 | 0.16 | 0.00036 | 0.16 | 0.00023 | 0.21 | 0.004 | Standard |
| 25 | 0.48 | 0.45 | 0.00074 | 0.45 | 0.00072 | 0.42 | 0.00331 | 0.41 | 0.00421 | MOI |
| 26 | 0.4 | 0.41 | 0.4E-4 | 0.48 | 0.00578 | 0.39 | 0.00017 | 0.41 | 1.06E-6 | DDDDI & MOI |
| 27 | 0.37 | 0.48 | 0.01265 | 0.49 | 0.01524 | 0.43 | 0.00415 | 0.49 | 0.01508 | DDDI |
| 28 | 0.26 | 0.34 | 0.00537 | 0.35 | 0.0081 | 0.32 | 0.00354 | 0.35 | 0.00758 | DDDI |
| 29 | 0.19 | 0.33 | 0.01896 | 0.43 | 0.06048 | 0.27 | 0.0069 | 0.3 | 0.01169 | DDDI |
| 31 | 0.13 | 0.59 | 0.21668 | 0.28 | 0.02266 | 0.4 | 0.0751 | 0.56 | 0.18672 | MOI |
| 32 | 0.21 | 0.22 | 2E-04 | 0.29 | 0.00765 | 0.26 | 0.00257 | 0.25 | 0.00198 | Standard |
| 33 | 0.43 | 0.4 | 0.00086 | 0.42 | 0.00028 | 0.4 | 0.00129 | 0.39 | 0.00161 | MOI |
| 34 | 0.46 | 0.53 | 0.00505 | 0.53 | 0.0048 | 0.55 | 0.00836 | 0.36 | 0.00937 | MOI |
| 37 | 0.11 | 0.17 | 0.00339 | 0.16 | 0.00263 | 0.21 | 0.01003 | 0.83 | 0.51031 | MOI |
| 40 | 0.48 | 0.63 | 0.02104 | 0.45 | 0.00086 | 0.62 | 0.02002 | 0.64 | 0.02427 | MOI |
| 41 | 0.32 | 0.49 | 0.02946 | 0.47 | 0.02329 | 0.41 | 0.00754 | 0.47 | 0.02246 | DDDI |
| 42 | 0.46 | 0.51 | 0.00229 | 0.51 | 0.00227 | 0.48 | 0.00045 | 0.52 | 0.00294 | DDDI |
| 44 | 0.33 | 0.48 | 0.02197 | 0.43 | 0.0094 | 0.38 | 0.0018 | 0.45 | 0.01241 | DDDI |
| 46 | 0.16 | 0.23 | 0.00556 | 0.27 | 0.01344 | 0.21 | 0.00314 | 0.26 | 0.01064 | DDDI |
| 48 | 0.34 | 0.52 | 0.03033 | 0.62 | 0.07535 | 0.46 | 0.01402 | 0.65 | 0.09556 | DDDI |
| 49 | 0.43 | 0.44 | 0.00033 | 0.44 | 0.2E-4 | 0.43 | 0.32E-4 | 0.42 | 0.1E-4 | DDDDI & MOI |
| 52 | 0.29 | 0.65 | 0.12639 | 0.64 | 0.1222 | 0.64 | 0.12419 | 0.26 | 0.00077 | DDDDI & MOI |
| 55 | 0.34 | 0.37 | 0.00127 | 0.41 | 0.00487 | 0.36 | 0.00042 | 0.39 | 0.00307 | DDDI |
| 57 | 0.39 | 0.47 | 0.00509 | 0.44 | 0.00185 | 0.42 | 0.00049 | 0.45 | 0.00261 | DDDI |
| 58 | 0.45 | 0.62 | 0.03128 | 0.56 | 0.01339 | 0.44 | 4.23E-9 | 0.49 | 0.00228 | DDDI |
| 59 | 0.36 | 0.47 | 0.01031 | 0.46 | 0.00997 | 0.44 | 0.00619 | 0.47 | 0.01022 | DDDI |
| 61 | 0.28 | 0.38 | 0.00944 | 0.36 | 0.00653 | 0.36 | 0.00649 | 0.37 | 0.00805 | DDDI |
| 62 | 0.54 | 0.53 | 0.00016 | 0.54 | 0.2E-4 | 0.49 | 0.00334 | 0.38 | 0.02616 | MOI |
| 64 | 0.16 | 0.2 | 0.00119 | 0.22 | 0.00369 | 0.2 | 0.00112 | 0.24 | 0.00576 | DDDI |
| 65 | 0.32 | 0.15 | 0.02861 | 0.24 | 0.00573 | 0.14 | 0.03127 | 0.73 | 0.16905 | MOI |
| 67 | 0.55 | 0.61 | 0.00278 | 0.57 | 0.00022 | 0.55 | 0.9E-4 | 0.61 | 0.00252 | DDDI |
| 71 | 0.44 | 0.49 | 0.00253 | 0.51 | 0.00439 | 0.46 | 0.00044 | 0.52 | 0.00575 | DDDI |
| 73 | 0.6 | 0.65 | 0.00188 | 0.64 | 0.0012 | 0.61 | 0.3E-4 | 0.45 | 0.0239 | DDDI |
| Mean | 0.334 | 0.459 | 0.043 | 0.432 | 0.019 | 0.397 | 0.013 | 0.42 | 0.031 | NA |
| Median | 0.33 | 0.48 | 0.009 | 0.45 | 0.007 | 0.4 | 0.003 | 0.4 | 0.006 | NA |
| IQR | 0.185 | 0.195 | 0.028 | 0.175 | 0.018 | 0.18 | 0.01 | 0.175 | 0.014 | NA |
