# Supplementary material for: Modeling dynamics of acute HIV infection incorporating density-dependent cell death and multiplicity of infection
Source: PLoS Comput Biol. 2024 Jun 7;20(6):e1012129. doi: 10.1371/journal.pcbi.1012129 (PMC11189221; doi:10.1371/journal.pcbi.1012129)
Supplement: S9 Table — Data-derived decay rate and model-derived decay rates for each study participants, along with the squared difference for the data-and-model derived rates for each model. (DOCX) [file pcbi.1012129.s011.docx]

Table S9: Data-derived decay rate and model-derived decay rates for each study participants, along with the squared difference for the data-and-model derived rates for each model. We also report mean, median and interquartile range (IQR) for the reader reference.

| **ID** | **Data Decay Rate** | **Standard Decay Rate** | **Error Standard** | **DDDI**  **Decay Rate** | **Error DDDI** | **MOI**  **Decay Rate** | **Error MOI** | **DDDDI & MOI Decay Rate** | **Error DDDDI & MOI** | **Best Model** |
| --- | --- | --- | --- | --- | --- | --- | --- | --- | --- | --- |
| 1 | -0.13 | -0.13 | 1.36E-6 | -0.17 | 0.00166 | -0.15 | 0.00025 | -0.15 | 0.00026 | Standard |
| 2 | -0.14 | -0.11 | 0.00069 | -0.15 | 0.00028 | -0.15 | 0.00028 | -0.2 | 0.00438 | MOI |
| 4 | -0.21 | -0.1 | 0.01223 | -0.14 | 0.00591 | -0.15 | 0.00423 | -0.17 | 0.00154 | DDDDI & MOI |
| 5 | -0.19 | -0.16 | 0.00099 | -0.17 | 0.00037 | -0.13 | 0.00425 | -0.22 | 6E-04 | MOI |
| 6 | -0.24 | -0.15 | 0.00664 | -0.17 | 0.00388 | -0.2 | 0.00136 | -0.17 | 0.00484 | DDDI |
| 7 | -0.11 | -0.14 | 0.00093 | -0.14 | 0.00136 | -0.15 | 0.00172 | -0.15 | 0.00167 | Standard |
| 8 | -0.15 | -0.13 | 0.00036 | -0.18 | 0.00082 | -0.17 | 0.00027 | -0.17 | 0.00033 | DDDI |
| 11 | -0.18 | -0.23 | 0.0026 | -0.24 | 0.00289 | -0.27 | 0.00723 | -0.16 | 0.00049 | DDDDI & MOI |
| 12 | -0.24 | -0.25 | 0.00013 | -0.25 | 7E-05 | -0.25 | 0.00028 | -0.19 | 0.00256 | MOI |
| 20 | -0.35 | -0.17 | 0.02955 | -0.23 | 0.01363 | -0.14 | 0.04168 | -0.2 | 0.02206 | MOI |
| 21 | -0.26 | -0.24 | 0.00043 | -0.23 | 0.00105 | -0.25 | 0.00017 | -0.22 | 0.00219 | DDDI |
| 22 | -0.18 | -0.16 | 4E-04 | -0.24 | 0.00285 | -0.2 | 0.00016 | -0.23 | 0.0025 | DDDI |
| 23 | -0.21 | -0.13 | 0.00649 | -0.18 | 8E-04 | -0.2 | 0.00012 | -0.35 | 0.01963 | DDDI |
| 24 | NA | NA | NA | NA | NA | NA | NA | NA | NA | NA |
| 25 | -0.15 | -0.18 | 0.00101 | -0.18 | 0.00107 | -0.18 | 0.00136 | -0.19 | 0.00168 | Standard |
| 26 | -0.19 | -0.12 | 0.00582 | -0.16 | 0.00092 | -0.25 | 0.00323 | -0.25 | 0.00334 | MOI |
| 27 | -0.3 | -0.25 | 0.00292 | -0.25 | 0.00229 | -0.28 | 0.00034 | -0.25 | 0.00301 | DDDI |
| 28 | -0.22 | -0.19 | 0.00055 | -0.22 | 1E-05 | -0.21 | 0.00012 | -0.2 | 4E-04 | MOI |
| 29 | -0.27 | -0.12 | 0.02153 | -0.15 | 0.01289 | -0.13 | 0.01745 | -0.14 | 0.01592 | MOI |
| 31 | -0.27 | -0.27 | 1.05E-7 | -0.19 | 0.00718 | -0.2 | 0.00484 | -0.31 | 0.00121 | Standard |
| 32 | -0.16 | -0.1 | 0.00394 | -0.12 | 0.00196 | -0.13 | 0.00094 | -0.16 | 1E-05 | DDDDI & MOI |
| 33 | -0.17 | -0.21 | 0.00189 | -0.21 | 0.00223 | -0.22 | 0.00291 | -0.23 | 0.0043 | Standard |
| 34 | -0.16 | -0.23 | 0.00398 | -0.22 | 0.0037 | -0.25 | 0.00691 | -0.18 | 0.00034 | DDDDI & MOI |
| 37 | -0.05 | -0.07 | 0.00036 | -0.08 | 0.00102 | -0.09 | 0.00154 | -0.05 | 1.55E-6 | DDDDI & MOI |
| 40 | -0.26 | -0.37 | 0.01159 | -0.3 | 0.00157 | -0.39 | 0.01608 | -0.37 | 0.01339 | MOI |
| 41 | -0.24 | -0.26 | 0.00031 | -0.25 | 1E-05 | -0.24 | 1.99E-6 | -0.25 | 0.00014 | DDDI |
| 42 | -0.11 | -0.1 | 0.00018 | -0.11 | 1.67E-6 | -0.14 | 0.00043 | -0.13 | 0.00026 | MOI |
| 44 | -0.16 | -0.09 | 0.00413 | -0.15 | 1E-04 | -0.19 | 0.001 | -0.14 | 0.00045 | MOI |
| 46 | -0.15 | -0.09 | 0.00306 | -0.11 | 0.00154 | -0.1 | 0.00189 | -0.12 | 0.00061 | DDDDI & MOI |
| 48 | -0.35 | -0.19 | 0.02578 | -0.23 | 0.01432 | -0.31 | 0.00173 | -0.24 | 0.01151 | DDDI |
| 49 | -0.11 | -0.12 | 0.00019 | -0.14 | 0.00064 | -0.14 | 0.00082 | -0.17 | 0.00326 | Standard |
| 52 | -0.07 | -0.06 | 9E-05 | -0.09 | 0.00038 | -0.08 | 2E-05 | -0.13 | 0.00356 | DDDI |
| 55 | -0.13 | -0.14 | 0.5E-4 | -0.18 | 0.00189 | -0.18 | 0.00246 | -0.16 | 0.00082 | Standard |
| 57 | -0.1 | -0.11 | 1E-04 | -0.16 | 0.00424 | -0.16 | 0.00334 | -0.16 | 0.00357 | Standard |
| 58 | -0.14 | -0.13 | 0.00024 | -0.16 | 0.00056 | -0.17 | 0.00089 | -0.22 | 0.00685 | Standard |
| 59 | -0.15 | -0.12 | 0.00098 | -0.16 | 5E-05 | -0.15 | 2.96E-6 | -0.15 | 3E-05 | DDDI |
| 61 | -0.26 | -0.13 | 0.01501 | -0.16 | 0.00815 | -0.14 | 0.01289 | -0.16 | 0.0096 | MOI |
| 62 | -0.18 | -0.2 | 0.00028 | -0.26 | 0.0058 | -0.25 | 0.00559 | -0.18 | 1E-05 | DDDDI & MOI |
| 64 | -0.05 | -0.05 | 1.6E-6 | -0.06 | 0.00022 | -0.05 | 6E-05 | -0.06 | 0.00017 | Standard |
| 65 | NA | NA | NA | NA | NA | NA | NA | NA | NA | NA |
| 67 | -0.11 | -0.12 | 2E-05 | -0.16 | 0.00219 | -0.14 | 0.00106 | -0.17 | 0.00295 | Standard |
| 71 | -0.14 | -0.16 | 0.00024 | -0.19 | 0.00194 | -0.21 | 0.00419 | -0.18 | 0.00141 | Standard |
| 73 | -0.18 | -0.18 | 2E-05 | -0.27 | 0.00801 | -0.42 | 0.05464 | -0.24 | 0.00388 | Standard |
| Mean | -0.181 | -0.158 | 0.004 | -0.181 | 0.003 | -0.19 | 0.005 | -0.19 | 0.004 | NA |
| Median | -0.17 | -0.14 | 0.001 | -0.17 | 0.002 | -0.18 | 0.001 | -0.18 | 0.002 | NA |
| IQR | 0.1 | 0.07 | 0.004 | 0.08 | 0.003 | 0.1 | 0.004 | 0.06 | 0.003 | NA |
