# Supplementary material for: Modeling dynamics of acute HIV infection incorporating density-dependent cell death and multiplicity of infection
Source: PLoS Comput Biol. 2024 Jun 7;20(6):e1012129. doi: 10.1371/journal.pcbi.1012129 (PMC11189221; doi:10.1371/journal.pcbi.1012129)
Supplement: S10 Table — Data-derived setpoint and model-derived setpoint for each study participants, along with the squared difference for the data-and-model derived setpoint for each model. (DOCX) [file pcbi.1012129.s012.docx]

Table S10: Data-derived setpoint and model-derived setpoint for each study participants, along with the squared difference for the data-and-model derived setpoint for each model. We also report mean, median and interquartile range (IQR) for the reader reference.

| **ID** | **Data Setpoint** | **Standard**  **Setpoint** | **Error Standard** | **DDDI**  **Setpoint** | **Error DDDI** | **MOI**  **Setpoint** | **Error MOI** | **DDDDI & MOI Setpoint** | **Error DDDDI & MOI** | **Model** |
| --- | --- | --- | --- | --- | --- | --- | --- | --- | --- | --- |
| 1 | 3.9 | 5.69 | 3.18 | 5.75 | 3.4 | 5.72 | 3.28 | 5.72 | 3.3 | Standard |
| 2 | 3.69 | 4.14 | 0.2 | 4.21 | 0.26 | 4.17 | 0.23 | 4.25 | 0.31 | Standard |
| 4 | 3.81 | 4 | 0.04 | 4.03 | 0.05 | 4.08 | 0.08 | 4.09 | 0.08 | Standard |
| 5 | 4.3 | 5.25 | 0.89 | 5.94 | 2.68 | 4.76 | 0.21 | 4.62 | 0.1 | DDDDI & MOI |
| 6 | 4.9 | 5.44 | 0.3 | 5.43 | 0.29 | 5.63 | 0.53 | 5.39 | 0.24 | DDDDI & MOI |
| 7 | 3.38 | 5.47 | 4.37 | 5.43 | 4.22 | 5.5 | 4.48 | 5.47 | 4.4 | DDDI |
| 8 | 3.9 | 5.51 | 2.57 | 5.48 | 2.5 | 5.58 | 2.81 | 5.47 | 2.44 | DDDDI & MOI |
| 11 | NA | NA | NA | NA | NA | NA | NA | NA | NA | NA |
| 12 | 5.2 | 6.52 | 1.75 | 6.42 | 1.48 | 6.49 | 1.67 | 6.1 | 0.81 | DDDDI & MOI |
| 20 | 3.46 | 4.9 | 2.07 | 4.94 | 2.16 | 4.64 | 1.38 | 4.84 | 1.88 | MOI |
| 21 | 5.08 | 6.48 | 1.98 | 6.34 | 1.59 | 6.41 | 1.77 | 6.21 | 1.29 | DDDDI & MOI |
| 22 | 3.2 | 4.73 | 2.33 | 4.75 | 2.41 | 4.7 | 2.25 | 4.77 | 2.45 | MOI |
| 23 | 4.08 | 4.46 | 0.15 | 4.48 | 0.17 | 4.43 | 0.12 | 4.7 | 0.38 | MOI |
| 24 | NA | NA | NA | NA | NA | NA | NA | NA | NA | NA |
| 25 | 3.57 | 5.13 | 2.43 | 5.13 | 2.44 | 5.14 | 2.45 | 5.15 | 2.48 | Standard |
| 26 | 4.54 | 5.48 | 0.89 | 5.6 | 1.14 | 5.68 | 1.32 | 5.7 | 1.35 | Standard |
| 27 | 3.7 | 6.38 | 7.18 | 6.32 | 6.83 | 6.4 | 7.26 | 6.26 | 6.56 | DDDDI & MOI |
| 28 | 3.09 | 5.29 | 4.84 | 5.37 | 5.21 | 5.37 | 5.24 | 5.36 | 5.16 | Standard |
| 29 | 4.32 | 5.24 | 0.85 | 5.31 | 0.98 | 5.23 | 0.83 | 5.28 | 0.93 | MOI |
| 31 | 2.69 | 4.49 | 3.23 | 3.96 | 1.6 | 4.12 | 2.04 | 4.36 | 2.77 | DDDI |
| 32 | 3.45 | 3.23 | 0.05 | 3.28 | 0.03 | 3.37 | 0.01 | 3.48 | 0 | DDDDI & MOI |
| 33 | NA | NA | NA | NA | NA | NA | NA | NA | NA | NA |
| 34 | NA | NA | NA | NA | NA | NA | NA | NA | NA | NA |
| 37 | 3.02 | 4.1 | 1.15 | 4.07 | 1.1 | 4.19 | 1.35 | 3.93 | 0.82 | DDDDI & MOI |
| 40 | NA | NA | NA | NA | NA | NA | NA | NA | NA | NA |
| 41 | NA | NA | NA | NA | NA | NA | NA | NA | NA | NA |
| 42 | 5.29 | 6.16 | 0.76 | 6.24 | 0.92 | 6.22 | 0.87 | 6.23 | 0.9 | Standard |
| 44 | 3.78 | 4.3 | 0.27 | 4.32 | 0.29 | 4.39 | 0.37 | 4.35 | 0.32 | Standard |
| 46 | 4.77 | 4.97 | 0.04 | 5 | 0.05 | 5.01 | 0.06 | 5.04 | 0.07 | Standard |
| 48 | 5.22 | 6.63 | 1.97 | 6.76 | 2.37 | 6.74 | 2.31 | 6.67 | 2.09 | Standard |
| 49 | 4.8 | 4.96 | 0.03 | 4.99 | 0.04 | 5 | 0.04 | 5.01 | 0.05 | Standard |
| 52 | 5.34 | 5.42 | 0.01 | 5.61 | 0.07 | 5.42 | 0.01 | 5.55 | 0.04 | Standard |
| 55 | 4.01 | 5.21 | 1.44 | 5.26 | 1.56 | 5.29 | 1.62 | 5.26 | 1.56 | Standard |
| 57 | 4.6 | 5.47 | 0.76 | 5.5 | 0.82 | 5.54 | 0.88 | 5.49 | 0.8 | Standard |
| 58 | 5.03 | 6.13 | 1.21 | 6.23 | 1.44 | 6.13 | 1.2 | 6.22 | 1.42 | MOI |
| 59 | 4.8 | 5.21 | 0.17 | 5.2 | 0.17 | 5.23 | 0.19 | 5.21 | 0.17 | DDDI |
| 61 | 3.71 | 4.28 | 0.33 | 4.25 | 0.3 | 4.27 | 0.31 | 4.26 | 0.3 | DDDI |
| 62 | 4.93 | 5.47 | 0.29 | 5.5 | 0.33 | 5.56 | 0.4 | 5.57 | 0.41 | Standard |
| 64 | 4.41 | 4.81 | 0.16 | 4.81 | 0.16 | 4.82 | 0.17 | 4.8 | 0.15 | DDDDI & MOI |
| 65 | 4.09 | 5.37 | 1.64 | 4.85 | 0.58 | 5.35 | 1.58 | 4.81 | 0.52 | DDDDI & MOI |
| 67 | 5.08 | 5.76 | 0.47 | 5.77 | 0.48 | 5.72 | 0.42 | 5.75 | 0.45 | MOI |
| 71 | 5.63 | 6.61 | 0.96 | 6.62 | 0.97 | 6.69 | 1.11 | 6.6 | 0.93 | DDDDI & MOI |
| 73 | 4.33 | 6.14 | 3.27 | 6.38 | 4.23 | 6.53 | 4.86 | 5.97 | 2.71 | DDDDI & MOI |
| Mean | 4.246 | 5.266 | 1.466 | 5.285 | 1.495 | 5.284 | 1.506 | 5.242 | 1.369 | NA |
| Median | 4.3 | 5.29 | 0.89 | 5.37 | 0.98 | 5.35 | 1.11 | 5.28 | 0.82 | NA |
| IQR | 1.2 | 0.88 | 1.8 | 0.96 | 2.08 | 1.02 | 1.81 | 0.95 | 1.79 | NA |
