# Supplementary material for: Modeling dynamics of acute HIV infection incorporating density-dependent cell death and multiplicity of infection
Source: PLoS Comput Biol. 2024 Jun 7;20(6):e1012129. doi: 10.1371/journal.pcbi.1012129 (PMC11189221; doi:10.1371/journal.pcbi.1012129)
Supplement: S11 Table — Data-derived peak magnitude and model-derived peak magnitude for each study participants, along with the squared difference for the data-and-model derived peak magnitude for each model. (DOCX) [file pcbi.1012129.s013.docx]

Table S11: Data-derived peak magnitude and model-derived peak magnitude for each study participants, along with the squared difference for the data-and-model derived peak magnitude for each model. We also report mean, median and interquartile range (IQR) for the reader reference.

| **ID** | **Data Peak magnitude** | **Standard Peak magnitude** | **Error Standard** | **DDDI**  **Peak magnitude** | **Error DDDI** | **MOI Peak magnitude** | **SQ_MOI** | **DDDDI & MOI Peak magnitude** | **Error DDDDI & MOI** | **Best Model** |
| --- | --- | --- | --- | --- | --- | --- | --- | --- | --- | --- |
| 1 | 7.51 | 6.99 | 0.2708 | 7.1 | 0.17 | 7.06 | 0.204 | 7.06 | 0.2005 | DDDI |
| 2 | 5.71 | 5.41 | 0.0884 | 5.47 | 0.0552 | 5.4 | 0.0961 | 5.5 | 0.045 | DDDDI & MOI |
| 4 | 5.94 | 5.22 | 0.5122 | 5.28 | 0.4361 | 5.4 | 0.2923 | 5.39 | 0.2968 | MOI |
| 5 | 5.74 | 6.74 | 0.9936 | 7.3 | 2.4102 | 5.97 | 0.0504 | 5.7 | 0.0022 | DDDDI & MOI |
| 6 | 7.27 | 6.76 | 0.2504 | 6.8 | 0.2206 | 7.04 | 0.0516 | 6.74 | 0.2752 | MOI |
| 7 | 6.78 | 6.78 | 2.5E-8 | 6.72 | 0.0039 | 6.85 | 0.0042 | 6.8 | 4E-04 | Standard |
| 8 | 7.25 | 6.85 | 0.1573 | 6.81 | 0.1892 | 7 | 0.0618 | 6.8 | 0.1955 | MOI |
| 11 | 7.78 | 7.98 | 0.0377 | 7.82 | 0.0012 | 8.26 | 0.233 | 7.4 | 0.148 | DDDI |
| 12 | 8.07 | 7.97 | 0.0098 | 7.88 | 0.0368 | 8.06 | 3E-04 | 7.52 | 0.3049 | MOI |
| 20 | 6.68 | 6.32 | 0.1293 | 6.39 | 0.0852 | 5.96 | 0.5221 | 6.28 | 0.1564 | DDDI |
| 21 | 8.27 | 7.93 | 0.1181 | 7.73 | 0.2899 | 7.87 | 0.1593 | 7.67 | 0.3559 | Standard |
| 22 | 6.32 | 6.16 | 0.0246 | 6.2 | 0.0131 | 6.18 | 0.0196 | 6.22 | 0.0095 | DDDDI & MOI |
| 23 | 6.26 | 5.8 | 0.2108 | 5.84 | 0.1748 | 5.77 | 0.242 | 6.08 | 0.0336 | DDDDI & MOI |
| 24 | 6.01 | 5.69 | 0.1034 | 5.67 | 0.1149 | 5.68 | 0.1067 | 5.57 | 0.1954 | Standard |
| 25 | 6.74 | 6.55 | 0.0383 | 6.55 | 0.0369 | 6.59 | 0.024 | 6.61 | 0.0161 | DDDDI & MOI |
| 26 | 7.39 | 6.75 | 0.4078 | 6.9 | 0.24 | 7.12 | 0.0745 | 7.15 | 0.0607 | DDDDI & MOI |
| 27 | 8.29 | 7.84 | 0.2078 | 7.79 | 0.2496 | 7.89 | 0.1591 | 7.76 | 0.2846 | MOI |
| 28 | 7.07 | 6.71 | 0.1283 | 6.82 | 0.0642 | 6.81 | 0.0699 | 6.83 | 0.0571 | DDDDI & MOI |
| 29 | 7.01 | 6.5 | 0.2626 | 6.58 | 0.1895 | 6.51 | 0.249 | 6.58 | 0.1875 | DDDDI & MOI |
| 31 | 5.68 | 6 | 0.1043 | 5.35 | 0.1068 | 5.58 | 0.0106 | 5.93 | 0.0606 | MOI |
| 32 | 4.84 | 4.44 | 0.1532 | 4.48 | 0.1273 | 4.56 | 0.0781 | 4.58 | 0.0634 | DDDDI & MOI |
| 33 | 5.64 | 5.36 | 0.0805 | 5.41 | 0.0549 | 5.48 | 0.025 | 5.51 | 0.017 | DDDDI & MOI |
| 34 | 7.24 | 7.3 | 0.0044 | 7.21 | 7E-04 | 7.32 | 0.006 | 6.87 | 0.135 | DDDI |
| 37 | 5.45 | 5.16 | 0.0814 | 5.15 | 0.0882 | 5.32 | 0.0157 | 4.9 | 0.302 | MOI |
| 40 | 7.76 | 7.71 | 0.0023 | 7.17 | 0.3442 | 7.84 | 0.0072 | 7.72 | 0.0016 | DDDDI & MOI |
| 41 | 7.69 | 7.65 | 0.0019 | 7.42 | 0.0721 | 7.44 | 0.0629 | 7.47 | 0.0476 | Standard |
| 42 | 7.67 | 7.41 | 0.0686 | 7.47 | 0.0418 | 7.55 | 0.0152 | 7.53 | 0.0194 | MOI |
| 44 | 5.95 | 5.53 | 0.1799 | 5.57 | 0.1463 | 5.73 | 0.0471 | 5.59 | 0.1291 | MOI |
| 46 | 6.53 | 6.14 | 0.1555 | 6.2 | 0.1106 | 6.18 | 0.1226 | 6.25 | 0.0783 | DDDDI & MOI |
| 48 | 8.54 | 8.01 | 0.2802 | 8.12 | 0.1725 | 8.24 | 0.0856 | 8.12 | 0.1695 | MOI |
| 49 | 6.46 | 6.27 | 0.0369 | 6.28 | 0.0334 | 6.35 | 0.0121 | 6.36 | 0.0118 | DDDDI & MOI |
| 52 | 6.66 | 6.56 | 0.0104 | 6.68 | 6E-04 | 6.62 | 0.0018 | 6.84 | 0.034 | DDDI |
| 55 | 6.79 | 6.55 | 0.0616 | 6.59 | 0.0399 | 6.69 | 0.0107 | 6.61 | 0.0339 | MOI |
| 57 | 6.86 | 6.74 | 0.0128 | 6.8 | 0.0032 | 6.9 | 0.002 | 6.8 | 0.0032 | MOI |
| 58 | 7.69 | 7.48 | 0.0429 | 7.53 | 0.0244 | 7.46 | 0.051 | 7.69 | 3.1E-5 | DDDDI & MOI |
| 59 | 6.77 | 6.51 | 0.0689 | 6.48 | 0.0875 | 6.6 | 0.0283 | 6.49 | 0.0805 | MOI |
| 61 | 6.14 | 5.59 | 0.3029 | 5.57 | 0.3225 | 5.62 | 0.27 | 5.58 | 0.3142 | MOI |
| 62 | 7.13 | 6.92 | 0.043 | 6.96 | 0.0278 | 7.1 | 8E-04 | 7 | 0.0164 | MOI |
| 64 | 6.09 | 5.77 | 0.1053 | 5.75 | 0.1136 | 5.81 | 0.0808 | 5.74 | 0.1196 | MOI |
| 65 | 6.95 | 6.4 | 0.3055 | 5.87 | 1.1796 | 6.42 | 0.288 | 6 | 0.9118 | MOI |
| 67 | 7.16 | 7.08 | 0.007 | 7.03 | 0.018 | 7.07 | 0.0093 | 7.03 | 0.0165 | Standard |
| 71 | 8.21 | 7.97 | 0.0605 | 7.94 | 0.0731 | 8.09 | 0.0142 | 7.96 | 0.061 | MOI |
| 73 | 7.89 | 7.57 | 0.0998 | 7.78 | 0.0122 | 8.14 | 0.0663 | 7.46 | 0.1803 | DDDI |
| Mean | 6.881 | 6.63 | 0.145 | 6.615 | 0.19 | 6.687 | 0.091 | 6.597 | 0.131 | NA |
| Median | 6.86 | 6.71 | 0.1 | 6.72 | 0.088 | 6.69 | 0.052 | 6.74 | 0.063 | NA |
| IQR | 1.39 | 1.285 | 0.156 | 1.4 | 0.147 | 1.495 | 0.102 | 1.31 | 0.173 | NA |
