# Supplementary material for: Modeling dynamics of acute HIV infection incorporating density-dependent cell death and multiplicity of infection
Source: PLoS Comput Biol. 2024 Jun 7;20(6):e1012129. doi: 10.1371/journal.pcbi.1012129 (PMC11189221; doi:10.1371/journal.pcbi.1012129)
Supplement: S12 Table — Data-derived peak time and model-derived peak time for each study participants, along with the squared difference for the data-and-model derived peak time for each model. (DOCX) [file pcbi.1012129.s014.docx]

Table S12: Data-derived peak time and model-derived peak time for each study participants, along with the squared difference for the data-and-model derived peak time for each model. We also report mean, median and interquartile range (IQR) for the reader reference.

| **ID** | **Data peak time** | **Standard peak time** | **Error Standard** | **DDDI peak time** | **SQ_DDDI** | **MOI peak time** | **Error MOI** | **DDDDI & MOI peak time** | **Error DDDDI & MOI** | **Best Model** |
| --- | --- | --- | --- | --- | --- | --- | --- | --- | --- | --- |
| 1 | 11 | 9.03 | 3.881 | 14.08 | 9.49 | 11.47 | 0.221 | 11.63 | 0.397 | MOI |
| 2 | 7.41 | 6.27 | 1.292 | 8.74 | 1.78 | 8.99 | 2.507 | 9.31 | 3.623 | Standard |
| 4 | 18.91 | 15.95 | 8.783 | 17.84 | 1.15 | 17.9 | 1.027 | 18.29 | 0.389 | DDDDI & MOI |
| 5 | 12.17 | 2.47 | 94.183 | 0.1 | 145.8 | 4.43 | 59.982 | 11.77 | 0.164 | DDDDI & MOI |
| 6 | 11.34 | 9.1 | 5.01 | 10.32 | 1.04 | 9.81 | 2.336 | 9.94 | 1.955 | DDDI |
| 7 | 9.28 | 8.7 | 0.337 | 10.02 | 0.55 | 10.37 | 1.187 | 10.49 | 1.463 | Standard |
| 8 | 9.83 | 10.24 | 0.165 | 12.72 | 8.33 | 10.72 | 0.785 | 12.05 | 4.912 | Standard |
| 11 | 14.01 | 12.76 | 1.552 | 14.04 | 0.001 | 12.23 | 3.153 | 14.54 | 0.285 | DDDI |
| 12 | 12.92 | 11.49 | 2.043 | 13.15 | 0.05 | 10.67 | 5.06 | 11.81 | 1.231 | DDDI |
| 20 | 13 | 7.62 | 28.944 | 10.61 | 5.71 | 9.99 | 9.06 | 10.15 | 8.123 | DDDI |
| 21 | 12.12 | 11.65 | 0.217 | 12.46 | 0.12 | 12.04 | 0.006 | 12.23 | 0.013 | MOI |
| 22 | 8.1 | 5.91 | 4.781 | 8.98 | 0.78 | 7.74 | 0.127 | 8.3 | 0.041 | DDDDI & MOI |
| 23 | 11.03 | 8.01 | 9.134 | 11.51 | 0.23 | 11.17 | 0.019 | 11.63 | 0.357 | MOI |
| 24 | 22 | 28.95 | 48.303 | 27.06 | 25.6 | 22.98 | 0.96 | 22.27 | 0.073 | DDDDI & MOI |
| 25 | 8.93 | 10.35 | 2.018 | 10.44 | 2.28 | 10.87 | 3.766 | 11.02 | 4.371 | Standard |
| 26 | 10.52 | 9.74 | 0.608 | 11.9 | 1.9 | 11.51 | 0.98 | 11.38 | 0.74 | Standard |
| 27 | 13.89 | 13.01 | 0.773 | 14.39 | 0.25 | 13.25 | 0.408 | 13.01 | 0.773 | DDDI |
| 28 | 12.63 | 11.02 | 2.589 | 12.84 | 0.04 | 12.05 | 0.335 | 11.94 | 0.475 | DDDI |
| 29 | 12.85 | 8.33 | 20.4 | 11.15 | 2.88 | 10.38 | 6.084 | 10.36 | 6.183 | DDDI |
| 31 | 9 | 5.72 | 10.758 | 7.79 | 1.46 | 6.13 | 8.237 | 6.71 | 5.244 | DDDI |
| 32 | 10.72 | 6.26 | 19.858 | 10.87 | 0.02 | 10.36 | 0.127 | 10.93 | 0.046 | DDDI |
| 33 | 8 | 8.2 | 0.04 | 8.63 | 0.4 | 9.39 | 1.932 | 9.59 | 2.528 | Standard |
| 34 | 10 | 10.97 | 0.934 | 11.1 | 1.2 | 10.91 | 0.821 | 11.26 | 1.578 | MOI |
| 37 | 17 | 10.19 | 46.376 | 15.52 | 2.19 | 14.9 | 4.41 | 6.23 | 115.993 | DDDI |
| 40 | 9 | 7.39 | 2.592 | 7.72 | 1.64 | 7.58 | 2.016 | 7.68 | 1.742 | DDDI |
| 41 | 12.55 | 11.26 | 1.666 | 13.01 | 0.21 | 12.01 | 0.292 | 11.98 | 0.326 | DDDI |
| 42 | 11.54 | 11.39 | 0.023 | 11.75 | 0.04 | 12.55 | 1.018 | 12.73 | 1.414 | Standard |
| 44 | 12.68 | 10.1 | 6.658 | 13.97 | 1.66 | 13.21 | 0.281 | 12.93 | 0.062 | DDDDI & MOI |
| 46 | 15.74 | 11.13 | 21.272 | 15.38 | 0.13 | 14.23 | 2.287 | 14.35 | 1.938 | DDDI |
| 48 | 9.56 | 8.18 | 1.903 | 8.75 | 0.66 | 8.81 | 0.562 | 8.36 | 1.439 | MOI |
| 49 | 11.53 | 11.86 | 0.11 | 12.26 | 0.54 | 12.96 | 2.052 | 13.91 | 5.675 | Standard |
| 52 | 11 | 9.26 | 3.028 | 9.97 | 1.06 | 10.64 | 0.13 | 17.19 | 38.316 | MOI |
| 55 | 9.47 | 9.78 | 0.098 | 11.82 | 5.54 | 10.62 | 1.331 | 10.66 | 1.425 | Standard |
| 57 | 10.39 | 9.57 | 0.671 | 13.58 | 10.18 | 11.77 | 1.906 | 12.65 | 5.111 | Standard |
| 58 | 10.39 | 9.61 | 0.612 | 10.78 | 0.15 | 11.4 | 1.015 | 11.24 | 0.718 | DDDI |
| 59 | 12.16 | 11.36 | 0.635 | 13.43 | 1.62 | 11.85 | 0.094 | 12.78 | 0.388 | MOI |
| 61 | 17.4 | 13.6 | 14.427 | 16.75 | 0.42 | 15.53 | 3.491 | 15.98 | 2.012 | DDDI |
| 62 | 9.51 | 10.32 | 0.662 | 12.81 | 10.91 | 11.55 | 4.175 | 12.33 | 7.971 | Standard |
| 64 | 18 | 15.52 | 6.15 | 19.96 | 3.84 | 18.56 | 0.314 | 19.58 | 2.496 | MOI |
| 65 | 7.3 | 8.82 | 2.323 | 7.73 | 0.19 | 11.69 | 19.307 | 5.23 | 4.268 | DDDI |
| 67 | 8.13 | 8.59 | 0.21 | 10.77 | 6.96 | 9.7 | 2.46 | 10.24 | 4.445 | Standard |
| 71 | 9.03 | 9.48 | 0.2 | 11.58 | 6.49 | 10.07 | 1.076 | 10.88 | 3.413 | Standard |
| 73 | 9.88 | 9.88 | 2.76E-7 | 11.06 | 1.39 | 11.26 | 1.906 | 11.93 | 4.205 | Standard |
| Mean | 11.673 | 10.21 | 8.749 | 12.078 | 6.207 | 11.541 | 3.703 | 11.848 | 5.775 | NA |
| Median | 11 | 9.78 | 2.018 | 11.75 | 1.2 | 11.26 | 1.187 | 11.63 | 1.578 | NA |
| IQR | 3.275 | 2.85 | 7.11 | 3.125 | 3.12 | 1.925 | 2.458 | 2.455 | 3.926 | NA |
