# Supplementary material for: Modeling dynamics of acute HIV infection incorporating density-dependent cell death and multiplicity of infection
Source: PLoS Comput Biol. 2024 Jun 7;20(6):e1012129. doi: 10.1371/journal.pcbi.1012129 (PMC11189221; doi:10.1371/journal.pcbi.1012129)
Supplement: S13 Table — Data-derived joint peak measurement and model-derived joint peak measurement for each study participants, along with the squared difference for the data-and-model derived joint peak measurement for each model. (DOCX) [file pcbi.1012129.s015.docx]

Table S13: Data-derived joint peak measurement and model-derived joint peak measurement for each study participants, along with the squared difference for the data-and-model derived joint peak measurement for each model. The joint measurement for the peak is defined as $\frac{peak magnitude-mean peak magnitude}{mean peak magnitude}$ +$\frac{peak timing-mean peak timing}{mean peak timing}$ . We also report mean, median and interquartile range (IQR) for the reader reference.

| **ID** | **Data peak joint** | **Standard peak joint** | **Error Standard** | **DDDI peak joint** | **Error DDDI** | **MOI**  **peak joint** | **Error MOI** | **DDDDI & MOI peak joint** | **Error DDDDI & MOI** | **Best Model** |
| --- | --- | --- | --- | --- | --- | --- | --- | --- | --- | --- |
| 1 | 2.03 | 1.94 | 0.009 | 2.24 | 0.042 | 2.05 | 2E-04 | 2.05 | 3E-04 | MOI |
| 2 | 1.46 | 1.43 | 0.0011 | 1.55 | 0.0076 | 1.59 | 0.0149 | 1.62 | 0.0239 | Standard |
| 4 | 2.48 | 2.35 | 0.0178 | 2.27 | 0.0435 | 2.36 | 0.0157 | 2.36 | 0.015 | DDDDI & MOI |
| 5 | 1.88 | 1.26 | 0.3833 | 1.11 | 0.5877 | 1.28 | 0.3616 | 1.86 | 4E-04 | DDDDI & MOI |
| 6 | 2.03 | 1.91 | 0.0134 | 1.88 | 0.0212 | 1.9 | 0.0155 | 1.86 | 0.0278 | Standard |
| 7 | 1.78 | 1.88 | 0.0089 | 1.85 | 0.0042 | 1.92 | 0.0201 | 1.92 | 0.0184 | DDDI |
| 8 | 1.9 | 2.04 | 0.0198 | 2.08 | 0.035 | 1.98 | 0.0064 | 2.05 | 0.0233 | MOI |
| 11 | 2.33 | 2.45 | 0.0149 | 2.34 | 2E-04 | 2.3 | 0.0012 | 2.35 | 3E-04 | DDDI |
| 12 | 2.28 | 2.33 | 0.0023 | 2.28 | 0 | 2.13 | 0.0227 | 2.14 | 0.0206 | DDDI |
| 20 | 2.08 | 1.7 | 0.1481 | 1.84 | 0.0578 | 1.76 | 0.1076 | 1.81 | 0.0758 | DDDI |
| 21 | 2.24 | 2.34 | 0.0094 | 2.2 | 0.0016 | 2.22 | 4E-04 | 2.2 | 0.002 | MOI |
| 22 | 1.61 | 1.51 | 0.0107 | 1.68 | 0.0048 | 1.59 | 3E-04 | 1.64 | 0.001 | MOI |
| 23 | 1.86 | 1.66 | 0.0382 | 1.84 | 4E-04 | 1.83 | 6E-04 | 1.9 | 0.0023 | DDDI |
| 24 | 2.76 | 3.69 | 0.8746 | 3.1 | 0.1153 | 2.84 | 0.0069 | 2.72 | 0.0012 | DDDDI & MOI |
| 25 | 1.74 | 2 | 0.0657 | 1.85 | 0.012 | 1.93 | 0.0332 | 1.93 | 0.0353 | DDDI |
| 26 | 1.98 | 1.97 | 8.46E-6 | 2.03 | 0.0028 | 2.06 | 0.0075 | 2.04 | 0.0046 | Standard |
| 27 | 2.39 | 2.46 | 0.0038 | 2.37 | 7E-04 | 2.33 | 0.0044 | 2.27 | 0.0147 | DDDI |
| 28 | 2.11 | 2.09 | 3E-04 | 2.09 | 3E-04 | 2.06 | 0.0023 | 2.04 | 0.0044 | DDDI |
| 29 | 2.12 | 1.8 | 0.1046 | 1.92 | 0.0409 | 1.87 | 0.0606 | 1.87 | 0.0616 | DDDI |
| 31 | 1.6 | 1.47 | 0.0171 | 1.45 | 0.0203 | 1.37 | 0.0535 | 1.46 | 0.0174 | Standard |
| 32 | 1.62 | 1.28 | 0.1138 | 1.58 | 0.0019 | 1.58 | 0.0017 | 1.62 | 1.31E-5 | DDDDI & MOI |
| 33 | 1.51 | 1.61 | 0.0112 | 1.53 | 7E-04 | 1.63 | 0.0165 | 1.64 | 0.0194 | DDDI |
| 34 | 1.91 | 2.18 | 0.0715 | 2.01 | 0.0101 | 2.04 | 0.017 | 1.99 | 0.0068 | DDDDI & MOI |
| 37 | 2.25 | 1.78 | 0.2222 | 2.06 | 0.0341 | 2.09 | 0.026 | 1.27 | 0.9604 | MOI |
| 40 | 1.9 | 1.89 | 1E-04 | 1.72 | 0.0307 | 1.83 | 0.0047 | 1.82 | 0.0065 | Standard |
| 41 | 2.19 | 2.26 | 0.004 | 2.2 | 3.83E-5 | 2.15 | 0.0016 | 2.14 | 0.0024 | DDDI |
| 42 | 2.1 | 2.23 | 0.0168 | 2.1 | 4.11E-6 | 2.22 | 0.0127 | 2.22 | 0.0126 | DDDI |
| 44 | 1.95 | 1.82 | 0.0165 | 2 | 0.0022 | 2 | 0.0026 | 1.94 | 2E-04 | DDDDI & MOI |
| 46 | 2.3 | 2.02 | 0.0796 | 2.21 | 0.0076 | 2.16 | 0.0197 | 2.16 | 0.0194 | DDDI |
| 48 | 2.06 | 2.01 | 0.0026 | 1.95 | 0.0116 | 2 | 0.004 | 1.94 | 0.015 | Standard |
| 49 | 1.93 | 2.11 | 0.0326 | 1.96 | 0.0014 | 2.07 | 0.0214 | 2.14 | 0.0442 | DDDI |
| 52 | 1.91 | 1.9 | 2E-04 | 1.84 | 0.0055 | 1.91 | 2.04E-6 | 2.49 | 0.3339 | MOI |
| 55 | 1.8 | 1.95 | 0.0216 | 1.98 | 0.0314 | 1.92 | 0.015 | 1.9 | 0.0106 | DDDDI & MOI |
| 57 | 1.89 | 1.95 | 0.0046 | 2.15 | 0.0706 | 2.05 | 0.0274 | 2.1 | 0.0448 | Standard |
| 58 | 2.01 | 2.07 | 0.0038 | 2.03 | 5E-04 | 2.1 | 0.0092 | 2.11 | 0.0115 | DDDI |
| 59 | 2.03 | 2.09 | 0.0047 | 2.09 | 0.0043 | 2.01 | 1E-04 | 2.06 | 0.0013 | MOI |
| 61 | 2.38 | 2.17 | 0.0432 | 2.23 | 0.0236 | 2.19 | 0.0387 | 2.19 | 0.0356 | DDDI |
| 62 | 1.85 | 2.05 | 0.0417 | 2.11 | 0.0689 | 2.06 | 0.0451 | 2.1 | 0.0631 | Standard |
| 64 | 2.43 | 2.39 | 0.0014 | 2.52 | 0.009 | 2.48 | 0.0024 | 2.52 | 0.0092 | Standard |
| 65 | 1.64 | 1.83 | 0.0375 | 1.53 | 0.0118 | 1.97 | 0.1136 | 1.35 | 0.0813 | DDDI |
| 67 | 1.74 | 1.91 | 0.0294 | 1.95 | 0.0469 | 1.9 | 0.0255 | 1.93 | 0.0371 | MOI |
| 71 | 1.97 | 2.13 | 0.0265 | 2.16 | 0.0369 | 2.08 | 0.0134 | 2.13 | 0.025 | MOI |
| 73 | 1.99 | 2.11 | 0.0137 | 2.09 | 0.0097 | 2.19 | 0.0404 | 2.14 | 0.0211 | DDDI |
| Mean | 2 | 2 | 0.059 | 1.999 | 0.033 | 2 | 0.028 | 2 | 0.049 | NA |
| Median | 1.98 | 2 | 0.016 | 2.03 | 0.01 | 2.04 | 0.015 | 2.04 | 0.015 | NA |
| IQR | 0.3 | 0.325 | 0.036 | 0.335 | 0.033 | 0.24 | 0.023 | 0.275 | 0.028 | NA |
