# Supplementary material for: Modeling dynamics of acute HIV infection incorporating density-dependent cell death and multiplicity of infection
Source: PLoS Comput Biol. 2024 Jun 7;20(6):e1012129. doi: 10.1371/journal.pcbi.1012129 (PMC11189221; doi:10.1371/journal.pcbi.1012129)
Supplement: S36 Fig — We provide the fitted curves of the four models (solid lines) to the viral load measurements (points). The black line represents the Standard model, the green line shows the Density-dependent cell death model, the pink line shows the MOI model and orange line represents the Density-dependent cell death & MOI model. Time 0 is the day of the first positive result in the Aptima HIV-1 RNA Qualitative Assay and a quantitative viral load measurement was not taken at that day. (PDF) [file pcbi.1012129.s051.pdf]

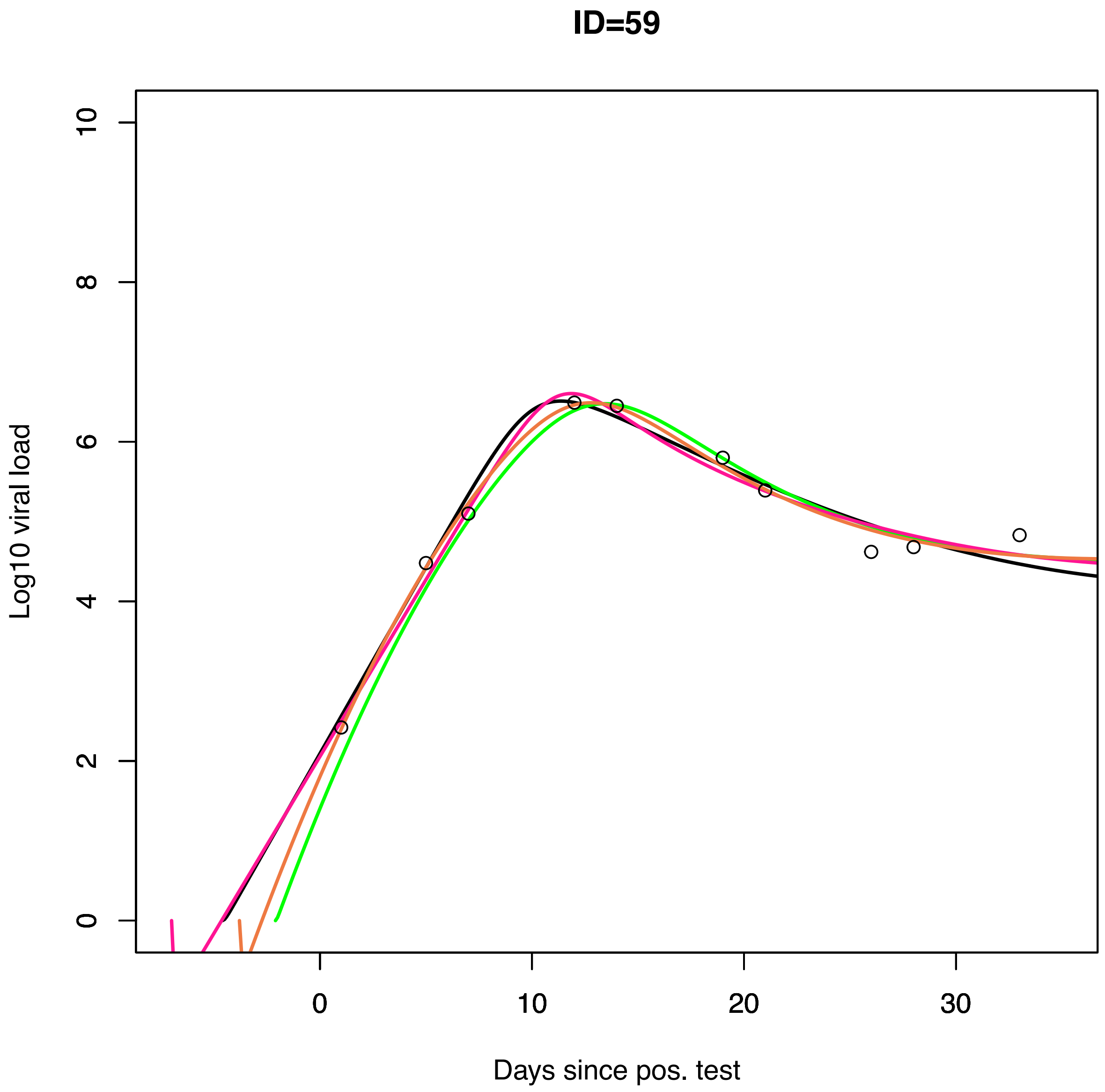

Fig S36: All model fits to study participant 59. We provide the fitted curves of the four models (solid lines) to the viral load measurements (points). The black line represents the Standard model, the green line shows the Density-dependent cell death model, the pink line shows the MOI model and orange line represents the Density-dependent cell death & MOI model. Time 0 is the day of the first positive result in the Aptima HIV-1 RNA Qualitative Assay and a quantitative viral load measurement was not taken at that day.
